# Mathematical model of the zebrafish ventricular cardiomyocyte action potential and calcium transient

**DOI:** 10.1113/JP287624

**Published:** 2025-07-31

**Authors:** Ludovica Cestariolo, Zachary D. Long, Arie O. Verkerk, Jose M. Ferrero, T. Alexander Quinn, Jose F. Rodriguez Matas

**Affiliations:** ^1^ Department of Biotechnology and Biosciences Università degli studi di Milano‐Bicocca Milan Italy; ^2^ Laboratory of Biological Structure Mechanics (LaBS), Department of Chemistry Materials and Chemical Engineering ‘Giulio Natta’ Politecnico di Milano Milan Italy; ^3^ Department of Physiology and Biophysics, Faculty of Medicine Dalhousie University Halifax Nova Scotia Canada; ^4^ Department of Medical Biology, Amsterdam Cardiovascular Sciences, Amsterdam UMC University of Amsterdam Amsterdam The Netherlands; ^5^ Department of Experimental Cardiology, Heart Center, Amsterdam Cardiovascular Sciences, Amsterdam UMC University of Amsterdam Amsterdam The Netherlands; ^6^ Centre for Research and Innovation in Bioengineering (Ci^2^B) Universitat Politècnica de València Valencia Spain; ^7^ School of Biomedical Engineering Dalhousie University Halifax Nova Scotia Canada

**Keywords:** cardiomyocyte, computational simulations, microelectrode recordings, optical mapping, patch clamp, restitution

## Abstract

**Abstract:**

In recent decades, the use of zebrafish to study cardiac electrophysiology has expanded significantly, based on striking similarities between zebrafish and human action potentials, as well as the underlying ion channels involved. Here, we developed a detailed mathematical model of the zebrafish ventricular cardiomyocyte action potential. The model is based on a previously developed human cardiomyocyte framework, with a simple calcium dynamics component that allows realistic modelling of calcium transients and excitation–contraction coupling in zebrafish. It was reparameterized using published patch clamp data and newly generated L‐type calcium current recordings from single cells to adjust the biophysical properties of the principal ionic currents. The principal ionic current conductances in the model were then calibrated and validated using new experimental data, including microelectrode measurements of membrane potential and optical measurements of intracellular calcium in isolated hearts during steady‐state and restitution pacing protocols. The model was used to explore components underlying the zebrafish action potential and calcium transient, highlighting that: (1) the T‐type calcium current contributes to the action potential upstroke; (2) the L‐type calcium current strongly affects the plateau and is a greater contributor to the intracellular calcium transient than sarcoplasmic reticulum calcium release; and (3) both rapid and slow delayed rectifier potassium currents make significant contributions to action potential repolarization. Overall, the novel zebrafish‐specific computational model presented here provides a valuable tool for studying cardiac electrophysiology in zebrafish and may be adapted in future work for use in large‐scale models to study whole heart electrical activity.

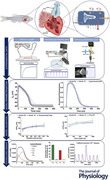

**Key points:**

- We have developed the first zebrafish‐specific computational ventricular action potential model, based on new and existing patch clamp data from single cells, with model calibration and validation performed using newly generated voltage and calcium measurements in the whole heart.
- The model reinforces experimental findings, highlighting key roles of T‐ and L‐type calcium currents in sustaining action potential depolarization and the intracellular calcium transient.
- Despite conflicting evidence regarding the existence of the slow delayed rectifier potassium current in zebrafish, the model suggested its important role in repolarization.
- While single‐cell and tissue model simulations produced similar results, depolarization‐related parameters (i.e. action potential upstroke speed and amplitude) varied, highlighting the importance of tissue‐based simulations for accurate comparison with tissue‐derived data.
- The model accurately predicted action potential prolongation with individual current block, aligning with experimental data. The effects of multi‐channel block were greater than in human, emphasizing the need for caution when translating zebrafish pharmacology.

## Graphical abstract

**Abstract figure legend** This study presents the development and validation of the first biophysically detailed computational model of the zebrafish ventricular action potential (AP). The model is based on a human cardiomyocyte framework and reparameterized using published and newly generated patch‐clamp recordings of zebrafish ionic currents. Model parameters were further calibrated using new experimental data, including microelectrode recordings of membrane potential and optical mapping of calcium transients (CaT) in adult zebrafish whole hearts. The model reproduces steady‐state AP and CaT morphologies, restitution dynamics, and responses to ionic current block. Comparative simulations and experimental data highlight the critical importance of calcium currents to the zebrafish AP and CaT. The model offers physiological insight into calcium handling and excitation–contraction coupling in zebrafish and provides a foundation for future whole‐heart computational simulations.

## Introduction

The zebrafish (*Danio rerio*) has emerged as a powerful experimental model for cardiac research, in part due to its remarkable similarities to human cardiac function and its high degree of genetic homology (Gut et al., 2017), with most zebrafish cardiac genes having human orthologues with analogous function (Howe et al., 2013). Zebrafish offer distinct advantages over traditional mammalian models, being more cost‐effective and requiring less intensive husbandry and experimental resources. Furthermore, the ease of introducing targeted genetic modifications facilitates the study of underlying pathways and mechanisms (Rafferty & Quinn, 2018). These specific advantages have driven the popularity of zebrafish in cardiac (patho‐)physiology research and high‐throughput pharmacological screening, positioning them as an essential tool for advancing our understanding of cardiovascular health and disease (González‐Rosa, 2022; Stoyek & Quinn, 2018).

The zebrafish is particularly useful for studies of cardiac electrophysiology, as resting heart rate, mechanisms of automaticity, sarcolemmal ion channels, action potential (AP) morphologies, calcium (Ca^2+^) handling proteins, and intra‐ and extracardiac regulatory control are comparable to human (MacDonald et al., 2017; Nemtsas et al., 2010; Stoyek et al., 2022). There are of course limitations to their use for the study of some aspects of cardiac physiology, as the zebrafish heart is composed of two chambers (one atrium and one ventricle, unlike the four‐chambered heart of most mammals), operates at relatively low pressures (Hu et al., 2001), and cardiomyocytes (CMs) in zebrafish also have poorly developed traverse tubules and ryanodine receptors that are less sensitive to Ca^2+^ than in mammals, such that even with much higher sarcoplasmic reticulum (SR) Ca^2+^ levels, the release of Ca^2+^ from the SR during excitation (via Ca^2+^‐induced Ca^2+^ release) is limited (Bovo et al., 2013; Brette et al., 2008), and partly driven by reverse‐mode sodium (Na^+^)–Ca^2+^ exchanger current, which is much higher than in mammals (Zhang et al., 2011). Also important to consider for studies involving genetic manipulation is the duplication of the zebrafish genome, which means that even though over 70% of zebrafish genes are orthologous to human, 24% of human genes have two orthologues in the zebrafish, leading to redundancy of gene function that can make interpretation of results difficult (Howe et al., 2013).

Yet, even with the popularity of the zebrafish as an experimental model for studies of cardiac electrophysiology (Ravens, 2018; van Opbergen et al., 2018; Vornanen & Hassinen, 2016), and the extensive use of biophysical computational AP models in the field (Trayanova et al., 2024), comprehensive, biophysically detailed computational models of the adult zebrafish AP are lacking, limiting the potential for generating novel mechanistic insight through combined experimental–computational investigations (Quinn & Kohl, 2013). To date, only phenomenological models are available, such as those utilized by Crowcombe et al. (2016), Qian and Tarte (2019), and Cestariolo, Luraghi, et al. (2023), or preliminary reports from our group describing the initial development of a full model, which importantly outline the transition from the ten Tusscher and Panfilov formulation of the human ventricular AP from 2006 (TP06) (Cestariolo et al., 2022; ten Tusscher & Panfilov, 2006) to the ten Tusscher and Panfilov previous formulation from 2004 (TP04) (Cestariolo, Ferrero, et al., 2023; ten Tusscher et al., 2004) as the base model, due to the simpler Ca^2+^ handling system of the TP04 formulation, and improved agreement with experimental data. This warrants the development of comprehensive, biophysically detailed computational zebrafish AP models to bolster the utility of the zebrafish as a model organism and to enhance the translation of findings to human.

Based on this need, the goal of the current study was to develop a 0D single‐cell biophysical computational model of the adult zebrafish ventricular AP that reproduces AP and intracellular calcium transient (CaT) morphologies at various stimulation rates, as well as their restitution properties. For this, the TP04 formulation was modified by reparameterizing the biophysical properties of the principal ionic currents (fast Na^+^ current, $I_{\mathrm{Na}}$; L‐type Ca^2+^ current, $I_{\mathrm{CaL}}$; rapid and slow delayed rectifier potassium (K^+^) currents, $I_{\mathrm{Kr}}$ and $I_{\mathrm{Ks}}$; the inward rectifier K^+^ current, $I_{K1}$) and adding the T‐type Ca^2+^ current, $I_{\mathrm{CaT}}$, using novel and published experimental patch‐clamp data, followed by calibration of their conductances using newly generated experimental measurements of transmembrane potential ($V_{m}$) and intracellular Ca^2+^ dynamics from the adult zebrafish isolated heart. This 0D single‐cell model was then incorporated into a 1D multicellular framework. The developed 0D and 1D models accurately reproduced the morphology of the AP and CaT in steady‐state conditions and experimentally measured AP (APD) and CaT (CaTD) duration restitution and were subsequently used to explore the mechanisms contributing to the CaT in zebrafish and responses to the pharmacological block of various ion currents, which agreed with previous experimental findings.

In summary, here we have developed the first computational model of the zebrafish ventricular AP, based on novel experimental findings, which will be a valuable tool for future translational studies of cardiac electrophysiology in health and disease.

## Methods

### Ethical approval

Patch‐clamp experiments using isolated zebrafish ventricular myocytes were performed at Amsterdam UMC (Amsterdam, The Netherlands) and were approved by the Dutch Central Authority for Scientific Procedures on Animals in accordance with the Utrecht Hubrecht Institute and Amsterdam UMC ethical guidelines. Zebrafish were sent from Brigham and Women's Hospital (Boston, MA, USA) and kept in quarantine at the Utrecht Hubrecht Institute. On the day of cell isolations, zebrafish were transported to Amsterdam UMC.

Microelectrode and intracellular Ca^2+^ experiments were performed at Dalhousie University (Halifax, Canada) and were approved by the Dalhousie University Committee for Laboratory Animals in accordance with the Canadian Council on Animal Care ethical guidelines. Zebrafish were maintained in standard 4–10 L tanks (nif–0000–31933; Aquatic Habitats, Apopka, FL, USA) at 28°C, with a continuous supply of conditioned water provided by a recirculating water system and a 14/10 h light/dark cycle. The fish were fed twice daily with commercial dry fish food (Golden Pearl pellets; Brine Shrimp Direct, Ogden, UT, USA) and live *Artemia* spp. cultured in‐house.

### Experimental preparation

#### Cell preparation

Zebrafish were killed by submersion in ice cold water for >1 min, until opercular movement stopped and there was no response to fin pinch, followed by excision of the heart. Ventricles from four or five adult wild‐type AB/Tuebingen (Tu) zebrafish of a mixed‐sex population were pooled and stored at room temperature in a HEPES‐buffered saline solution (in mM: 140 NaCl, 5.4 KCl, 1.8 CaCl_2_, 1 MgCl_2_, 5.5 glucose, 5 HEPES), with pH titrated to 7.4 using NaOH. Subsequently, the ventricles were cut into small pieces and transferred to HEPES‐buffered saline with 10 µM CaCl_2_ at 30°C. This solution was refreshed once, and single cells were dissociated by an enzymatic dissociation procedure as described previously (Chiang et al., 2024). The cells were stored at room temperature for at least 45 min in modified Kraft–Brühe solution (in mM: 85 KCl, 30 K_2_HPO4, 5 MgSO_4_, 5.5 glucose, 5 pyruvic acid, 5 creatine, 30 taurine, 5 β‐hydroxybutyric acid, 5 succinic acid, 1% bovine serum albumin, 2 Na_2_ATP), with pH titrated to 6.9 using KOH.

#### Whole heart preparation

In total, 18 adult wild‐type AB zebrafish (11–13 months post‐fertilization) from a mixed‐sex population were utilized for the whole heart experiments. Zebrafish were anaesthetized with 1.5 mm tricaine in Tris‐buffered (pH 7.4; BP152, Fisher Scientific, Hampton, NH, USA) 28°C tank water until opercular movement stopped and there was no response to fin pinch. The fish were then placed in a Sylgard‐lined dish (DC 170, Dow Corning, Midland, MI, USA) with HEPES‐buffered saline solution (in mM: 135 NaCl, 5 KCl, 5.5 NaHCO_3_, 1.5 NaH_2_PO_4_, 1.7 MgCl_2_, 1.8 CaCl_2_, 7.5 glucose, 5 creatine, 10 HEPES), with an osmolality of 300 ± 5 mOsm/kg and a pH of ∼7.30 ± 0.05 at 28°C. A ventral midline incision was made through the body wall and a block of tissue encompassing the ventral aorta, ventricle, atrium and sinus venosus was removed. The isolated heart was pinned with 100 mm pins (Fine Science Tools, Foster City, CA, USA) in a Sylgard‐lined Petri dish with 5 mL of the saline solution and maintained at 28.0 ± 0.5°C. Custom suction microelectrodes (1B100 and MPH6R10, World Precision Instruments, Sarasota, FL, USA) connected to an ECG amplifier (Animal Bio Amp, ADInstruments, Colorado Springs, CO, USA) were placed on the atrium and ventricle, and suction was applied with a 1 mL syringe to measure the ECG from each chamber for confirmation of successful pacing. The atria of hearts were paced at 2 Hz to control for heart rate (HR), using a square pulse stimulator (S48, Grass Instruments, Quincy, MA, USA) with a 2 ms pulse duration and current ranging from 20 to 100 mA. Bath temperature was measured using a thermocouple (T‐type pod, ADInstruments). Temperature and ECG signals were recorded at 2 kHz using a software‐controlled data acquisition system (PowerLab and LabChart, ADInstruments).

#### Cell patch‐clamp protocol

Isolated cells were placed in a recording chamber on the stage of an inverted microscope (Diaphot, Nikon) and superfused with HEPES‐buffered saline for $I_{\mathrm{CaL}}$ measurements.

$I_{\mathrm{CaL}}$ was recorded at 28°C with the amphotericin perforated patch clamp technique using an Axopatch 200B amplifier (Molecular Devices, Sunnyvale, CA, USA). Voltage control, data acquisition and signal analysis were performed with custom‐made software. Signals were filtered with a low pass filter with a cutoff of 5 kHz and digitized at 10 kHz. Patch pipettes (2.5–3.5 MΩ, borosilicate glass capillaries; Harvard Apparatus, Cambridge, UK) were filled with a solution containing (in mM): 125 potassium gluconate, 20 KCl, 5.0 NaCl, 0.55 amphotericin‐B, 10 HEPES; pH 7.2 (KOH). Cell membrane capacitance ($C_{m}$) was estimated by dividing the time constant of the decay of the capacitive transient in response to 5 mV hyperpolarizing voltage clamp steps from −40 mV by the series resistance and was calculated as 27.2 ± 8.8 pF (*n* = 6; presented as mean ± SD). For $I_{\mathrm{CaL}}$ measurements, $C_{m}$ and series resistance were compensated for by at least 60%.

#### Whole heart pacing protocols

Steady‐state, S1–S2 and dynamic restitution pacing protocols were performed. For the steady‐state pacing protocol, a train of 300 stimuli was delivered to the heart, with cycle lengths of 1000, 500 or 200 ms. For the S1–S2 restitution protocol, after a conditioning period of 10 stimuli at a cycle length of 500 ms (S1), a single premature stimulus of a shorter cycle length (S2) was delivered to the heart. The S1–S2 coupling interval was progressively shortened from 500 to 100 ms, with a varying step size (steps of 50 ms from 500 to 400 ms, 25 ms from 400 to 300 ms, 20 ms from 300 to 200 ms, and 10 ms from 200 to 100 ms). For the dynamic restitution protocol, trains of 100 stimuli were delivered to the heart, with a decreasing cycle length for each stimulus train (1000, 800, 600, 500, 400, 300, 250, 225, 200, 175 or 150 ms).

### *I*_CaL_ measurements

Current density and gating properties of $I_{\mathrm{CaL}}$ were recorded from a −60 mV holding potential using a two‐pulse voltage clamp protocol with a cycle length of 4 s (see Fig. 4A, top panel). The first pulse (300 ms with test potentials between −60 and +70 mV) served to activate $I_{\mathrm{CaL}}$, from which densities and voltage‐dependency of activation were analysed. The second pulse (200 ms to 0 mV) was used to analyse the voltage dependency of $I_{\mathrm{CaL}}$ inactivation. $I_{\mathrm{CaL}}$ was defined as the difference between peak current and steady‐state current (see Fig. 4A, bottom panel) and was normalized to $C_{m}$. Voltage dependence of activation and inactivation curves were fitted with a Boltzmann function ($y=\frac{1}{1+e^{\left(\frac{V_{m}-V_{1/2}}{k}\right)}}$), where $V_{1/2}$ is the half‐maximal voltage of (in)activation and k is the slope factor (mV). The time course of $I_{\mathrm{CaL}}$ inactivation was fitted with a mono‐exponential equation: $y=Ae^{\left(-\frac{t}{\tau }\right)}$. Data are presented as mean ± SD.

### AP measurements

Intracellular microelectrode recordings of $V_{m}$ were performed as previously described (Stoyek et al., 2024). Microelectrodes were pulled from borosilicate glass tubing (0.5 mm inner diameter, 1.0 mm outer diameter, with an internal filament; BF/100/50/10, Sutter Instruments, Novato, CA, USA) with a Brown/Flaming micropipette puller (P97, Sutter Instruments) and filled with 3 M KCl, resulting in a resistance of 40–60 MΩ. Electrodes were coupled to the headstage of an amplifier (1600 Neuroprobe Amplifier, A/M Systems, Everett, WA, USA) operated in the current clamp mode. The measured potential was taken as the difference between the potential inside the microelectrode and a silver/silver chloride wire in the bath. With the electrode tip in the bath, the potential was zeroed using the amplifier bridge controls. The tip of the electrode was then moved with a mechanical manipulator (MX/4, Narishige, Tokyo, Japan) into the mid‐ventricular/atrial epicardium until an AP signal was obtained. At the end of a recording, the microelectrode was withdrawn from the tissue, the null potential was checked, and the data were adjusted for shifts from zero, if necessary. Criteria for a successful cell impalement and data inclusion were an AP with a stable resting membrane potential (RMP) < −60 mV and a peak > 0 mV maintained throughout the recording. Data were recorded at 5 kHz using the data acquisition system.

The last 10 beats for each cycle length of the steady‐state and dynamic restitution pacing protocols, and the S2 beat of the S1–S2 restitution protocol, were used for AP analysis. RMP (stable $V_{m}$ during diastole), maximum AP upstroke speed (${dV/dt|}_{\max}$, maximum of the time derivative of $V_{m}$), time to peak $V_{m}$ ($t_{\mathrm{peak}}$, the time from RMP to maximum membrane potential, $V_{\max}$), AP amplitude (APA, the difference between $V_{\max}$ and RMP), and APD (time from the point of ${dV/dt|}_{\max}$ to a specified percentage decline in $V_{m}$ from peak to rest) were calculated and averaged over the beats in MATLAB (MathWorks, R2022a, Natick, MA, USA).

### Ca^2+^ transient measurements

Hearts were incubated with 0.02% pluronic acid (P2443, Sigma‐Aldrich, St Louis, MO, USA) in saline solution for 1 min. Ca^2+^‐sensitive fluorescent dye was added to the bath, along with 10 µM of the excitation–contraction uncoupler (±)‐blebbistatin (BB592490, Toronto Research Chemicals, Toronto, Canada), to minimize artefacts associated with movement during cardiac contraction. Two different Ca^2+^‐sensitive dyes with different affinities for Ca^2+^ were used: (1) 10 µM Rhod‐2 AM [ab142780, Abcam, Cambridge, UK; dissociation constant (*K*_d_) ≈ 0.57 µM) or (2) 5 µM Fluo‐5F AM (20560, AAT Bioquest, Pleasanton, CA, USA; *K*_d_ ≈ 2.30 µM), which were incubated for 15 or 20 min, respectively, followed by replacement with a saline solution containing 10 µM (±)‐blebbistatin and 1 mm probenecid (P36400, Fisher Scientific) and an additional 30 min period to allow for dye de‐esterification and the cessation of contraction.

For fluorescence imaging, hearts were epi‐illuminated by a mercury arc lamp (U‐HGLPS, Olympus, Tokyo, Japan) through a compound macroscope (MVX10, Olympus) with a 1× objective (MV PLAPO 1×, Olympus) at 6.3× magnification or an upright microscope (BX63, Olympus) with a 10× objective lens (MPlanFL N 10×, Olympus) for Rhod‐2 and Fluo‐5F, respectively. Rhod‐2 was excited for 5 s periods with light passed through a 525 ± 25 nm filter (FF03‐525/50, Semrock, Rochester, NY, USA) reflected by a 562 nm dichroic filter (FF562‐Di03, Semrock), with emitted fluorescence collected through a 578 ± 10.5 nm filter (FF01‐578/21, Semrock) and captured on an EMCCD camera with Solis software at 500 frames/s (iXon3, Andor, Belfast, UK). Fluo‐5F was excited for 5 s periods with light passed through a 466 ± 20 nm filter (F101‐466/40, Semrock) reflected by a 495 nm dichroic filter (FF495‐Di03, Semrock), with emitted fluorescence collected through a 574 ± 69 nm filter (FF01‐574/168, Semrock) and captured with the EMCCD camera and Solis software at 500 frames/s.

For analysis, a 10 × 10‐pixel region of interest was chosen at the mid‐ventricle. Ten consecutive CaT for each cycle length of the steady‐state or dynamic restitution pacing protocol or the S2 beat of the S1–S2 restitution protocol were used for CaT analysis. CaT upstroke speed (${dF/dt|}_{\max}$, maximum of the time derivative of scaled fluorescence) and CaTD (time from the point of ${dF/dt|}_{\max}$ to a specified percentage decline in fluorescence from peak to rest) were calculated and averaged over the beats in MATLAB (MathWorks, R2022a). Additionally, a second region of interest was chosen at a known distance from the first region to calculate conduction velocity based on the time delay between the respective upstrokes of the CaT signals.

Due to photobleaching that occurs with the use of Fluo‐5F, an abbreviated pacing protocol was employed to compare CaT measurements to Rhod‐2, involving a train of 100 stimuli with cycle lengths of 1000, 500 and 250 ms.

All experimental AP and intracellular Ca^2+^ transient data are presented as mean ± SD. Data are available upon request, and figures showing all individual data points are available on Figshare (DOI: https://doi.org/10.6084/m9.figshare.27997817).

### Computational model of the ventricular AP

**Figure 1:**
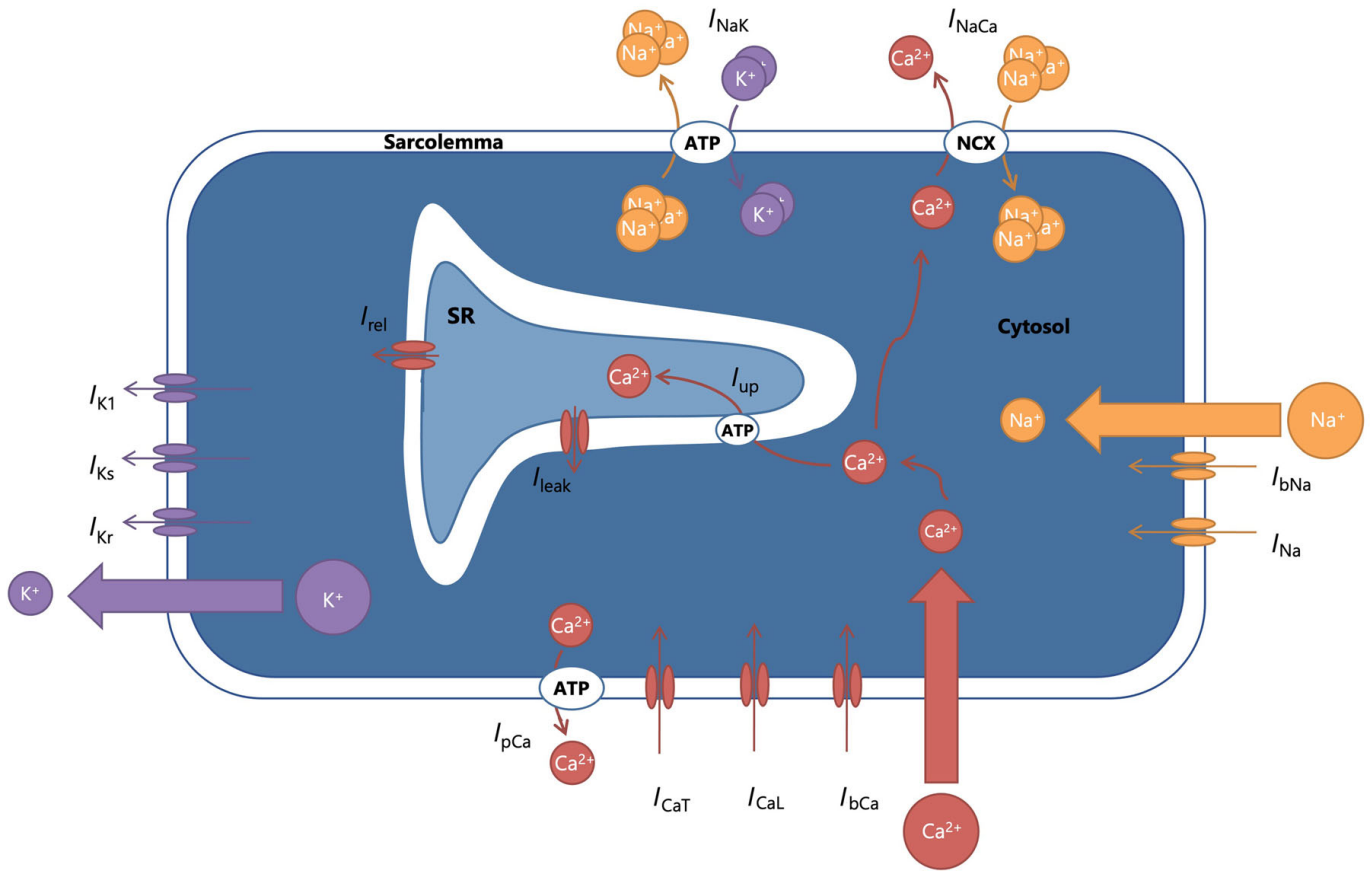
Schematic representation of the adult zebrafish ventricular CM On the sarcolemma, the ionic currents are: sodium (Na^+^) currents (fast Na^+^, $I_{\mathrm{Na}}$, and background Na^+^, $I_{\mathrm{bNa}}$, in yellow); calcium (Ca^2+^) currents (T‐type Ca^2+^, $I_{\mathrm{CaT}}$, L‐type Ca^2+^, $I_{\mathrm{CaL}}$, background Ca^2+^, $I_{\mathrm{bCa}}$, and sarcolemma Ca^2+^ pump, $I_{\mathrm{pCa}}$ in red); potassium (K^+^) currents (rapid delayed rectifier, $I_{\mathrm{Kr}}$, slow delayed rectifier, $I_{\mathrm{Ks}}$, and inward rectifier, $I_{K1},$ in purple). Additionally, the Na^+^–K^+^ pump ($I_{\mathrm{NaK}}$) and the Na^+^–Ca^2+^ exchanger ($I_{\mathrm{NaCa}}$). In the sarcoplasmic reticulum (SR), the currents include the uptake current ($I_{\mathrm{up}}$), the release current ($I_{\mathrm{rel}}$) and the leak current ($I_{\mathrm{leak}}$).

Figure 1 gives a schematic representation of the adult zebrafish ventricular CMs, illustrating the primary transmembrane currents and intracellular Ca^2+^ handling machinery. The computational model of the zebrafish ventricular AP developed in the current study was based on the TP04 formulation of the human ventricular AP (ten Tusscher et al., 2004), chosen for its high computational cost‐effectiveness and justified by the electrophysiological similarities between human and zebrafish. While newer human computational models exist, such as the updated ten Tusscher and Panfilov formulation (TP06; ten Tusscher & Panfilov, 2006) or the more recent O'Hara, Tomek and Bartolucci models (Bartolucci et al., 2020; O'Hara et al., 2011; Tomek et al., 2019), these incorporate more complex Ca^2+^ dynamics that were deemed unnecessary for modelling the zebrafish due to differences in its Ca^2+^ handling system compared to human. In fact, we first began developing our zebrafish ventricular AP model based on the TP06 formulation (Cestariolo et al., 2022) but later transitioned to the TP04 formulation (Cestariolo, Ferrero, et al., 2023) as its simpler Ca^2+^ handling system resulted in an improved agreement with experimental results.

As important differences between the ion channels of zebrafish and human ventricular CMs exist, the model was adjusted accordingly. Zebrafish ventricular CMs lack transient outward K^+^ channels (which pass $I_{\mathrm{to}}$) (Alday et al., 2014) and possess T‐type Ca^2+^ channels (which pass $I_{\mathrm{CaT}}$, and are almost undetectable in human) (Haverinen, Hassinen, Dash, et al., 2018), so $I_{\mathrm{to}}$ was removed from the model and $I_{\mathrm{CaT}}$ introduced [based on the formulation of Demir et al. (1994), for the rabbit sinoatrial node].

The TP04 model uses a parallel conductor model for the CMs, in which the membrane is modelled as a capacitor in parallel to non‐linear resistances and batteries, representing the ionic channels and current sources for modelling the pumps and exchangers. Therefore, the electrophysiological behaviour of the single CM (0D) is described as:

(1) $$\frac{dV_{m}}{dt}=-\frac{1}{C_{m}}\left(I_{\mathrm{ion}}+I_{\mathrm{stim}}\right),$$

where $C_{m}$ is the cell membrane capacitance, t is time, $I_{\mathrm{stim}}$ is the externally applied stimulation current (with an amplitude of −52 µA/µF and a 1 ms duration), and $I_{\mathrm{ion}}$ is the sum of the ionic currents:

(2) $$\begin{array}{ccc} I_{\mathrm{ion}} & = & I_{\mathrm{Na}}+I_{\mathrm{CaT}}+I_{\mathrm{CaL}}+I_{\mathrm{Ks}}+I_{\mathrm{Kr}}+I_{K1}+I_{\mathrm{NaK}}+I_{\mathrm{NaCa}}\\ & & +\;I_{\mathrm{pCa}}+I_{\mathrm{bNa}}+I_{\mathrm{bCa}}, \end{array}$$

where $I_{\mathrm{NaK}}$ is the Na^+^–K^+^ pump, $I_{\mathrm{NaCa}}$ the Na^+^–Ca^2+^ exchanger, $I_{\mathrm{pCa}}$ the sarcolemmal Ca^2+^ pump, and $I_{\mathrm{bNa}}$ and $I_{\mathrm{bCa}}$ the Na^+^ and Ca^2+^ background currents, respectively.

A one‐dimensional (1D) array of interconnected CMs can be modelled by the well‐known cable equation:

(3) $$\frac{\partial V_{m}}{\partial t}=D\frac{\partial^{2}V_{m}}{\partial x^{2}}-\frac{1}{C_{m}}\left(I_{\mathrm{ion}}+I_{\mathrm{stim}}\right),$$

where x represents space and D is the effective diffusion coefficient, proportional to the cable conductivity.

The physical units used in the model were: (1) ms for time, t; (2) mm for space, x; (3) mV for voltage, $V_{m}$; (4) µA/µF for ionic currents, $I_{x}$; (5) µF/cm^2^ for cell capacitance, $C_{m}$; (6) mS/µF for maximum ionic current conductances, $G_{x}$; (7) mmol/L for intracellular and extracellular ionic concentrations, ${\left[X\right]}_{i}$ and ${\left[X\right]}_{o}$.; and (8) mm^2^/ms for the effective diffusion coefficient, D.

### Model implementation

The model was implemented in MATLAB (MathWorks, R2022a). For the 0D (cell) simulations, a stiff solver (ODE 15s) with an adaptive time step was used. For the 1D (cable) simulations, eqn (3) was solved using the operator splitting numerical scheme together with the explicit Euler method. To integrate the Hodgkin–Huxley type equations for the gating variables of the various time‐dependent currents (m, h and j for $I_{\mathrm{Na}}$, $d_{T}$ and $f_{T}$ for $I_{\mathrm{CaT}}$, $d_{L}$, $f_{L}$, and $f_{\mathrm{Ca}}$ for $I_{\mathrm{CaL}}$, $x_{S}$ for $I_{\mathrm{Ks}}$, and $x_{r1}$ and $x_{r2}$ for $I_{\mathrm{Kr}}$, see ‘Sensitivity activity’ for more details) the Rush–Larsen scheme was used (Rush & Larsen, 1978). The parameters used for the computational simulations are reported in Appendix 2 and the scripts for the MATLAB models and simulations, along with a CellML version of the 0D model, are available on GitHub (https://github.com/jfrodrig1971/Zebrafish).

For the 1D simulations, a 0.99 mm long cable (compatible with the dimensions of the zebrafish ventricle) uniformly discretized with elements 0.03 mm in size was implemented with eqn (3), integrated using a constant time step of Δt=0.02ms. The effective diffusion coefficient was set to $D=1.76\;\times \;10^{-2}$ mm^2^/ms, resulting in a conduction velocity of 126.8 mm/s, which is consistent with our experimental measurements (125 ± 15 mm/s; *n* = 6) and conduction velocity reported in the literature (Kikuchi et al., 2010; Wang et al., 2011). The accuracy of the 1D computational simulations was verified against simulations that employed smaller space and time discretization of Δx=0.005mm and Δt=0.01ms, respectively. Results indicate that reducing the space discretization and time step to these values leads to an increase in the conduction velocity of less than 2% (from 127 to 129 mm/s).

First, the biophysical properties of the principal ionic currents were reparametrized using published and measured voltage‐clamp data from zebrafish (as described in‘Model ionic currents’ below). Next, to calibrate the ion channel conductances and Ca^2+^ handling parameters, a Monte Carlo simulation was conducted, involving variation of the maximum conductance of all currents and Ca^2+^ dynamics parameters to identify combinations that fit within the range of experimental data, while ensuring model stability. The protocol involved stimulating the model with 110 pulses at a frequency of 1 Hz, followed by a 3 s period without stimulation. The last 10 action potentials and the final 3 s of the simulation were saved for analysis. A given combination of parameters was considered valid if the results demonstrated, first, the absence of alternans in the last 10 action potentials and a lack of pacemaking behaviour following the cessation of stimulation, and then if AP features fell within the evaluated experimental ranges. The model turned out to be stable, and as a result, a large number of parameter combinations satisfied these conditions. Therefore, manual parameter tuning was performed to adjust the maximum conductance of the principal ionic currents and better match the model's restitution characteristics. This practice is commonly adopted in computational models due to the discrepancy between the current density measured in voltage‐clamp experiments and the apparent contribution of the current to APD (Varró et al., 2021). This inconsistency is probably attributable to the sensitivity of ion channels (in particular K^+^ channels), to the cell isolation procedures required for voltage‐clamp recordings, which can lead to substantial channel degradation prior to current density measurement (O'Hara et al., 2011; ten Tusscher et al., 2004; Tomek et al., 2019; Yue et al., 1996). The final conductance values are reported in Table A1 in Appendix 2.

Simulations were then run using the same stimulation protocols as for the experiments (described in‘Whole heart pacing protocols’ above) to validate the model, specifically: (1) a steady‐state protocol; (2) a dynamic restitution protocol; and (3) an S1–S2 restitution protocol. For the steady‐state protocol, the following AP characteristics of the last beat were compared with experimental recordings: (1) RMP; (2) ${dV/dt|}_{\max}$; (3) $V_{\max}$; (4) APA; (5) APD at 90% and 50% repolarization (APD_90_ and APD_50_ respectively); and (6) AP triangulation (APD_90_ − APD_50_), as well as characteristics of the CaT: (1) ${dF/dt|}_{\max}$; (2) time to peak Ca^2+^ ($t_{\mathrm{peak}}$); and (3) CaTD at 80% and 50% recovery (CaTD_80_ and CaTD_50_, respectively).

### Sensitivity analysis

After model parameterization and validation, a sensitivity analysis was conducted using both the 0D and 1D models, to evaluate the influence of each current on AP morphology. A Monte Carlo method was used, involving 5000 combinations of 18 parameters representing the maximum conductance of the principal currents ($G_{\mathrm{Na}}$, $G_{\mathrm{CaL}}$, $G_{\mathrm{CaT}}$, $G_{\mathrm{Kr}}$, $G_{\mathrm{Ks}}$, $G_{K1}$, $G_{\mathrm{pCa}}$, $G_{\mathrm{bNa}}$, $G_{\mathrm{bCa}}$, $G_{\mathrm{NaK}}$, $G_{\mathrm{NaCa}}$), the cytoplasmic volume ($V_{c}$), and key determinants of Ca^2+^ dynamics ($V_{\mathrm{leak}}$, $V_{\mathrm{mxu}}$, $K_{\mathrm{up}}$, $\tau_{g}$, $buf_{c}$, $kbuf_{c}$), which were varied within a range of ±30% with the exception of $V_{c}$, which was varied within a range of ±50%). Results were analysed by quantifying the unique relationship between each model parameter and the principal AP characteristics (i.e. RMP, APA, APD_20_, APD_50_, APD_80_, APD_90_, $dV/dt_{\max}$, $V_{\max}$, $t_{\mathrm{peak}}$ and AP triangulation) using partial correlation coefficients. This analysis was performed by controlling for the influence of all other parameters via a linear regression‐based approach to isolate the direct contribution of each parameter to the respective characteristics.

### Model response to channel specific block

Steady‐state APD_90_ was calculated in the presence of channel‐specific blockers and compared to values reported in the literature. The simulated block was based on experimental dose–response measurements in zebrafish hearts for the block of $I_{\mathrm{Kr}}$ with E4031 and $I_{\mathrm{Ks}}$ with chromanol 293B (Tsai et al., 2011), and the simultaneous block of $I_{\mathrm{Na}}$, $I_{\mathrm{Kr}}$ and $I_{\mathrm{Ks}}$ with quinidine in human (as no data in zebrafish were available) (Crumb et al., 2016), presented in Table 1. Each drug was simulated using a pore block model, expressed as:

(4) $$F_{B}=\frac{1}{1+{\left(\frac{\left[C\right]}{IC_{50}}\right)}^{n}}\;,$$

where $F_{B}$ is the fraction of channel block, [C] is the concentration of the drug, $IC_{50}$ is the half‐maximal inhibitory concentration and n is the Hill coefficient. Simulated AP at baseline and after 15 min of 10 µM drug application was compared with the reported experimental measurements.

**Table 1:** Half‐maximal inhibitor concentration ($IC_{50}$) and Hill coefficient (n) for the three ion channel blockers considered in the current study

Compound	Current	$IC_{50}$ (µM)	n
Chromanol 293B	$I_{\mathrm{Ks}}$	2.265	1.49
E4031	$I_{\mathrm{Kr}}$	2.811	1.09
Quinidine	$I_{\mathrm{Na}}$	18.815	1.00
	$I_{\mathrm{Ks}}$	4.899	1.40
	$I_{\mathrm{Kr}}$	0.343	1.00

### Model ionic currents

This section describes the sarcolemmal ion currents, exchangers and pumps, and intracellular ion dynamics of the model, and the experimental data used for their biophysical parameterization. For most of the currents, the same formulation proposed in the original TP04 human ventricular AP model was utilized, with the steady‐state and time‐constant curves of the different gating variables reparametrized by fitting to experimental voltage‐clamp data reported in the literature or measured in the current study. A detailed listing of all relevant equations can be found in Appendix 1.

#### Fast Na^+^ current – $I_{\mathrm{Na}}$

Voltage‐gated fast Na^+^ channels play a crucial role in the rapid upstroke of the ventricular CM AP (*Phase 0*) and are partly responsible for determining the velocity of impulse transmission between cells (Vornanen & Hassinen, 2016). $I_{\mathrm{Na}}$ has been shown to be present in zebrafish ventricular CMs, but at a density lower than in human, leading to a slower AP upstroke (Vornanen & Hassinen, 2016).

The three gates formulation for the Na^+^ current from the TP04 model, which was first introduced by Beeler & Reuter (1977), was adopted:

(5) $$I_{\mathrm{Na}}=G_{\mathrm{Na}}\cdot m^{3}\cdot h\cdot j\cdot \left(V_{m}-E_{\mathrm{Na}}\right),$$

where $G_{\mathrm{Na}}$ is the maximum channel conductance, m is the $V_{m}$‐dependent activation gate, h and j are the fast and slow $V_{m}$‐dependent inactivation gates, and $E_{\mathrm{Na}}$ is the reversal potential for Na^+^. For each of the gating variables, the first‐order Hodgkin–Huxley‐type differential equation, characterized by a $V_{m}$‐dependent steady‐state value and a $V_{m}$‐dependent time constant for reaching the steady‐state value was assumed (ten Tusscher et al., 2004) (see Appendix 1s for detail of the differential equations).

**Figure 2:**
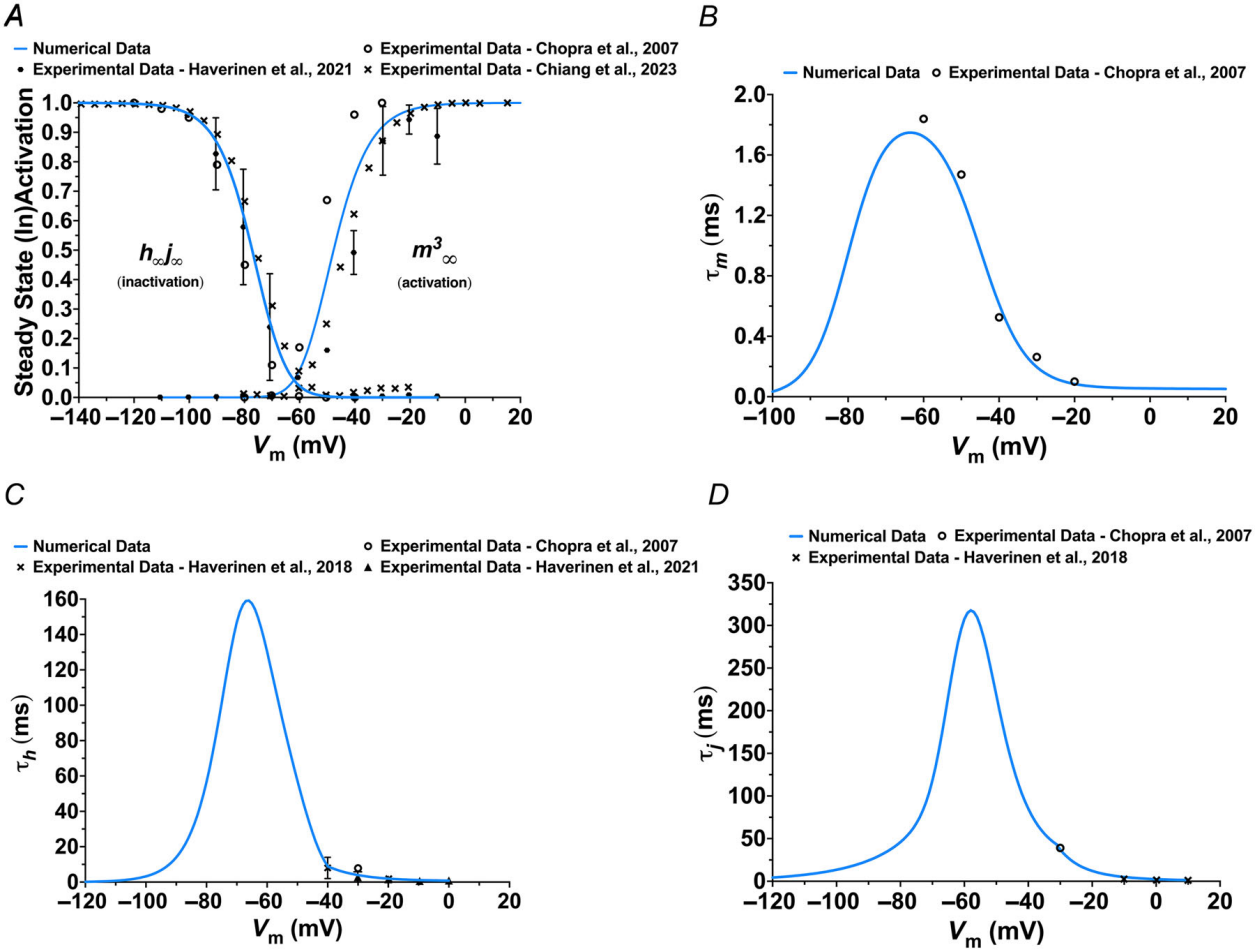
Steady‐state (in)activation and time constant curves describing the voltage ($V_{m}$)‐dependent gating of $I_{\mathrm{Na}}$ Model curves are shown together with experimental data (Chiang et al., 2024; Chopra et al., 2007; Haverinen, Hassinen, Korajoki, et al., 2018; Haverinen et al., 2021), presented as mean or mean ± SD. *A*, steady‐state activation and inactivation curves ($h_{\infty }$, fast $V_{m}$‐dependent inactivation gate; $j_{\infty }$, slow $V_{m}$‐dependent inactivation gate; $m_{\infty }$, $V_{m}$‐dependent activation gate). *B*, activation time constants ($\tau_{m}$). *C*, fast inactivation time constants ($\tau_{h}$). *D*, slow inactivation time constants ($\tau_{j}$).

**Figure 3:**
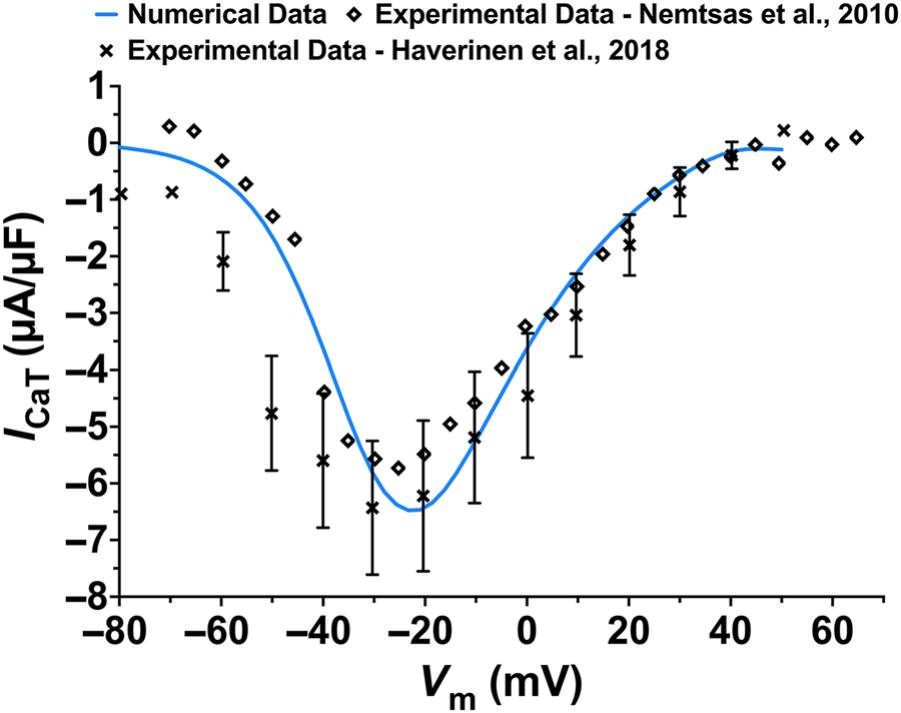
Experimetal current‐voltage (*I*‐*V*) data for *I*_CaT_ Experimental current–voltage (*I–V*) data from the literature (Haverinen, Hassinen, Dash, et al., 2018; Nemtsas et al., 2010) for $I_{\mathrm{CaT}}$, presented as mean or mean ± SD (if available), together with the corresponding computational *I–V* curve.

The steady‐state curve of the activation gates $m_{\infty }^{3}$ was fit (Fig. 2 *A*) with a Boltzmann function using steady‐state data from zebrafish activation gates expressed in Chinese hamster ovary (CHO) cells, as reported by Chopra et al. (2007) (found in fig. 6 and table 6 of the published paper), along with data from Haverinen et al. (2021) (found in fig. 3 of the paper) and Chiang et al. (2024) (found in fig. 3 of their paper). Similarly, the steady‐state curves for the inactivation gates $h_{\infty }j_{\infty }$ were fit (Fig. 2 *A*) using experimental data from published work (Chiang et al., 2024; Chopra et al., 2007; Haverinen et al., 2021).

Time‐to‐peak data from Chopra et al. (2007) (open circles in Fig. 2 *B*) were used to inform the derivation of the activation time constant ($\tau_{m}$) in our model. Specifically, we reparameterized the formulation for $\tau_{m}$ from the TP04 model to fit these experimental time‐to‐peak data (continuous line in Fig. 2 *B*), ensuring the observed kinetics of the fast Na^+^ current accurately reflect the observed values. For $\tau_{h}$ and $\tau_{j}$, experimental recovery from inactivation data was used (Chopra et al., 2007; Haverinen, Hassinen, Korajoki, et al., 2018; Haverinen et al., 2021). The experimental data from Chopra et al. (2007), were fit with two exponentials, generating two distinct time constants. Additionally, scaling was applied as the experimental data were collected at 21–23°C (Chopra et al., 2007) rather than at 28°C. The curves were scaled with a factor of 0.758, corresponding to a *Q*_10_ = 1.74, as reported by Haverinen, Hassinen, Korajoki, et al. (2018). Data from Haverinen, Hassinen, Korajoki, et al. (2018) (found in table 2 of their paper) and Haverinen et al. (2021) (found in figs 2 and 3 of their paper) were also used to fit $\tau_{h}$ and $\tau_{j}$. Since only ‘tail’ data (corresponding to $V_{m}$ between −40 and 0 mV) were available, $\tau_{h}$ and $\tau_{j}$ were derived by shifting and scaling the TP04 equations to the data (Fig. 2 *C* and *D*). In particular, $\tau_{h}$ was multiplied by a factor of 3.5, while $\tau_{j}$ was shifted by 9 mV to the right and scaled by a factor of 0.8.

**Table 2:** **Comparison of action potential (AP) characteristics between the 0D and 1D computational models and experimental recordings for steady‐state pacing with a cycle length of 500 ms. Experimental data are presented as mean ± SD. Isolated hearts from six experimental animals were used, with intracellular AP recordings from three ventricular cells in each (*N* = 6, *n* = 18). APA, AP amplitude; APD, AP duration;** ${dV/dt|}_{\max}$ **, AP upstroke speed; RMP, resting membrane potential;** $V_{\max}$ **, maximum membrane potential**

AP characteristic	Model 0D	Model 1D	Experiment
RMP [mV]	−81	−80	−76 ± 6
$dV/dt_{\max}$ [V/s]	46	26	21 ± 3
$V_{\max}$ [mV]	30	25	27 ± 7
APA [mV]	111	105	105 ± 4
APD_20_ [ms]	65	75	75 ± 13
APD_50_ [ms]	134	136	138 ± 11
APD_80_ [ms]	163	164	169 ± 14
APD_90_ [ms]	173	174	179 ± 17
Triangulation [ms]	40	38	41 ± 8

#### T‐type Ca^2+^ current – $I_{\mathrm{CaT}}$

One of the principal differences between the zebrafish and human ventricular AP is the importance of $I_{\mathrm{CaT}}$ in the zebrafish, specifically during the upstroke of the AP (*Phase 0*) (Vornanen & Hassinen, 2016). In the adult human heart, $I_{\mathrm{CaT}}$ is limited to the sinoatrial node, atrioventricular node and conductive pathways, so is not present in the TP04 model. The formulation for $I_{\mathrm{CaT}}$ used in the current model is based on a previous formulation for the rabbit sinoatrial node (Demir et al., 1994; Zhang et al., 2000):

(6) $$I_{\mathrm{CaT}}=G_{\mathrm{CaT}}\;\cdot d_{T}\cdot f_{T}\cdot \left(V_{m}-E_{\mathrm{Ca}}\right),$$

where $G_{\mathrm{CaT}}$ is the maximum channel conductance, $d_{T}$ is a $V_{m}$‐dependent activation gate, $f_{T}$ is a $V_{m}$‐dependent inactivation gate, and $E_{\mathrm{Ca}}$ is the Ca^2+^ equilibrium potential (see Appendix 1).

**Figure 4:**
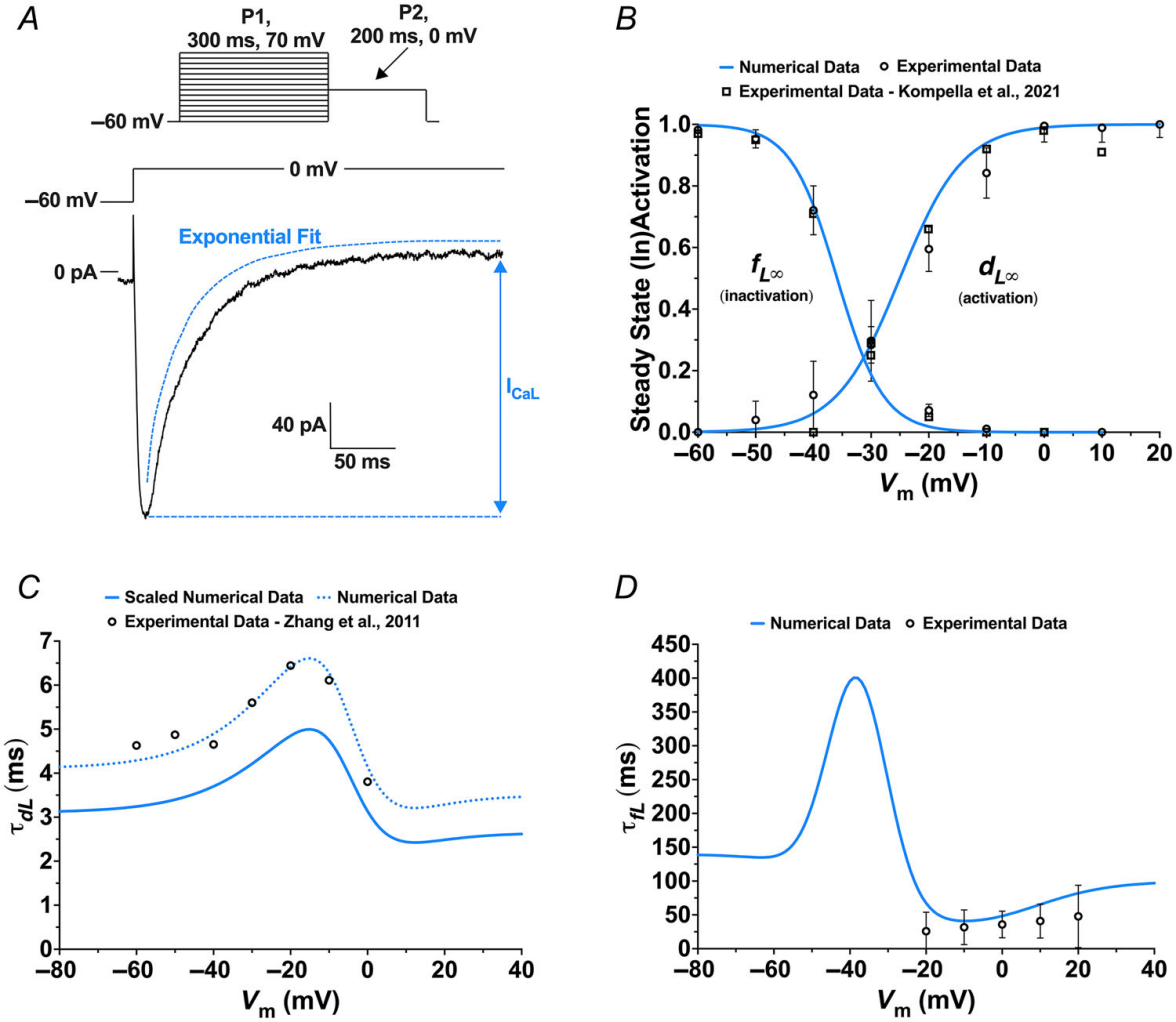
Steady‐state and time constant curves describing the voltage ($V_{m}$)‐dependent gating of $I_{\mathrm{CaL}}$ Model curves are shown together with our newly generated experimental data and data from the literature (Kompella et al., 2021; Zhang et al., 2011), presented as mean or mean ± SD (if available). *A*, voltage clamp protocol (top panel) and typical $I_{\mathrm{CaL}}$ activated by a depolarizing step to 0 mV (bottom panel) in an isolated zebrafish cell of 22.4 pF. In blue lines and fonts are the $I_{\mathrm{CaL}}$ parameters analysed. *B*, steady‐state activation and inactivation curves ($f_{L\infty }$, $V_{m}$‐dependent inactivation gate; $d_{L\infty }$, $V_{m}$‐dependent activation gate). *C*, activation time constants ($\tau_{\mathrm{dL}}$). The dotted line represents the computational fit, and the continuous line represents the scaled curve used in the model. *D*, inactivation time constants ($\tau_{\mathrm{fL}}$).

To fit the experimental behaviour of $I_{\mathrm{CaT}}$ in zebrafish to the rabbit model formulation, data from Haverinen et al. (2021) (found in fig. 3 of their paper) and Nemtsas et al. (2010) (found in fig. 4 of their paper) were used. The experimental patch‐clamp protocol was modelled by incrementing voltage in steps to fit the current–voltage curve (Fig. 3). The formulation was then manually adjusted to ensure that the simulated patch‐clamp protocol produced an I‐V relationship consistent with the experimental data.

$E_{\mathrm{CaT}}$ was obtained using the Nernst equation with the experimental intra‐ and extracellular Ca^2+^ concentrations described in‘Experimental preparation’ above.

#### L‐type Ca^2+^ current – $I_{\mathrm{CaL}}$

$I_{\mathrm{CaL}}$ is responsible for the extended plateau that is characteristic of both the human and zebrafish AP (*Phase 2*) and plays a critical role in initiating contraction in ventricular CMs (Vornanen & Hassinen, 2016). In the zebrafish, $I_{\mathrm{CaL}}$ exists in ventricular CMs, with a current density about fivefold larger than in humans (Hove‐Madsen et al., 2006; Zhang et al., 2011).

The formulation for $I_{\mathrm{CaL}}$ from the TP04 model was adopted:

(7) $$\begin{array}{ccc} I_{\mathrm{CaL}} & = & G_{\mathrm{CaL}}\;\cdot d_{L}\cdot f_{L}\cdot f_{\mathrm{Ca}}\cdot 4\cdot \frac{V_{m}F^{2}}{RT}\\ & & \cdot \frac{{\left[Ca^{2+}\right]}_{i}\;e^{\frac{2V_{m}F}{RT}}-0.341{\left[Ca^{2+}\right]}_{o}}{e^{\frac{2V_{m}F}{RT}}-1}, \end{array}$$

where $G_{\mathrm{CaL}}$ is the maximum channel conductance, $d_{L}$ is a $V_{m}$‐dependent activation gate, $f_{L}$ is a $V_{m}$‐dependent inactivation gate, $f_{\mathrm{Ca}}$ is an intracellular Ca^2+^‐dependent inactivation gate, and the driving force is modelled with a Goldmann–Hodgkin–Katz equation (ten Tusscher et al., 2004) with ${\left[Ca^{2+}\right]}_{i}$ and ${\left[Ca^{2+}\right]}_{o}$ the intracellular and extracellular Ca^2+^ concentrations respectively (see Appendix 1).

The original formulation from TP04 for $f_{\mathrm{Ca}}$ was maintained, as data for the zebrafish were not available. By contrast, for $d_{L\infty }$ and $f_{L\infty }$, the original TP04 formulation was reparameterized to fit our newly generated voltage‐clamp data, as well as data from Kompella et al. (2021) (found in fig. 2 of their paper). Figure 4 *A* shows the voltage clamp protocol used to measure *I*_CaL_ (top panels) and a typical current trace in response to a depolarizing step to 0 mV (bottom panel). Figure 4 *B* shows the steady‐state activation and inactivation curves of the model (continuous line) together with the experimental data.

For the time constant $\tau_{\mathrm{dL}}$ (Fig. 4 *C*) experimental data from Zhang et al. (2011) (found in fig. 3 of their paper) were used. As for the Na^+^ current, the time‐to‐peak was evaluated for each voltage value, and the TP04 formulation was reparameterized to fit the data. However, it was necessary to scale the curve, as the experimental data were collected at 21–23°C (Zhang et al., 2011) rather than at 28°C. The curve was scaled with a factor of 0.756, corresponding to a *Q*_10_ = 1.62, as it produced better restitution behaviour with the model. Finally, the formulation for τ_fL_ was modified from the TP04 model by fitting to our generated patch‐clamp data (Fig. 4 *D*).

#### Rapid delayed rectifier K^+^ current – $I_{\mathrm{Kr}}$

**Figure 5:**
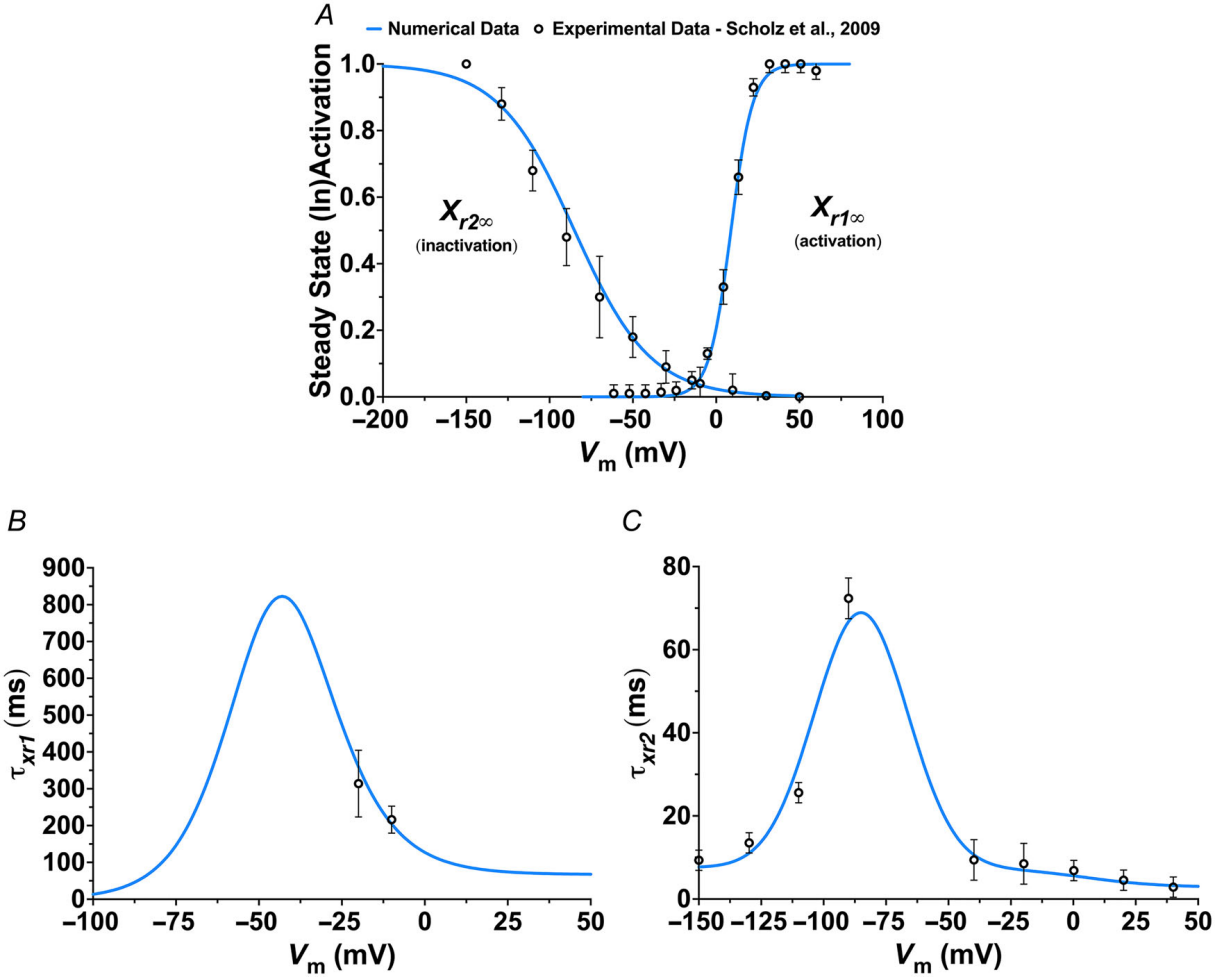
Steady‐state and time constant curves describing the voltage ($V_{m}$)‐dependent gating of $I_{\mathrm{Kr}}$ Model curves are shown together with experimental data (Scholz et al., 2009), presented as mean or mean ± SD. *A*, steady‐state activation and inactivation curves ($X_{r2\infty }$, $V_{m}$‐dependent inactivation gate; $X_{r1\infty }$, $V_{m}$‐dependent activation gate). *B*, activation time constants ($\tau_{\mathrm{xr}1}$). *C*, inactivation time constants ($\tau_{\mathrm{xr}2}$).

In zebrafish, $I_{\mathrm{Kr}}$ is known to play a significant role in the AP plateau (*Phase 2*) and repolarization (*Phase 3*) (Hassinen, Haverinen, & Vornanen, 2015; Nemtsas et al., 2010). A slightly different formulation than that of the original TP04 model has been chosen to better fit the experimental patch clamp data from Scholz et al. (2009) (found in fig. 5 of their paper):

(9) $$I_{\mathrm{Kr}}=G_{\mathrm{Kr}}\;\cdot \sqrt{\frac{{\left[K^{+}\right]}_{o}}{4.85}}\cdot x_{r1}\cdot x_{r2}\cdot \left(V_{m}-E_{K}\right),$$

where $G_{\mathrm{Kr}}$ is the maximum conductance of the channel, $\sqrt{\frac{{\left[K^{+}\right]}_{o}}{4.85}}$ represents the extracellular K^+^ concentration dependence of the current, $x_{r1}$ is the activation gate, $x_{r2}$ the inactivation gate, and $E_{k}$ the K^+^ reversal potential.

Starting from experimental data from Scholz et al. (2009), the steady‐state formulation of the TP04 model for activation and inactivation gates was reparameterized to fit the experiments (Fig. 5 *A*). Contrary to the steady‐state value of the activation gate, data associated with activation and the time constant are only reported for −10 and −20 mV (Scholz et al., 2009). Hence, the original formulation for the activation time constant from the TP04 model was scaled to fit the available experimental data (Fig. 5 *B*). The inactivation gate time constant, $\tau_{\mathrm{xr}2}$, was also fit to the available experimental data (Fig. 5 *C*).

#### Slow delayed rectifier K^+^ current – $I_{\mathrm{Ks}}$

The first evidence of the existence of $I_{\mathrm{Ks}}$ in zebrafish, which is involved in the AP plateau (*Phase 2*) and repolarization (*Phase 3*) in mammals, was provided by Abramochkin et al. (2018), along with a characterization of its electrophysiological properties. The formulation for the $I_{\mathrm{Ks}}$ current in the model has been slightly modified from the original TP04 formulation by altering the activation kinetics from quadratic ($x_{s}^{2}$) to linear ($x_{s}$), to better fit the experimental data:

(8) $$I_{\mathrm{Ks}}=G_{\mathrm{Ks}}\;\cdot x_{s}\cdot \left(V_{m}-E_{\mathrm{ks}}\right),$$

where $G_{\mathrm{Ks}}$ is the maximum conductance of the channel, $x_{S}$ is the activation gate, and $E_{\mathrm{Ks}}$ is a reversal potential determined by assuming a large permeability to K^+^ and a small permeability to Na^+^, as in the original TP04 model (since no experimental data for the zebrafish are available; see Appendix 1).

**Figure 6:**
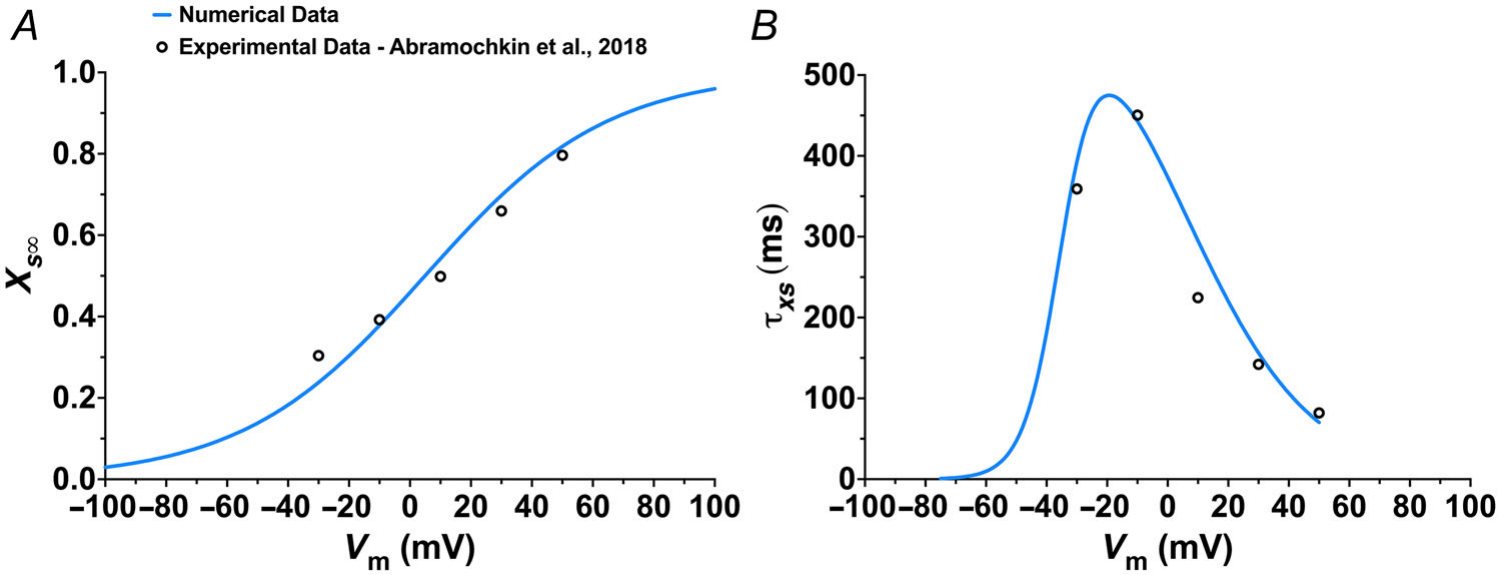
Steady‐state and time constant curves describing the voltage ($V_{m}$)‐dependent gating of $I_{\mathrm{Ks}}$ Model curves are shown together with experimental data (Abramochkin et al., 2018). *A*, steady‐state activation ($X_{s\infty }$). *B*, activation time constant ($\tau_{\mathrm{xs}}$).

Steady‐state activation, $x_{S\infty }$, and values of the activation gate time constant $\tau_{\mathrm{xS}}$ were directly estimated from current recordings presented in t Abramochkin et al. (2018) (found in fig. 3 of their paper). Figure 6 shows the data and fits used for the model (see Appendix 1 for the detailed formulation).

#### Inward rectifier K^+^ current – $I_{K1}$

The inward rectifier K^+^ current influences AP morphology during initial depolarization and final repolarization. More importantly, $I_{K1}$ is critical for stabilizing RMP during the diastolic period (*Phase 4*) (Hassinen, Haverinen, et al., 2015). As for the other K^+^ currents, modifications were made to the original formulation to properly fit the available experimental data. The current is defined in the TP04 model as:

(10) $$I_{K1}=G_{K1}\;\cdot \sqrt{\frac{{\left[K^{+}\right]}_{o}}{4.85}}\cdot x_{K1\infty }\cdot \left(V_{m}-E_{K}\right),$$

where $G_{K1}$ is the maximum channel conductance, $\sqrt{\frac{{\left[K^{+}\right]}_{o}}{4.85}}$ represents the extracellular K^+^ dependence of the current and $x_{K1\infty }$ is a time‐independent inward rectifying factor that is a function of the potential (see Appendix 1).

**Figure 7:**
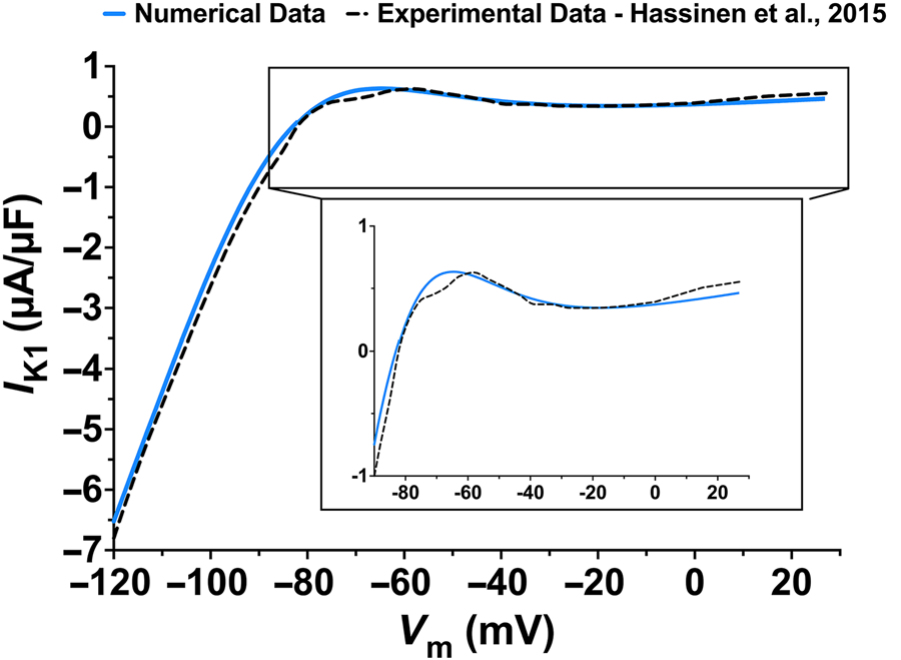
Experimental current‐voltage (*I‐V*) data for *I*_K1_ Experimental current–voltage (*I–V*) data from Hassinen, Haverinen, et al. (2015) and the corresponding computational *I*–*V* curve for $I_{K1}$

The equation for $x_{K1\infty }$ was derived from the *I–V* curve for the zebrafish reported in Hassinen, Haverinen, et al. (2015) (found in fig. 3 of their paper). Figure 7 shows the experimental and computational *I–V* curves.

#### Exchangers, pumps and background currents

Concerning the currents associated with the Na^+^–Ca^2+^ exchanger, $I_{\mathrm{NaCa}}$, and the Na^+^–K^+^ pump, $I_{\mathrm{NaK}}$, as well as the sarcolemmal Ca^2+^ pump, $I_{\mathrm{pCa}},$ and the Ca^2+^ and Na^+^ background currents, $I_{\mathrm{bCa}}$ and $I_{\mathrm{bNa}}$, the formulations present in the TP04 model were adopted, as no experimental data for the zebrafish are currently available.

(11) $$I_{\mathrm{NaCa}}=k_{\mathrm{NaCa}}\;\cdot \frac{e^{\gamma V_{m}F/RT}{\left[Na^{+}\right]}_{i}^{3}{\left[Ca^{2+}\right]}_{o}-2.5e^{\left(\gamma -1\right)V_{m}F/RT}{\left[Ca^{2+}\right]}_{o}^{3}{\left[Ca^{2+}\right]}_{i}}{\left(K_{\mathrm{mNai}}^{3}+{\left[Na^{+}\right]}_{o}^{3}\right)\left(K_{\mathrm{mCa}}+{\left[Ca^{2+}\right]}_{o}\right)\left(1+k_{\mathrm{sst}}e^{\left(\gamma -1\right)V_{m}F/RT}\right)},$$

(12) $$I_{\mathrm{NaK}}=P_{\mathrm{NaK}}\;\cdot \frac{{\left[K^{+}\right]}_{o}{\left[Na^{+}\right]}_{i}}{\left({\left[K^{+}\right]}_{o}+K_{\mathrm{mK}}\right)\left({\left[Na^{+}\right]}_{i}+K_{\mathrm{mNa}}\right)\left(1+0.1245e^{-\frac{0.1V_{m}F}{RT}}+0.0353e^{-V_{m}F/RT}\right)},$$

(13) $$I_{\mathrm{pCa}}=G_{\mathrm{pCa}}\;\cdot \frac{{\left[Ca^{2+}\right]}_{i}}{K_{\mathrm{pCa}}+{\left[Ca^{2+}\right]}_{i}},$$

(14) $$I_{\mathrm{bNa}}=G_{\mathrm{bNa}}\;\cdot \left(V_{m}-E_{\mathrm{Na}}\right),$$

(15) $$I_{\mathrm{bCa}}=G_{\mathrm{bCa}}\;\cdot \left(V_{m}-E_{\mathrm{Ca}}\right).$$

#### Intracellular ion dynamics

The dynamics of intracellular Na^+^ concentration was formulated as in the TP04 model:

(16) $$\frac{dNa_{i}}{dt}=-\frac{I_{\mathrm{Na}}+I_{\mathrm{bNa}}+3I_{\mathrm{NaK}}+3I_{\mathrm{NaCa}}}{V_{c}F}C_{m}A_{c}.$$

Due to the removal of $I_{\mathrm{to}}$ and $I_{\mathrm{pK}}$ from the model, the dynamics of intracellular K^+^ concentration were expressed as:

(17) $$\frac{dK_{i}}{dt}=-\frac{I_{K1}+I_{\mathrm{Kr}}+I_{\mathrm{Ks}}-2I_{\mathrm{NaK}}+I_{\mathrm{stim}}}{V_{c}F}C_{m}A_{c}.$$

Finally, due to the addition of $I_{\mathrm{CaT}}$, intracellular Ca^2+^ dynamics were modified as follows:

(18) $$I_{\mathrm{leak}}=V_{\mathrm{leak}}\;\cdot \left({\left[Ca^{2+}\right]}_{\mathrm{sr}}-{\left[Ca^{2+}\right]}_{i}\right),$$

(19) $$I_{\mathrm{up}}=\frac{V_{\mathrm{maxup}}}{1+K_{\mathrm{up}}^{2}/{\left[Ca^{2+}\right]}_{i}^{2}}\;,$$

(20) $$I_{\mathrm{rel}}=\left(a_{\mathrm{rel}}\cdot \frac{{\left[Ca^{2+}\right]}_{\mathrm{sr}}^{2}}{b_{\mathrm{rel}}^{2}+{\left[Ca^{2+}\right]}_{\mathrm{sr}}^{2}}+c_{\mathrm{rel}}\right)\;\cdot d_{L}\cdot g,$$

(21) $$Ca_{\mathrm{ibufc}}=\frac{{\left[Ca^{2+}\right]}_{i}\cdot buf_{c}}{{\left[Ca^{2+}\right]}_{i}+kbuf_{c}}\;,$$

(22) $$\begin{array}{ccc} \frac{dCa_{\mathrm{itotal}}}{dt} & = & -\frac{I_{\mathrm{CaL}}+I_{\mathrm{CaT}}+I_{\mathrm{bCa}}+I_{\mathrm{pCa}}-2I_{\mathrm{NaCa}}}{2V_{c}F}\\ & & C_{m}A_{c}+I_{\mathrm{leak}}-I_{\mathrm{up}}+I_{\mathrm{rel}}, \end{array}$$

(23) $$Ca_{\mathrm{srbufsr}}=\frac{{\left[Ca^{2+}\right]}_{\mathrm{sr}}\cdot buf_{\mathrm{sr}}}{{\left[Ca^{2+}\right]}_{\mathrm{sr}}+kbuf_{\mathrm{sr}}}\;,$$

(24) $$\frac{dCa_{\mathrm{srtotal}}}{dt}=\frac{V_{C}}{V_{\mathrm{SR}}}\;\cdot \left(I_{\mathrm{up}}-I_{\mathrm{leak}}-I_{\mathrm{rel}}\right),$$

where $I_{\mathrm{leak}}$ is a leakage current from the SR to the cytoplasm, $I_{\mathrm{up}}$ is the sarco‐endoplasmic reticulum ATP‐ase (SERCA) pump‐mediated flux of Ca^2+^ into the SR, $I_{\mathrm{rel}}$ is the Ca^2+^‐induced Ca^2+^‐release current from the SR, $d_{L}$ is the activation gate of $I_{\mathrm{CaL}}$ reused as the activation gate of $I_{\mathrm{rel}}$, and g is the Ca^2+^‐dependent inactivation gate of $I_{\mathrm{rel}}$ (see Appendix 1 for details). $Ca_{\mathrm{iTotal}}$ is the total Ca^2+^ in the cytoplasm, which consists of the free (${\left[Ca^{2+}\right]}_{i}$) and buffered ($Ca_{\mathrm{ibufc}}$) Ca^2+^ in the cytoplasm. $Ca_{\mathrm{srtotal}}$ is the total Ca^2+^ in the SR, which consists of the free (${\left[Ca^{2+}\right]}_{\mathrm{SR}}$) and buffered ($Ca_{\mathrm{srbufsr}}$) Ca^2+^ in the SR (ten Tusscher et al., 2004).

Finally, regarding the volumes of the various intracellular compartments, the cytoplasmic volume $V_{c}$ is set to 390 µm^3^, following reported dimensions of the zebrafish ventricular CM (Brette et al., 2008) and assuming that $V_{c}$ corresponds to 40% of the cell volume. The volume of the sarcoplasmic reticulum $V_{\mathrm{SR}}$ is calculated as 5% of the cytoplasmic volume, in accordance with data from other fish species (Shiels & Galli, 2014). The capacitive area, $A_{c}$, is assumed to be twice the surface area of the cell, as in O'Hara et al. (2011), with the surface area of the cell set to 1500 µm^2^, corresponding to the lower bound value reported for the zebrafish (Brette et al., 2008). This leads to a cell capacitance of 30 pF, which is within the experimentally measured range (27.2 ± 8.8 pF).

## Results

### Steady‐state pacing protocol

**Figure 8:**
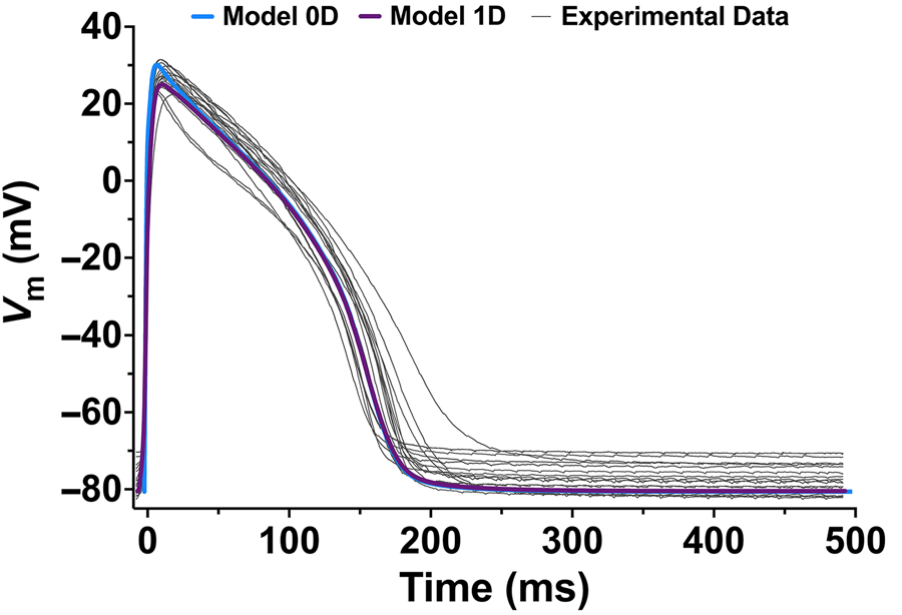
Comparison of experimental action potential data to computational models Comparison of the experimental action potential recordings and 0D and 1D computational outputs for steady‐state pacing with a cycle length of 500 ms

Figure 8 shows the comparison between the experimental AP recordings and the output of the 0D and 1D computational models during steady‐state pacing at 2 Hz. Both the 0D and 1D computational APs fall within the range of the experimental data, showing good agreement with the recorded AP morphology, including a lack of a spike–notch–dome architecture due to the absence of $I_{\mathrm{to}}$ in the zebrafish.

A quantitative comparison of measured AP characteristics for the experimental and modelled signals is given in Table 2. The results show that all features are within the range of experimental values (mean ± SD), except for APA and ${dV/dt|}_{\max}$, which are greater for the 0D model due to the absence of electrotonic coupling that exists in tissue (from which the recordings were made) and included in the 1D model as an axial current.

**Figure 9:**
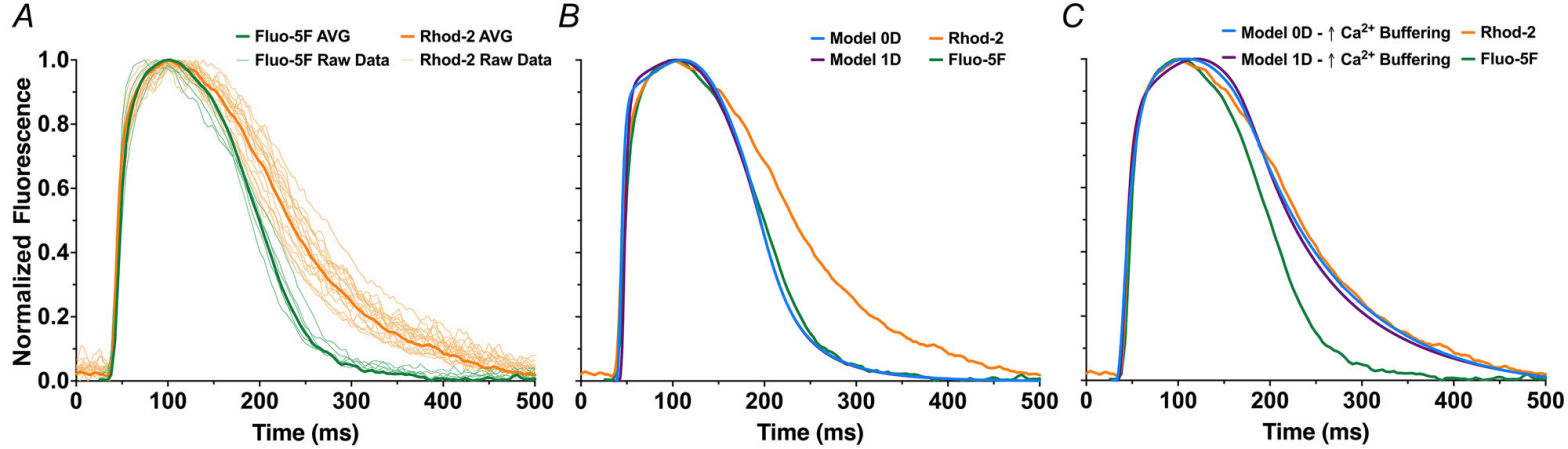
Comparison of the experimental calcium transient data to computational models Comparison of experimental calcium transient recordings and 0D and 1D computational outputs for steady‐state pacing with a cycle length of 500 ms. A, individual and average experimental calcium transient recordings using Rhod‐2 (higher affinity dye) and Fluo‐5F (lower affinity calcium dye). *B*, average experimental calcium transient signals from *A* compared to the outputs from the 0D and 1D computational simulations. *C*, calcium transients from 0D and 1D computational simulations mimicking increased Ca^2+^ buffering with Rhod‐2 compared to average experimental calcium transients from *A*.

Figure 9 shows the comparison between the experimental CaT recordings using the higher (Rhod‐2) and lower (Fluo‐5F) affinity Ca^2+^ dyes and the output for the 0D and 1D computational models during steady‐state pacing with a cycle length of 500 ms. In Fig. 9 *A*, it is evident that CaT recorded with Rhod‐2 are longer than those obtained with Fluo‐5F, showing an artefactual difference due to the relatively high Ca^2+^ affinity, which has been shown previously in mammals (note that while useful for investigating only Ca^2+^ dynamics, CaT amplitude cannot be compared with non‐ratiometric dyes, as total fluorescence depends on dye brightness, loading and light exposure, and thus CaT values are scaled between 0 and 1) (Babcock et al., 1997; Davidson & Duchen, 2018; Pozzan & Rudolf, 2008; Stamm et al., 2003). Thus, as shown in Fig. 9 *B*, both the 0D and 1D computational CaT show good agreement with the Fluo‐5F recorded CaT morphology. In fact, as seen in Fig. 9 *C*, when Ca^2+^ buffering parameters were modified in the models to mimic the higher Ca^2+^ buffering ($buf_{c}$ and $\tau_{g}$ increased 1.5×, $kbuf_{\mathrm{sr}}$ increased 4×, and ${\left[Ca^{2+}\right]}_{i}$ increased by 0.4×), the 0D and 1D computational CaT instead agreed with the Rhod‐2 recorded CaT.

A quantitative comparison of measured CaT characteristics for the experimental and modelled signals is given in Table 3. The results show that all features are within the range of experimental values calculated from the Fluo‐5F signals (mean ± SD) but not for the values calculated from the Rhod‐2 signals, due to the higher calcium affinity of the dye. Also, while both are within the experimental range, it is worth noting that CaT upstroke speed (${dF/dt|}_{\max}$) is greater for the 0D than the 1D model due to the lack of electrotonic coupling (similar to the difference in AP upstroke speed).

**Table 3:** **Comparison of calcium transient (CaT) characteristics between the 0D and 1D computational models and experimental recordings for steady‐state pacing with a cycle length of 500 ms. Experimental data are presented as mean ± SD. Isolated hearts from six experimental animals were used, with signals averaged over regions of interest taken from three distinct regions of the ventricle in each (*N* = 6, *n* = 18). CaTD, CaT duration;** ${dF/dt|}_{\max}$ **, CaT upstroke speed**

CaT characteristic	Model 0D	Model 1D	Experiment (Rhod‐2)	Experiment (Fluo‐5F)
${dF/dt|}_{\max}$ [ms^−1^]	0.057	0.052	0.028 ± 0.005	0.055 ± 0.006
CaTD_20_ [ms]	117	115	137 ± 15	115 ± 9
CaTD_50_ [ms]	149	149	192 ± 16	152 ± 12
CaTD_80_ [ms]	185	185	276 ± 19	196 ± 14

**Figure 10:**
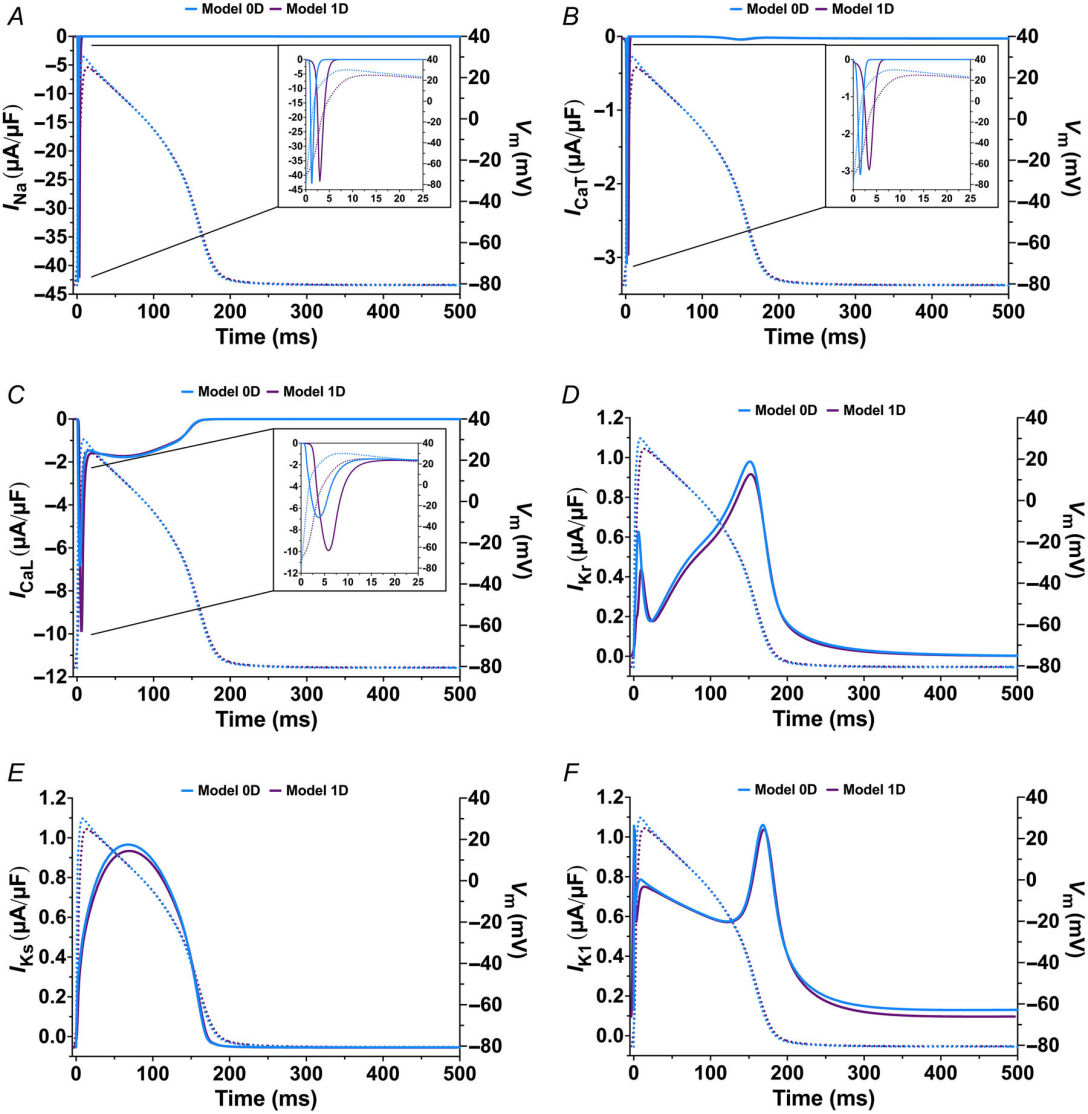
Comparison of the principal currents in the 0D and 1D computational models during steady‐state pacing with a cycle length of 500 ms, including the associated action potentials (dotted lines) *A*, fast sodium ($I_{\mathrm{Na}}$); *B*, T‐type calcium ($I_{\mathrm{CaT}}$); *C*, L‐type calcium ($I_{\mathrm{CaL}}$); *D*, rapid delayed rectifier potassium ($I_{\mathrm{Kr}}$); *E*, slow delayed rectifier potassium ($I_{\mathrm{Ks}}$); and *F*, inward rectifier potassium ($I_{K1}$) currents.

Figure 10 shows the principal currents over the course of a single AP in the 0D and 1D computational models for steady‐state pacing with a cycle length of 500 ms. The currents are largely similar for the two models, with small differences in peak current density and dynamics of the depolarizing currents. For the 1D model, the peak density of $I_{\mathrm{CaL}}$ is greater than the 0D model, while the peak density of all other currents is slightly decreased, and the peaks of the depolarizing currents are slightly delayed due to electrotonic coupling in the tissue model.

In Fig. 10 *A*, the contribution of voltage‐gated Na^+^ channels in zebrafish ventricular CMs, which are critical for the rapid upstroke of the AP (*Phase 0*) and influence the speed of impulse transmission across the heart, is evident. The lower peak density of $I_{\mathrm{Na}}$ compared to human and other mammals is largely responsible for the slower AP upstroke speed in zebrafish and may also contribute to the slower speed of impulse conduction (Vornanen & Hassinen, 2016). In Fig. 10 *B*, a contribution of $I_{\mathrm{CaT}}$ to the zebrafish ventricular AP upstroke (*Phase 0*) is evident, which has been shown experimentally (Alday et al., 2014), and is in contrast to mammals, in which $I_{\mathrm{CaT}}$ exists only in the sinoatrial node, atrioventricular node and conduction system (Vornanen & Hassinen, 2016). In Fig. 10 *C*, it is clear that as in mammals, $I_{\mathrm{CaL}}$ plays a critical role in sustaining the AP plateau (*Phase 2*) and supplying Ca^2+^ for contraction to occur (Vornanen & Hassinen, 2016). The current density of $I_{\mathrm{CaL}}$ in the models, however, is higher than for human, with peak and plateau values almost double those found in recent human ventricular AP models (Bartolucci et al., 2020; O'Hara et al., 2011; Tomek et al., 2019), such that it will have a larger direct role in the CaT. Figure 10*D*–*F* show the principal repolarization currents, with $I_{\mathrm{Kr}}$ and $I_{\mathrm{Ks}}$ having similar dynamics to recent human ventricular AP models (O'Hara et al., 2011), while $I_{K1}$ shows remarkably different behaviour, contributing to the plateau of the AP (*Phase 2*), in agreement with reports for zebrafish (Ravens, 2018).

### S1–S2 restitution pacing protocol

**Figure 11:**
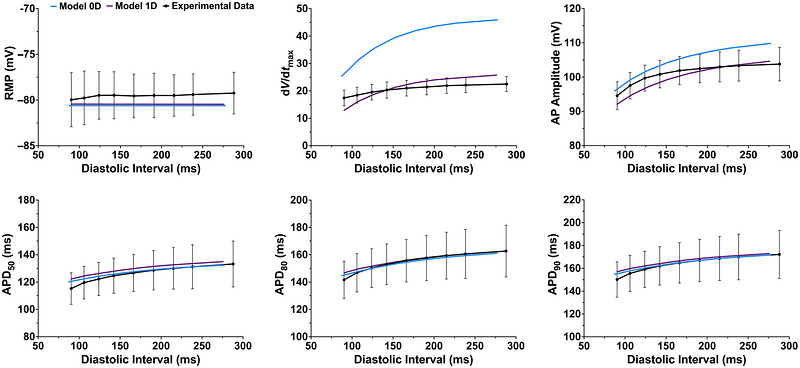
Comparison of experimental and computational restitution behaviour of several action potential (AP) characteristics for the S1–S2 pacing protocol The experimental AP characteristics, including resting membrane potential (RMP), maximum upstroke speed ($dV/dt_{\max}$) and AP duration (APD), were derived from recordings in isolated hearts, and are presented as mean ± SD.

Figure 11 shows the restitution behaviour of various AP characteristics for the 0D and 1D computational models obtained with the S1–S2 pacing protocol, compared to averages of the experimental results (figures showing individual experimental curves are available on Figshare, DOI: https://doi.org/10.6084/m9.figshare.27997817). The restitution behaviour of RMP and APD for both the 0D and 1D computational models aligns well with the experimental recordings and is also in agreement with reports for isolated zebrafish ventricular CMs (Koopman et al., 2021). For ${dV/dt|}_{\max}$ and APA, however, only the output of the 1D model fits the experimental data, as values for the 0D model are greater and outside of the experimental range due to electrotonic effects of cells coupled in tissue.

Unfortunately, due to signal degradation related to photobleaching with the use of the lower affinity Ca^2+^ dye (Fluo‐5F), we were unable to make measurements of CaT with the S1–S2 pacing protocol; CaT restitution dynamics for the dynamic pacing protocol are provided below.

### Dynamic restitution pacing protocol

**Figure 12:**
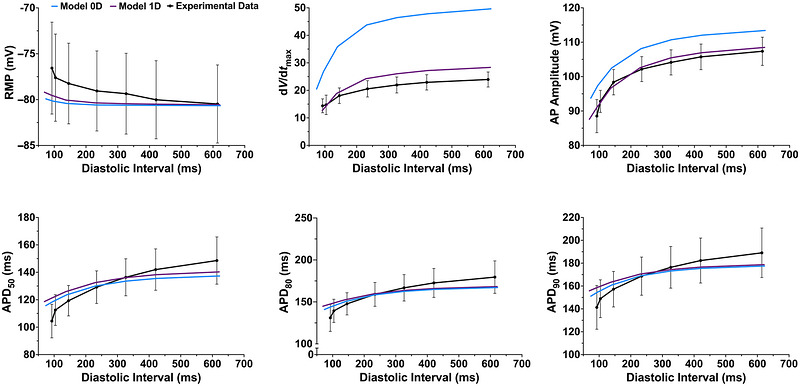
Comparison of experimental and computational restitution behaviour of several action potential (AP) characteristics for the dynamic pacing protocol The experimental AP characteristics, including resting membrane potential (RMP), maximum upstroke speed ($dV/dt_{\max}$) and AP duration (APD), were derived from recordings in isolated hearts, and are presented as mean ± SD.

Figure 12 shows the restitution behaviour of various AP characteristics for the 0D and 1D computational models obtained with the dynamic pacing protocol, compared to the experimental results. As for the S1–S2 protocol, the 0D and 1D models generate RMP and APD restitution behaviour within the experimental range, but the curves are generally flatter in each case, while only ${dV/dt|}_{\max}$ and APA of the 1D model fit the experimental data, with 0D values again being greater and outside of the experimental range.

**Figure 13:**
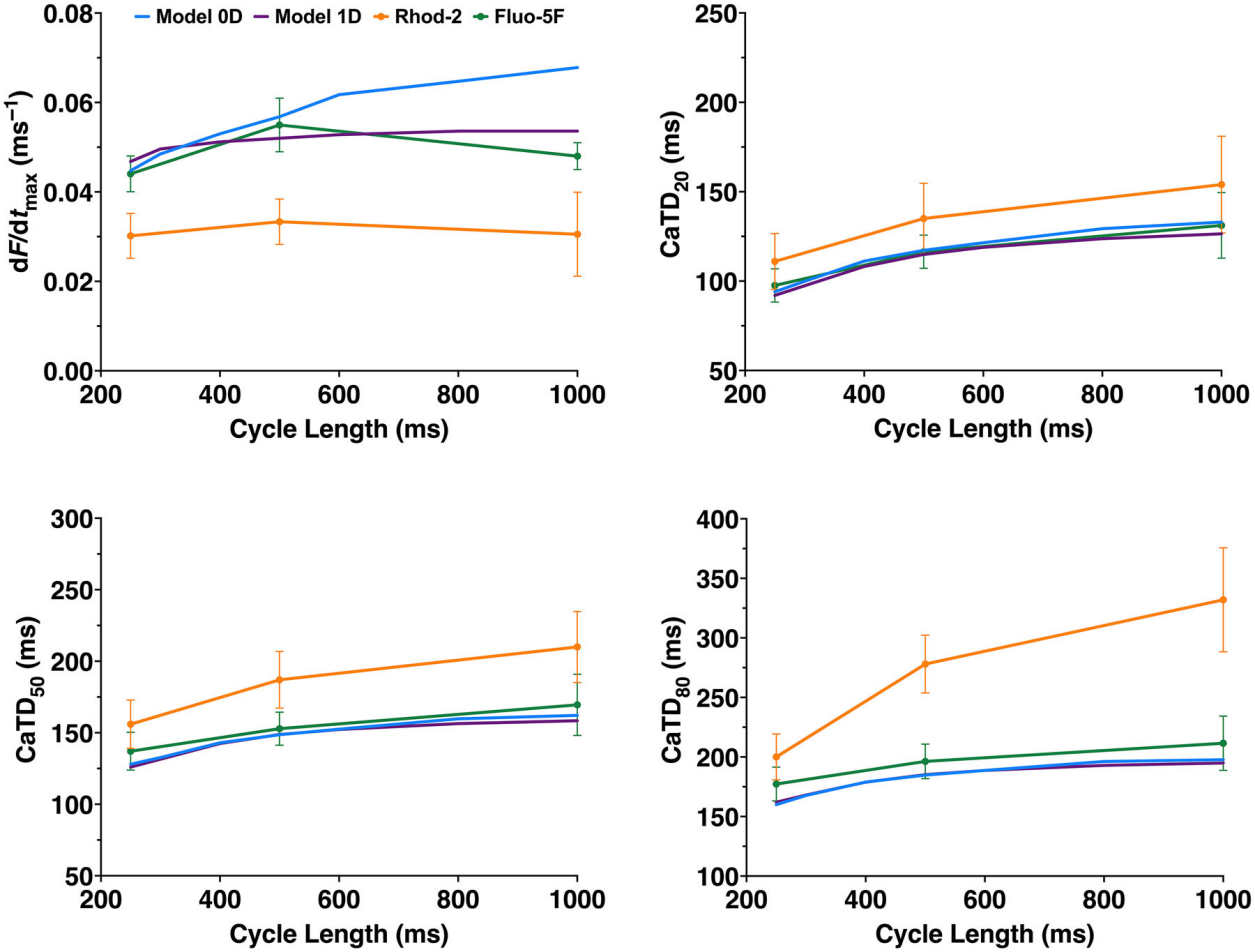
Comparison of the experimental and computational restitution behaviour of several calcium transient (CaT) characteristics for the dynamic pacing protocol The experimental CaT measurements, including maximum upstroke speed ($dF/dt_{\max}$) and CaT durations (CaTD) were obtained using two calcium dyes with different affinities – Rhod‐2 (higher affinity) and Fluo‐5F (lower affinity) – in isolated hearts, and are presented as mean ± SD.

Figure 13 shows the restitution behaviour of ${dF/dt|}_{\max}$ and CaTD for the 0D and 1D computational models obtained with the dynamic pacing protocol, compared to the experimental results obtained using both the higher (Rhod‐2) and lower (Fluo‐5F) Ca^2+^ affinity dyes. The behaviour of both $dF/dt{|}_{\max}$ and CaTD from the models were similar to experiments using the lower affinity dye, except for $dF/dt{|}_{\max}$ at longer cycles lengths, for which the 0D model produced higher values outside of the experimental range. The experimental measurements using the higher affinity dye, on the other hand, differed from the lower affinity dye and computational results, with lower $dF/dt{|}_{\max}$ and greater CaTD values.

### Sensitivity analysis

**Figure 14:**
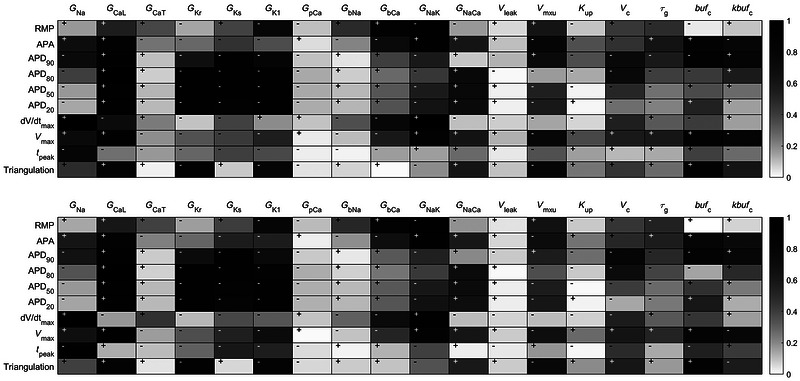
Sensitivity analyses for the principal ionic current conductances and other key parameters determining characteristics of the action potential (AP) in the 0D (top) and 1D (bottom) computational models APA, AP amplitude; APD, AP duration; $dV/dt{|}_{\max}$, AP upstroke speed; RMP, resting membrane potential; $t_{\mathrm{peak}}$, time to maximum membrane potential; $V_{\max}$, maximum membrane potential.

Figure 14 shows the results of the sensitivity analyses, which were similar for the 0D and 1D simulations. They reveal that characteristics of the AP upstroke (*Phase 0*) are determined primarily by fast Na^+^ channels, in agreement with Nemtsas et al. (2010). However, the total block of $I_{\mathrm{Na}}$ current did not prevent excitation, demonstrating a pivotal role of Ca^2+^ influx for ventricular CM excitation in the zebrafish. An impact of the Na^+^–K^+^ pump on $dV/dt{|}_{\max}$ and APA was also evident, which is an aspect not previously reported in the literature for zebrafish or human. Also different from what has been observed in the ten Tusscher et al. human ventricular model using similar techniques to the current study (Romero et al., 2009), the Na^+^–K^+^ pump had a mild influence on AP repolarization.

Regarding Ca^2+^ currents, the sensitivity analysis indicates that $I_{\mathrm{CaT}}$ and $I_{\mathrm{CaL}}$ have distinct influences on ventricular AP morphology. $I_{\mathrm{CaL}}$ appears pivotal for the plateau of the zebrafish AP (*Phase 2*), as in human and mammals, and in agreement with experimental reports (Brette et al., 2008; Nemtsas et al., 2010; Zhang et al., 2011), and also influences APA and $V_{\max}$. In fact, the only parameters that seem to not be greatly affected by $I_{\mathrm{CaL}}$ are those related to *Phase 0* (i.e. AP upstroke speed and time to peak). In contrast, $I_{\mathrm{CaT}}$ primarily influences the speed of the AP upstroke.

The sensitivity analysis also highlights that the three principal K^+^ currents ($I_{\mathrm{Kr}}$, $I_{\mathrm{Ks}}$ and $I_{K1}$) play a similar role in AP, driving repolarization, although AP triangulation is less affected by $I_{\mathrm{Ks}}$. In the model, the $I_{\mathrm{Ks}}$:$I_{K1}$ maximal conductance ratio ($G_{\mathrm{Ks}}$:$G_{K1}$) is 1:5, while the $I_{\mathrm{Ks}}$:$I_{\mathrm{Kr}}$ maximal conductance ratio ($G_{\mathrm{Ks}}$:$G_{\mathrm{Kr}}$) is 1:12, in agreement with previous reports from (Abramochkin et al., 2018; Hassinen, Haverinen, & Vornanen, 2015; Hassinen, Haverinen, et al., 2015). Yet, despite the relatively small maximal conductance of $I_{\mathrm{Ks}}$ compared to $I_{\mathrm{Kr}}$ and $I_{K1}$, the resulting current amplitude of $I_{\mathrm{Ks}}$ during the action potential is similar to that of $I_{\mathrm{Kr}}$ (Fig. 10*D*–*F*). This highlights that $I_{\mathrm{Ks}}$ still has a substantial influence on AP repolarization.

There is also an apparent influence of Ca^2+^ handling on AP repolarization, through cytosolic Ca^2+^ buffering $(buf_{c}$) activity of the Na^+^–Ca^2+^ exchanger.

### Calcium handling in the zebrafish model

**Figure 15:**
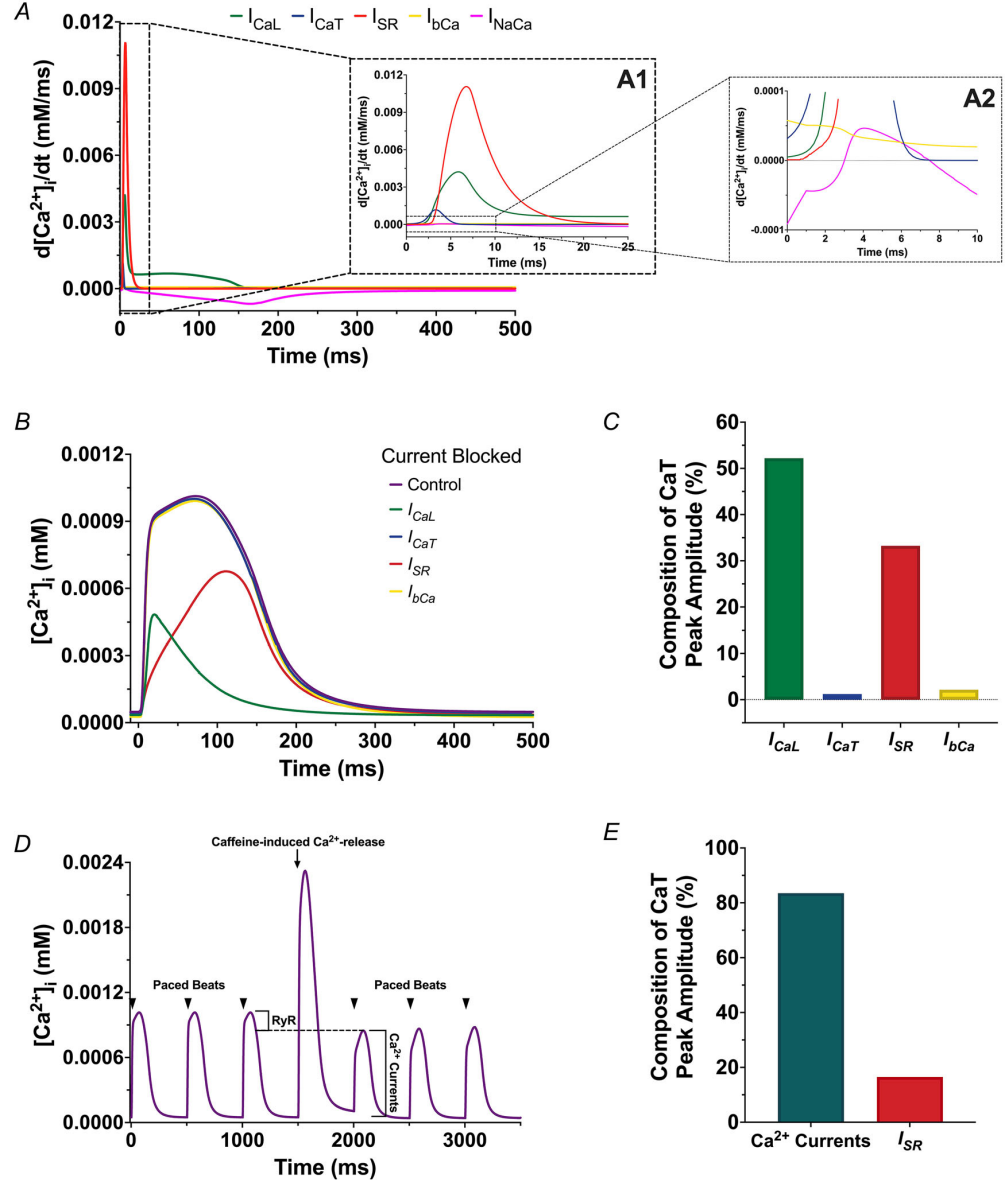
Cytosolic calcium (Ca^2+^) flows contributing to the calcium transient (CaT) in the 1D computational model *A*, the rate of change of intracellular calcium concentration (${\left[Ca^{2+}\right]}_{i}$) due to sarcoplasmic reticulum (SR) Ca^2+^ release ($I_{\mathrm{SR}}$) or Ca^2+^ influx through sarcolemmal Ca^2+^ channels (L‐type, $I_{\mathrm{CaL}}$; T‐type, $I_{\mathrm{CaT}}$; background, $I_{\mathrm{bCa}}$; and the Na^+^–Ca^2+^ exchanger, $I_{\mathrm{NaCa}}$). *B*, effect of individually blocking each flow of Ca^2+^ in the computational model on the CaT. *C*, percentage contribution of each Ca^2+^ flow to peak CaT amplitude. *D*, simulated experiment, mimicking that performed by Bovo et al. (2013). Arrowheads represent stimulated beats. *E*, relative contribution of sarcolemmal and SR Ca^2+^ flow to the CaT.

As Ca^2+^ handling differs in zebrafish ventricular CMs compared to human and other mammals (van Opbergen et al., 2018), we sought to further explore cytosolic flows of Ca^2+^ contributing to the CaT in our computational model. Specifically, in Fig. 15 we compared the relative contribution of ryanodine receptor‐mediated SR Ca^2+^ release and Ca^2+^ influx through sarcolemmal Ca^2+^ channels to the change of ${\left[Ca^{2+}\right]}_{i}$. Figure 15 *A* presents the rate of change of ${\left[Ca^{2+}\right]}_{i}$ due to each current (see eqn 68 in Appendix 1), highlighting its magnitude and time course during the CaT. From this (and inset panel *A*1) we can see that initially (for the first ∼20 ms of the CaT), the principal contributors to the change of cytosolic Ca^2+^ flow from the SR – either via Ca^2+^‐induced Ca^2+^‐release ($I_{\mathrm{rel}}$) or ryanodine receptor leak ($I_{\mathrm{rel}}$, which we have combined as $I_{\mathrm{SR}}$) – or $I_{\mathrm{CaL}}$, with an additional small contribution from $I_{\mathrm{CaT}}$, reverse mode of the Na^+^–Ca^2+^ exchanger, and $I_{\mathrm{bCa}}$ (inset panel *A*2). After that, Ca^2+^ flow from the SR ceases, and the remainder of the CaT is due primarily to a persistent level of $I_{\mathrm{CaL}}$.

This balance of contributions to the CaT is also evident when one considers the effect of individually blocking each flow of Ca^2+^ in the computational model, as seen in Fig. 15 *B*. With the block of $I_{\mathrm{SR}}$, the initial portion of the CaT is blunted, while blocking $I_{\mathrm{CaL}}$ affects both the initial and later phases of the CaT, having the most profound effect on the total change in Ca^2+^. Block of $I_{\mathrm{CaT}}$ or $I_{\mathrm{bCa}}$, on the other hand, has little effect on the CaT. In Fig. 15 *C*, the composition of the CaT is assessed by calculating the percentage contribution of each Ca^2+^ flow to its peak amplitude. This shows that block of $I_{\mathrm{CaL}}$ reduces the CaT peak by 52.2%, whereas the CaT peak is reduced by only 33.4% with block of *I*_SR_ and by 1.2% or 2.2% with block of $I_{\mathrm{CaT}}$ or $I_{\mathrm{bCa}}$.

To directly compare Ca^2+^ handling in the 1D computational model to what has been shown experimentally, we ran simulations mimicking experiments performed by Bovo et al. (2013) in zebrafish isolated ventricular CMs. Figure 15 *D* shows the model output for the simulated experiment, which involved pacing at 2 Hz, followed by a complete release of SR Ca^2+^ – mimicked experimentally using caffeine and simulated computationally by increasing the conductance of $I_{\mathrm{rel}}$ fivefold, until SR calcium was depleted – followed by the resumption of pacing. From this, the amount of Ca^2+^ released from the SR during a normal beat was measured as the difference between CaT amplitude immediately before and after the caffeine‐induced CaT, with the remainder attributed to sarcolemmal Ca^2+^ influx, and the fraction of total SR Ca^2+^ release was calculated by dividing normal SR Ca^2+^ release by the amplitude of the caffeine‐induced CaT. As shown in Fig. 15 *E*, from these measurements we can calculate the relative contribution of sarcolemmal and SR Ca^2+^ flow to the CaT, which indicates that 83.5% of the CaT comes from sarcolemmal currents and only 16.5% comes from the SR, amounting to a fractional release of only 7.2% of total SR Ca^2+^. These values are similar to the findings of Bovo et al. (2013) (fig. 2 of their paper), who measured an 84.1 ± 9.5% contribution of Ca^2+^ currents to the CaT and a 15.9 ± 9.5% contribution of SR Ca^2+^ release, with a fractional SR Ca^2+^ release of ∼9% (Bovo et al., 2013). For comparison, in rabbits sarcolemmal and SR Ca^2+^ contribute 44 ± 16 and 55 ± 13% to the CaT, respectively, with a fractional release of ∼ 40% (Bovo et al., 2013), indicating that in zebrafish ventricular CMs only a small fraction of Ca^2+^ stored in the SR contributes to the CaT.

### Response to channel‐specific block

Table 4 shows the effect of simulated block of $I_{\mathrm{Kr}}$ (as with E4031) or $I_{\mathrm{Ks}}$ (as with chromanol 293B), or combined block of $I_{\mathrm{Na}}$, $I_{\mathrm{Kr}}$ and $I_{\mathrm{Ks}}$ (as with quinidine), on APD in the computational model. For block of $I_{\mathrm{Kr}}$ and $I_{\mathrm{Ks}}$ with E4031 or chromanol 293B, APD prolongation in the model fell within the experimental range previously reported for zebrafish (Abramochkin et al., 2018; De la Cruz et al., 2023; Tsai et al., 2011). In contrast, for multichannel block with quinidine, the model overpredicted APD prolongation compared with experimental findings (Tsai et al., 2011).

**Table 4:** Response to channel specific block. APD, action potential duration

Compound	Concentration [µM]	Block	Model APD_90_ prolongation	Experimental APD_90_ prolongation
E4031	10	78% $I_{\mathrm{Kr}}$	30%	8–31% (Tsai et al., 2011)
Chromanol 293B	10	90% $I_{\mathrm{Ks}}$	14%	11–38% (Tsai et al., 2011)
Quinidine		35% $I_{\mathrm{Na}}$		
	10	97% $I_{\mathrm{Kr}}$	61%	8–30% (Crumb et al., 2016)
		73% $I_{\mathrm{Ks}}$		

## Discussion

We present the development of the first computational model of the zebrafish ventricular AP. Our model incorporates a detailed biophysical parameterization of the principal ionic currents using both published patch‐clamp data and our newly generated recordings from isolated adult zebrafish ventricular cardiomyocytes. In addition, we performed model calibration and validation in both isolated cell (0D) and tissue (1D) simulations using comprehensive experimental data, including microelectrode and fluorescent CaT recordings during steady‐state and restitution pacing protocols in the adult isolated heart.

Characteristics of the experimental AP recordings were within the range of those previously reported for zebrafish whole heart and single cell preparations (Abramochkin et al., 2018; Kompella et al., 2021; Nemtsas et al., 2010; Tsai et al., 2011; Vornanen & Hassinen, 2016). After model calibration using the steady‐state pacing data, all AP characteristics were still within previously reported experimental ranges: (1) APD_90_ = 132–275 ms; (2) APD_50_ = 95–240 ms; (3) $dV/dt{|}_{\max}$ = 18–25 mV/ms; (4) AP amplitude = 90–110 mV; and (5) RMP = −82 to −60 mV (Kompella et al., 2021; Kopton et al., 2018; Nemtsas et al., 2010; Stoyek et al., 2022; Vornanen & Hassinen, 2016).

The model was then validated and further refined using data from the restitution pacing protocols, which also provided novel information about AP and CaT restitution in the zebrafish ventricle. It was found that the zebrafish ventricular AP restitution curves resembled those of human. In both species, APD decreases monotonically in response to a dynamic increase in stimulation frequency. In the zebrafish the maximum reduction in APD was between 22 and 28%, in good agreement with the 25–29% reduction that has been reported for human (O'Hara et al., 2011). In response to an S1–S2 restitution pacing protocol, APD also monotonically decreases in both zebrafish and human, but with the zebrafish showing a smaller percentage reduction (13–15%) compared to human (22–25%) (O'Hara et al., 2011).

While no previous experimental data are available regarding CaT restitution dynamics in zebrafish, our computational model fits well with the novel experimental data generated in the current study. For the model, we used the original TP04 formulation, which, after parameterization and calibration, produced CaT that closely aligned with the experimental data, suggesting similarity to human Ca^2+^ dynamics. Moreover, by comparing CaT using two different fluorescent dyes – one with higher affinity (Rhod‐2) and one with lower affinity (Fluo‐5F) – we demonstrated that the use of higher affinity dyes can lead to artefactually long CaTD, probably due to the buffering of cytosolic Ca^2+^, as reported previously (Babcock et al., 1997; Davidson & Duchen, 2018; Pozzan & Rudolf, 2008; Stamm et al., 2003). While the lower affinity dye presumably produced more faithful CaT, its use was limited, as the signals are less strong, and degradation with photobleaching limits the number of possible simultaneous recordings. As such, it did not work with the S1–S2 restitution protocol, and the number of tested cycle lengths with the dynamics protocol was reduced. Yet, our experimental and computational measurements agreed well with previously reported experimental data: (1) CaTD_80_ = 200–261 ms; (2) CaTD_50_ = 117–140 ms; (3) CaTD_20_ = 110–135 ms; and (4) $dF/dt{|}_{\max}$ = 0.03–0.09 ms^−1^ (Lin et al., 2015; Rayani et al., 2018; Stoyek et al., 2024).

There are well‐described limitations of the zebrafish as a model for cardiac Ca^2+^ handling, as important differences exist compared to human and other mammals (van Opbergen et al., 2018). These differences limited our group's previous efforts to create a zebrafish‐specific computational model of the ventricular AP (Cestariolo et al., 2022; Cestariolo, Ferrero, et al., 2023). Cestariolo et al. (2022) implemented the TP06 formulation of the human AP as a base for their model. However, the more complex Ca^2+^ handling dynamic included in the TP06 formulation was not appropriate for zebrafish. Thus, Cestariolo et al. (2023) instead utilized the TP04 formulation. However, there was a lack of sufficient experimental data for its parameterization and calibration. A major update from that preliminary work is presented here, including a full model parameterization based on our novel CaT restitution data, which allowed us to further explore Ca^2+^ mechanisms in zebrafish ventricular CMs. Using our novel computational model, we examined the flows of Ca^2+^ contributing to the CaT, which showed that a majority comes from sarcolemmal influx via $I_{\mathrm{CaL}}$, as suggested by previous experimental work (Haverinen, Hassinen, Dash, et al., 2018). In contrast, Ca^2+^ release from the SR plays a smaller role in the CaT of zebrafish, even though SR Ca^2+^ concentration is high, due to a relatively small fractional release, as demonstrated previously in zebrafish isolated ventricular CMs (Bovo et al., 2013). This finding helps explain why $I_{\mathrm{NaCa}}$ is larger in zebrafish than human, despite a conserved genetic sequence for NCX1 (van Opbergen et al., 2018). Our model also allowed us to predict the absolute systolic concentration of Ca^2+^ in zebrafish ventricular CMs as 1.02 µM, and while there are no reported experimental values for comparison, this concentration is similar to that reported in human (1.0–1.5 µM; Bers, 2000). Thus, while different from that of mammals, the Ca^2+^ handling in our computational AP model is in agreement with what is currently known for zebrafish.

It is also important to consider the currents responsible for AP repolarization in zebrafish compared to human and other mammals. The computational model developed in this study includes $I_{\mathrm{Ks}}$, despite some studies suggesting that it may not be present in zebrafish (Alday et al., 2014; Nemtsas et al., 2010). A recent study, however, indicated that AP repolarization in zebrafish involves $I_{\mathrm{Ks}}$, produced by homotetramers of the Kv7.1 channel without the MinK β‐subunit (in contrast to human, where MinK is an essential component) (Abramochkin et al., 2018). It was further shown that $I_{\mathrm{Ks}}$ has a one order of magnitude lower maximal conductance than $I_{\mathrm{Kr}}$, the most prominent repolarizing current in zebrafish (Vornanen & Hassinen, 2016). In our computational model, the $I_{\mathrm{Ks}}$:$I_{\mathrm{Kr}}$ maximal conductance ratio ($G_{\mathrm{Ks}}$:$G_{\mathrm{Kr}}$) is 1:12, which agrees well with the previous experimental findings (Abramochkin et al., 2018; Hassinen, Haverinen, & Vornanen, 2015; Hassinen, Haverinen, et al., 2015), although the actual current amplitudes during the AP are more similar (Fig. 10). Furthermore, with block of $I_{\mathrm{Ks}}$ or $I_{\mathrm{Kr}}$, it correctly reproduced the previously reported experimental result (Abramochkin et al., 2018; Tsai et al., 2011). Yet, while the model accurately predicted the effects of blocking single currents (for which specific dose–response data for the zebrafish were available), with results similar to that seen in human, it predicted a greater AP prolongation for multi‐channel block with quinidine than in human (Tsai et al., 2011). However, as data on quinidine's effects in zebrafish are not available, dose–response parameters for human were used for parameterizing the simulations (Crumb et al., 2016). Thus, the discrepancy with the experimental results highlights that despite similarities of the AP between human and zebrafish, care should be taken with the use of zebrafish for pharmacological screens, as there may be different sensitivity to some compounds between the two species (Bett et al., 2006; Genge et al., 2024).

An important result of the current study stems from the comparison of our single cell (0D) and tissue (1D) models. Both reproduced similar APD under steady‐state and restitution pacing protocols, in agreement with previous work (Carro et al., 2017; O'Hara et al., 2011). However, the upstroke speed and APA differed between the models, such that only the 1D restitution simulations matched the whole heart experimental AP data. This highlights the importance of calibrating models using simulation protocols that most closely match those used experimentally. In the current study, we incorporated both whole‐heart experimental measurements and single‐cell data from patch‐clamp experiments. The whole‐heart data, which would include influences of electrotonic coupling as measurements are made in intact tissue, resulted in the 1D model more closely replicating our experimental findings. In contrast, the 0D model – replicating isolated cells that lack electrotonic interactions – did not fully recapitulate the behaviour observed in the intact heart.

**Figure A1:**
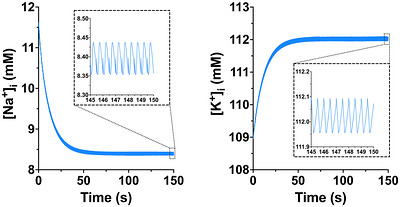
Intracellular concentrations of sodium and potassium (${\left[Na^{+}\right]}_{i}$ and ${\left[K^{+}\right]}_{i}$) over time in the computational model These traces highlight that ${\left[Na^{+}\right]}_{i}$ and ${\left[K^{+}\right]}_{i}$ reach a steady state, confirming the homeostatic function of the model over long‐term simulations. The model was paced at 2 Hz, which leads to oscillations in ionic concentrations during the cardiac cycle, due to cyclical ion fluxes associated with the AP. Stabilization of ionic concentrations is essential for ensuring the physiological relevance and numerical stability of the model.

Considering the limitations of our study, it is important to recognize that direct measurements of some currents, such as $I_{\mathrm{NaK}}$ and $I_{\mathrm{NaCa}}$, in adult zebrafish are lacking. Therefore, the original formulations from the TP04 model were utilized, and their validity was evaluated through specific biophysical properties of the model. In particular, the ability of the model to stabilize intracellular Na^+^ and K^+^ at different stimulation frequencies was verified. At a pacing rate of 2 Hz, after 100 s, intracellular Na^+^ stabilized at 7.99 mm, and intracellular K^+^ stabilized at 112.47 mm (see Fig. A1 and Table A2 in Appendix 2). Future measurements of the rate dependence of intracellular Na^+^ in the adult zebrafish heart will allow for better calibration and validation of the model. Furthermore, a lack of sufficient experimental data regarding the kinetics of other cardiac ion channels in the zebrafish (e.g. $I_{\mathrm{CaT}}$) prevented the sufficient development of species‐specific current formulations, necessitating the use of those proposed for other species. While our model includes a small Na^+^ permeability in the calculation of the reversal potential for $I_{\mathrm{Ks}}$, Na^+^ flux through $I_{\mathrm{Ks}}$ is not incorporated into the intracellular Na^+^ balance equations, in line with the TP04 model and several modern human cardiac models (e.g. O'Hara et al., 2011; Tomek et al., 2019). This simplification is commonly adopted in AP models due to the relatively minor flux of Na^+^ through $I_{\mathrm{Ks}}$, and is unlikely to have a large effect on intracellular ion homeostasis; however, it represents a modelling assumption that could be revisited in future work should experimental evidence suggest otherwise.

Despite these limitations, we have developed the first computational model of the adult zebrafish ventricular AP that accurately reproduces experimentally measured steady‐state AP and CaT and key restitution properties. The model's Ca^2+^ handling dynamics replicate experimental observations, and model simulations robustly capture the effects of specific ion channel blockade. Overall, this model fills a critical gap in available tools for studying zebrafish cardiac electrophysiology, providing a mechanistic framework to interpret experimental findings and generate novel hypothesis. While future experimental advances will allow for further refinement, the model in its current form represents a useful tool for future studies of cardiac electrophysiology and its dysfunction.

## Additional information

## Competing interests

None declared.

## Author contributions

L.C.: Study conceptualization, Methodological development, Software generation, Computational simulations, Data analysis, Writing – original draft, review, and editing. Z.D.L.: Study conceptualization, Methodological development, Experimental work, Data analysis, Writing – original draft, review, and editing. A.O.V.: Experimental work, Data analysis, Writing – review and editing. J.M.F.: Study conceptualization, Methodological developments, Writing – review and editing. T.A.Q.: Study conceptualization, Methodological developments, Experimental supervision, Funding acquisition, Project administration, Writing – review and editing. J.F.R.M.: Study conceptualization, Methodological developments, Software generation, Computational supervision, Funding acquisition, Project administration, Writing – review and editing.

## Funding

L.C. and J.F.R.M. are supported by a grant from the Italian Ministry of Education, University and Research (1613 FISR2019_03221, CECOMES). J.M.F. is supported by a grant from Ministerio de Ciencia e Innovación (PID2022‐140553OB‐C41, MICIU/AEI/10.13039/501100011033) and by the European Union's Horizon 2020 Research and Innovation Program (No. 101016496, SimCardioTest). Z.D.L. is supported by a Mitacs Globalink Research Award (IT38494), the Dalhousie Faculty of Medicine (MacDonald Heart & Diabetes Research Studentship), the Government of Nova Scotia (Nova Scotia Graduate Scholarship), and by a Canada Graduate Scholarship – Doctoral from the Natural Sciences and Engineering Research Council of Canada (CGS D – 601186 – 2025). T.A.Q. is supported by grants from the Natural Sciences and Engineering Research Council of Canada (RGPIN‐2022‐03150), the Government of Canada's New Frontiers in Research Fund (NFRFE‐1269 2021‐00219), the Canadian Institutes of Health Research (PJT‐185904 and PJT‐190009), and the Heart and Stroke Foundation of Canada (G‐22‐0032127).

## Supporting information


Peer Review History

## Data Availability

The authors confirm that the data supporting the findings of this study are available within the article [and/or] appropriate repositories.

## Appendix 1

### Reversal potentials

(A1)
$$E_{\mathrm{Na}}=\frac{RT}{F}\;\log\left(\frac{{\left[Na^{+}\right]}_{o}}{{\left[Na^{+}\right]}_{i}}\right)$$

(A2)
$$E_{\mathrm{Ca}}=\frac{RT}{2F}\;\log\left(\frac{{\left[Ca^{2+}\right]}_{o}}{{\left[Ca^{2+}\right]}_{i}}\right)$$

(A3)
$$E_{K}=\frac{RT}{F}\;\log\left(\frac{{\left[K^{+}\right]}_{o}}{{\left[K^{+}\right]}_{i}}\right)$$

(A4)
$$E_{\mathrm{Ks}}=\frac{RT}{F}\;\log\left(\frac{{\left[K^{+}\right]}_{o}+\rho_{\mathrm{KNa}}{\left[Na^{+}\right]}_{o}}{{\left[K^{+}\right]}_{i}+\rho_{\mathrm{KNa}}{\left[Na^{+}\right]}_{i}}\right)$$

### Fast Na^+^ current

(A5)
$$I_{\mathrm{Na}}=G_{\mathrm{Na}}m^{3}hj\left(V_{m}-E_{\mathrm{Na}}\right)$$

(A6)
$$m_{\infty }=\frac{1}{1+e^{-\left(\frac{V_{m}+58.5}{8.025}\right)}}\;$$

(A7)
$$\alpha_{m}=\frac{1}{1+e^{-\left(\frac{V_{m}+80}{5}\right)}}\;$$

(A8)
$$\beta_{m}=\left(\frac{1.95}{1+e^{\frac{V_{m}+45}{6}}}+\frac{0.1}{1+e^{\frac{V_{m}-60}{200}}}\right)$$

(A9)
$$\tau_{m}=0.9395\alpha_{m}\beta_{m}$$

(A10)
$$h_{\infty }=\frac{1}{1+e^{\frac{V_{m}+70.5727}{7.5}}}\;$$

(A11)
$$\alpha_{h}=\left\{\begin{array}{ccc} 0 & \mathrm{if} & V_{m}\geq -40\;mV\\ 0.057e^{-\left(\frac{V_{m}+80}{6.8}\right)} & \mathrm{if} & V_{m}<-40\;mV \end{array}\right.\;$$

(A12)
$$\beta_{h}=\left\{\begin{array}{ccc} \frac{0.77}{0.13\cdot \left(1+e^{-\left(\frac{V_{m}+10.66}{11.1}\right)}\right)} & \mathrm{if} & V_{m}\geq -40\;mV\\ 2.7e^{0.079V_{m}}+3.1\cdot 10^{5}e^{0.3485V_{m}} & \mathrm{if} & V_{m}<-40\;mV \end{array}\right.\;$$

(A13)
$$\tau_{h}=\frac{3.5}{\alpha_{h}+\beta_{h}}\;$$

(A14)
$$j_{\infty }=\frac{1}{1+e^{\frac{V_{m}+70.5727}{7.5}}}\;$$

(A15)
$$\alpha_{j}=\left\{\begin{array}{ccc} 0 & \mathrm{if} & V_{m}\geq -31\;mV\\ \frac{\left(-2.5428\cdot 10^{4}e^{0.2444\left(V_{m}-9\right)}-6.948\cdot 10^{-6}e^{-0.04391\left(V_{m}-9\right)}\right)\left(V_{m}+28.78\right)}{1+e^{0.311\left(V_{m}+70.23\right)}} & \mathrm{if} & V_{m}<-31\;mV \end{array}\right.\;$$

(A16)
$$\beta_{j}=\left\{\begin{array}{ccc} \frac{0.6\cdot e^{0.057\left(V_{m}-9\right)}}{1+e^{-0.1\left(V_{m}+23\right)}} & \mathrm{if} & V_{m}\geq -31\;mV\\ \frac{0.02424\cdot e^{-0.01052\left(V_{m}-9\right)}}{1+e^{-0.1378\left(V_{m}+31.14\right)}} & \mathrm{if} & V_{m}<-31\;mV \end{array}\right.\;$$

(17)
$$\tau_{j}=\frac{0.8}{\alpha_{j}+\beta_{j}}\;$$

### T‐type Ca^2+^ current

(A18)
$$I_{\mathrm{CaT}}=G_{\mathrm{CaT}}\;d_{T}f_{T}\left(V_{m}-E_{\mathrm{Ca}}\right)$$

(A19)
$$d_{T\infty }=\frac{1}{1+e^{-\frac{V_{m}+30}{8.5}}}\;$$

(A20)
$$\tau_{\mathrm{dT}}=\frac{1}{1.068e^{\frac{V_{m}+26.3}{30}}+1.068e^{-\;\frac{V_{m}+26.3}{30}}}\;$$

(A21)
$$f_{T\infty }=\frac{1}{1+e^{\frac{V_{m}+71}{9}}}\;$$

(A22)
$$\tau_{\mathrm{fT}}=\frac{1000}{15.3e^{-\frac{V_{m}+71.7}{83.3}}+15e^{\frac{V_{m}+71.7}{15.38}}\;}\;$$

### L‐type Ca^2+^ current

(A23)
$$\begin{array}{ccc} I_{\mathrm{CaL}} & = & 4G_{\mathrm{CaL}}\;d_{L}f_{L}f_{\mathrm{Ca}}\frac{V_{m}F^{2}}{RT}\\ & & \frac{{\left[Ca^{2+}\right]}_{i}\;e^{\frac{2V_{m}F}{RT}}-0.341{\left[Ca^{2+}\right]}_{o}}{e^{\frac{2V_{m}F}{RT}}-1} \end{array}$$

(A24)
$$d_{L\infty }=\frac{1}{1+e^{-\;\frac{V_{m}+25}{5.478}}}\;$$

(A25)
$$\alpha_{\mathrm{dL}}=2.93+\frac{2.8}{1+e^{-\frac{V_{m}+20}{13}}}$$

(A26)
$$\beta_{\mathrm{dL}}=\frac{1.4}{1+e^{\frac{V_{m}+5}{5}}}\;$$

(A27)
$$\gamma_{\mathrm{dL}}=\frac{3.5}{1+e^{-\frac{V_{m}+5}{10}}}\;$$

(A28)
$$\tau_{\mathrm{dL}}=0.756\left(\alpha_{\mathrm{dL}}\beta_{\mathrm{dL}}+\gamma_{\mathrm{dL}}\right)$$

(A29)
$$f_{L\infty }=\frac{1}{1+e^{\frac{V_{m}+35.8779}{4}}}\;$$

(A30)
$$\alpha_{\mathrm{fL}}=310.5e^{-\frac{{\left(V_{m}+38\right)}^{2}}{120}}$$

(A31)
$$\beta_{\mathrm{fL}}=\frac{80}{1+e^{-\frac{V_{m}-8}{10}}}\;$$

(A32)
$$\gamma_{\mathrm{fL}}=\frac{120}{1+e^{\frac{V_{m}+35}{10}}}+20$$

(A33)
$$\tau_{\mathrm{fL}}=\alpha_{\mathrm{fL}}+\beta_{\mathrm{fL}}+\gamma_{\mathrm{fL}}$$

(A34)
$$\alpha_{\mathrm{fCa}}=\frac{1}{1+{\left(\frac{{\left[Ca^{2+}\right]}_{i}}{0.000325}\right)}^{8}}\;$$

(A35)
$$\beta_{\mathrm{fCa}}=\frac{0.1}{1+e^{\frac{{\left[Ca^{2+}\right]}_{i}-0.0005}{0.0001}}}\;$$

(A36)
$$\gamma_{\mathrm{fCa}}=\frac{0.2}{1+e^{\frac{{\left[Ca^{2+}\right]}_{i}-0.00075}{0.0008}}}\;$$

(A37)
$$f_{\mathrm{Ca}\infty }=\frac{\alpha_{\mathrm{fCa}}+\beta_{\mathrm{fCa}}+\gamma_{\mathrm{fCa}+0.23}}{1.45}\;$$

(A38)
$$\tau_{\mathrm{fCa}}=2\;ms$$

(A39)
$$\begin{array}{ccc} & & \frac{df_{\mathrm{Ca}}}{dt}=\\ & & \left\{\begin{array}{ccc} 0 & \mathrm{if} & \left(f_{\mathrm{Ca}\infty }>f_{\mathrm{Ca}}\right)\;\mathrm{and}\;\left(V_{m}>-60\;mV\right)\\ \frac{f_{\mathrm{Ca}\infty }-f_{\mathrm{Ca}}}{\tau_{\mathrm{fCa}}} & & \mathrm{otherwise} \end{array}\right.\; \end{array}$$

### Rapid delayed rectifier K^+^ current

(A40)
$$I_{\mathrm{Kr}}=G_{\mathrm{Kr}}\;\sqrt{\frac{{\left[K^{+}\right]}_{o}}{4.85}}x_{r1}x_{r2}\left(V_{m}-E_{K}\right)$$

(A41)
$$x_{r1\infty }=\frac{1}{1+e^{-\frac{V_{m}-\;8.87}{6.56}}}\;$$

(A42)
$$\alpha_{\mathrm{xr}1}=\frac{690}{1+e^{-\frac{V_{m}+42}{10}}}\;$$

(A43)
$$\beta_{\mathrm{xr}1}=\frac{6}{1+e^{\frac{V_{m}+49}{11.5}}}+0.09$$

(A44)
$$\tau_{\mathrm{xr}1}=1.0798\cdot \alpha_{\mathrm{xr}1}\beta_{\mathrm{xr}1}$$

(A45)
$$x_{r2\infty }=\frac{1}{1+e^{\frac{V_{m}+85.07}{22.82}}}\;$$

(A46)
$$\alpha_{\mathrm{xr}2}=65e^{-\frac{{\left(V_{m}+85\right)}^{2}}{700}}$$

(A47)
$$\beta_{\mathrm{xr}2}=\frac{5}{1+e^{\frac{V_{m}-5}{15}}}+3$$

(A48)
$$\tau_{\mathrm{xr}2}=0.9443\left(\alpha_{\mathrm{xr}2}+\beta_{\mathrm{xr}2}\right)$$

### Slow delayed rectifier K^+^ current

(A49)
$$I_{\mathrm{Ks}}=G_{\mathrm{Ks}}\;x_{s}\left(V_{m}-E_{\mathrm{Ks}}\right)$$

(A50)
$$x_{s\infty }=\frac{1}{1+e^{-\;\frac{V_{m}-4.9182}{30}}}\;$$

(A51)
$$\alpha_{\mathrm{xs}}=\frac{670}{1+e^{-\;\frac{V_{m}+35}{6}}}\;$$

(A52)
$$\beta_{\mathrm{xs}}=\frac{1}{1+e^{\frac{V_{m}-5}{21}}}\;$$

(A53)
$$\tau_{\mathrm{xs}}=\alpha_{\mathrm{xs}}\;\beta_{\mathrm{xs}}$$

### Inward rectifier K^+^ current

(A54)
$$I_{K1}=G_{K1}\;\sqrt{\frac{{\left[K^{+}\right]}_{o}}{4.85}}x_{K1\infty }\left(V_{m}-E_{K}\right)$$

(A55)
$$x_{K1\infty }=\frac{1}{1+e^{\frac{V_{m}+87.0762}{13.5048}}}+0.02$$

### *Na^+^/Ca* ^2+^ exchanger current

(A56)
$$I_{\mathrm{NaCa}}=k_{\mathrm{NaCa}}\;\cdot \frac{e^{\gamma V_{m}F/RT}{\left[Na^{+}\right]}_{i}^{3}{\left[Ca^{2+}\right]}_{o}-\alpha e^{\left(\gamma -1\right)V_{m}F/RT}{\left[Na^{+}\right]}_{o}^{3}{\left[Ca^{2+}\right]}_{i}}{\left(K_{\mathrm{mNai}}^{3}+{\left[Na^{+}\right]}_{o}^{3}\right)\left(K_{\mathrm{mCa}}+{\left[Ca^{2+}\right]}_{o}\right)\left(1+k_{\mathrm{sat}}e^{\left(\gamma -1\right)V_{m}F/RT}\right)}$$

### Na^+^/K^+^ pump current

(A57)
$$I_{\mathrm{NaK}}=P_{\mathrm{NaK}}\;\frac{{\left[K^{+}\right]}_{o}{\left[Na^{+}\right]}_{i}}{\left({\left[K^{+}\right]}_{o}+K_{\mathrm{mK}}\right)\left({\left[Na^{+}\right]}_{i}+K_{\mathrm{mNa}}\right)\left(1+0.1245e^{-\frac{0.1V_{m}F}{RT}}+0.0353e^{-V_{m}F/RT}\right)}$$

### Sarcolemma Ca^2+^ pump

(A58)
$$I_{\mathrm{pCa}}=G_{\mathrm{pCa}}\;\frac{{\left[Ca^{2+}\right]}_{i}}{K_{\mathrm{pCa}}+{\left[Ca^{2+}\right]}_{i}}$$

### Background currents

(A59)
$$I_{\mathrm{bNa}}=G_{\mathrm{bNa}}\;\left(V_{m}-E_{\mathrm{Na}}\right)$$

(A60)
$$I_{\mathrm{bCa}}=G_{\mathrm{bCa}}\;\left(V_{m}-E_{\mathrm{Ca}}\right)$$

### Intracellular Ca^2+^ dynamics

(A61)
$$I_{\mathrm{leak}}=V_{\mathrm{leak}}\;\left({\left[Ca^{2+}\right]}_{\mathrm{sr}}-{\left[Ca^{2+}\right]}_{i}\right)$$

(A62)
$$I_{\mathrm{up}}=\frac{V_{\mathrm{maxup}}}{1+{\left(K_{\mathrm{up}}/{\left[Ca^{2+}\right]}_{i}\right)}^{2}}\;$$

(A63)
$$I_{\mathrm{rel}}=\left(a_{\mathrm{rel}}\cdot \frac{{\left[Ca^{2+}\right]}_{\mathrm{sr}}^{2}}{b_{\mathrm{rel}}^{2}+{\left[Ca^{2+}\right]}_{\mathrm{sr}}^{2}}+c_{\mathrm{rel}}\right)\;d_{L}g$$

(A64)
$$g_{\infty }=\left\{\begin{array}{ccc} \frac{1}{1+{\left({\left[Ca^{2+}\right]}_{i}/0.00035\right)}^{6}} & \mathrm{if} & Ca_{i}\leq 0.00035\;mM\\ \frac{1}{1+{\left({\left[Ca^{2+}\right]}_{i}/0.00035\right)}^{16}} & & \mathrm{otherwise} \end{array}\right.\;$$

(A65)
$$\tau_{g}=3\;ms$$

(A66)
$$\frac{dg}{dt}=\left\{\begin{array}{ccc} 0 & \mathrm{if} & g_{\infty }>g\;\mathrm{and}\;V_{m}>-60\;mV\\ \frac{g_{\infty }-g}{\tau_{g}} & & \mathrm{otherwise} \end{array}\right.\;$$

(A67)
$$Ca_{\mathrm{ibufc}}=\frac{{\left[Ca^{2+}\right]}_{i}\times buf_{c}}{{\left[Ca^{2+}\right]}_{i}+kbuf_{c}}\;$$

(A68)
$$\begin{array}{ccc} & & \frac{dCa_{\mathrm{itotal}}}{dt}=-\frac{I_{\mathrm{CaL}}+I_{\mathrm{CaT}}+I_{\mathrm{bCa}}+I_{\mathrm{pCa}}-2I_{\mathrm{NaCa}}}{2V_{c}F}C_{m}A_{c}\\ & & +\;I_{\mathrm{leak}}-I_{\mathrm{up}}+I_{\mathrm{rel}} \end{array}$$

(A69)
$$Ca_{\mathrm{srbufsr}}=\frac{{\left[Ca^{2+}\right]}_{\mathrm{sr}}\times buf_{\mathrm{sr}}}{{\left[Ca^{2+}\right]}_{\mathrm{sr}}+kbuf_{\mathrm{sr}}}\;$$

(A70)
$$\frac{dCa_{\mathrm{srtotal}}}{dt}=\frac{V_{C}}{V_{\mathrm{SR}}}\;\cdot \left(I_{\mathrm{up}}-I_{\mathrm{leak}}-I_{\mathrm{rel}}\right)$$

### Intracellular sodium and potassium dynamics

(A71)
$$\frac{dNa_{i}}{dt}=-\frac{I_{\mathrm{Na}}+I_{\mathrm{bNa}}+3I_{\mathrm{NaK}}+3I_{\mathrm{NaCa}}}{V_{c}F}C_{m}A_{c}$$

(A72)
$$\frac{dK_{i}}{dt}=-\frac{I_{K1}+I_{\mathrm{Kr}}+I_{\mathrm{Ks}}-2I_{\mathrm{NaK}}+I_{\mathrm{stim}}}{V_{c}F}C_{m}A_{c}$$

## Appendix 2

**Table A1:** Model parameters

Parameter	Value
Gas constant – R	8314.472 J/(K kmol)
Temperature – T	301 K
Faraday constant – F	96,485.3415 C/mol
Tissue diffusion coefficient – D	1.76 × 10^−2^ mm^2^/ms
Membrane capacitance – *C*_m_	1.0 µF/cm^2^
Cytoplasmic volume – $V_{c}$	390 µm^3^
Sarcoplasmic reticulum volume – $V_{\mathrm{SR}}$	0.05 V_c_ µm^3^
Capacitive area – $A_{c}$	3000 µm^2^
Extracellular K^+^ concentration – ${\left[K^{+}\right]}_{o}$	4.85 mM
Extracellular Na^+^ concentration – ${\left[Na^{+}\right]}_{o}$	142 mM
Extracellular Ca^2+^ concentration – ${\left[Ca^{2+}\right]}_{o}$	1.8 mM
Maximal $I_{\mathrm{Na}}$ conductance – $G_{\mathrm{Na}}$	1.0 mS/µF
Maximal $I_{K1}$ conductance – $G_{K1}$	0.3490 mS/µF
Maximal $I_{\mathrm{Kr}}$ conductance – $G_{\mathrm{Kr}}$	0.7776 mS/µF
Maximal $I_{\mathrm{Ks}}$ conductance – $G_{\mathrm{Ks}}$	0.0648 mS/µF
Relative $I_{\mathrm{Ks}}$ permeability to Na^+^ – $p_{\mathrm{KNa}}$	0.03
Maximal $I_{\mathrm{CaL}}$ conductance – $G_{\mathrm{CaL}}$	2.3843 × 10^−4^ cm^3^/(s µF)
Maximal $I_{\mathrm{NaCa}}$ – $k_{\mathrm{NaCa}}$	1000 µA/µF
Voltage dependence parameter of $I_{\mathrm{NaCa}}$ – γ	0.35
${\left[Ca^{2+}\right]}_{i}$ half‐saturation constant for $I_{\mathrm{NaCa}}$ – $K_{\mathrm{mCai}}$	1.38 mM
${\left[Na^{+}\right]}_{i}$ half‐saturation constant for $I_{\mathrm{NaCa}}$ – $K_{\mathrm{mNai}}$	87.5 mM
Saturation factor for $I_{\mathrm{NaCa}}$ – $k_{\mathrm{sat}}$	0.1
Factor enhancing outward nature of $I_{\mathrm{NaCa}}$ – α	2.5
Maximal $I_{\mathrm{CaT}}$ conductance – $G_{\mathrm{CaT}}$	0.066 mS/µF
Maximal $I_{\mathrm{NaK}}$ – $P_{\mathrm{NaK}}$	4.086 µA/µF
${\left[K^{+}\right]}_{o}$ half‐saturation of $I_{\mathrm{NaK}}$ – $K_{\mathrm{mK}}$	1.0 mM
${\left[Na^{+}\right]}_{i}$ half‐saturation of $I_{\mathrm{NaK}}$ – $K_{\mathrm{mNa}}$	40.0 mM
Maximal $I_{\mathrm{pCa}}$ conductance – $G_{\mathrm{pCa}}$	0.0456 mS/µF
Half‐saturation constant of $I_{\mathrm{pCa}}$ – $K_{\mathrm{pCa}}$	0.0005 mM
Maximal $I_{\mathrm{bNa}}$ conductance – $G_{\mathrm{bNa}}$	3.8137 × 10^−4^ mS/µF
Maximal $I_{\mathrm{bCa}}$ conductance – $G_{\mathrm{bCa}}$	6.6387 × 10^−4^ mS/µF
Maximal $I_{\mathrm{up}}$ conductance – $V_{\mathrm{maxup}}$	0.000425 mM/ms
Half‐saturation constant of $I_{\mathrm{up}}$ – $K_{\mathrm{up}}$	0.00025 mM
Maximal Ca_SR_‐dependent $I_{\mathrm{rel}}$ – $a_{\mathrm{rel}}$	0.0132 mM/ms
${\left[Ca^{2+}\right]}_{\mathrm{SR}}$ half‐saturation constant of $I_{\mathrm{rel}}$ – $b_{\mathrm{rel}}$	0.25 mM
Maximal Ca_SR_‐independent $I_{\mathrm{rel}}$ – $c_{\mathrm{rel}}$	0.0066 mM/ms
Maximal $I_{\mathrm{leak}}$ conductance – $V_{\mathrm{leak}}$	8 × 10^−5^ mM/ms
Total cytoplasmic buffer concentration – $buf_{c}$	0.19 mM
${\left[Ca^{2+}\right]}_{i}$ half‐saturation constant for cytoplasmic buffer – $kbuf_{c}$	0.001 mM
Total sarcoplasmic buffer concentration – $buf_{\mathrm{sr}}$	10 mM
${\left[Ca^{2+}\right]}_{\mathrm{SR}}$ half‐saturation constant for sarcoplasmic buffer – $kbuf_{\mathrm{sr}}$	0.3 mM

**Table A2:** Initial parameters for the state variables in the model; in parentheses (.) is the steady‐state value at BCL = 500 ms

Parameter	Value
Transmembrane potential – $V_{m}$	−80.52 (−80.61) mV
Intracellular Na^+^ concentration – ${\left[Na^{+}\right]}_{i}$	11.60 (7.99) mM
Intracellular K^+^ concentration – ${\left[K^{+}\right]}_{i}$	109 (112.47) mM
Intracellular Ca^2+^ concentration – ${\left[Ca^{2+}\right]}_{i}$	2 × 10^−4^ (4.69 × 10^−5^) mM
Sarcoplasmic reticulum Ca^2+^ concentration – ${\left[Ca^{2+}\right]}_{\mathrm{sr}}$	0.20 (0.40) mM
$I_{\mathrm{Na}}$ activation gate – m	0.00 (5.98 × 10^−2^)
$I_{\mathrm{Na}}$ fast inactivation gate – h	0.75 (0.79)
$I_{\mathrm{Na}}$ slow inactivation gate – j	0.75 (0.79)
$I_{\mathrm{Kr}}$ activation gate – $x_{r1}$	0.00 (9.60 × 10^−3^)
$I_{\mathrm{Kr}}$ inactivation gate – $x_{r2}$	0.10 (0.45)
$I_{\mathrm{Ks}}$ activation gate – $x_{s}$	0.00 (5.46 × 10^−2^)
$I_{\mathrm{CaL}}$ activation gate – $d_{L}$	0.00 (3.90 × 10^−5^)
$I_{\mathrm{CaL}}$ inactivation gate – $f_{L}$	1.00 (0.93)
$I_{\mathrm{CaL}}$ intracellular Ca^2+^‐dependent inactivation gate – $f_{\mathrm{Ca}}$	1.00 (1.00)
$I_{\mathrm{CaT}}$ activation gate – $d_{T}$	2.60 × 10^−3^ (2.60 × 10^−3^)
$I_{\mathrm{CaT}}$ inactivation gate – $f_{T}$	0.74 (0.74)
$I_{\mathrm{rel}}$ intracellular calcium‐dependent inactivation gate – g	1.00 (1.00)

## References

[tjp16866-bib-0001] Abramochkin, D. V. , Hassinen, M. , & Vornanen, M. (2018). Transcripts of Kv7.1 and MinK channels and slow delayed rectifier K^+^ current (IKs) are expressed in zebrafish (Danio rerio) heart. Pflugers Archiv: European journal of Physiology, 470(12), 1753–1764. 30116893 10.1007/s00424-018-2193-1

[tjp16866-bib-0002] Alday, A. , Alonso, H. , Gallego, M. , Urrutia, J. , Letamendia, A. , Callol, C. , & Casis, O. (2014). Ionic channels underlying the ventricular action potential in zebrafish embryo. Pharmacological Research, 84, 26–31. 24747832 10.1016/j.phrs.2014.03.011

[tjp16866-bib-0003] Babcock, D. F. , Herrington, J. , Goodwin, P. C. , Park, Y. B. , & Hille, B. (1997). Mitochondrial participation in the intracellular Ca^2+^ network. Journal of Cell Biology, 136(4), 833–844. 9049249 10.1083/jcb.136.4.833 PMC2132502

[tjp16866-bib-0004] Bartolucci, C. , Passini, E. , Hyttinen, J. , Paci, M. , & Severi, S. (2020). Simulation of the effects of extracellular calcium changes leads to a novel computational model of human ventricular action potential with a revised calcium handling. Frontiers in Physiology, 11(314), 1–20. 32351400 10.3389/fphys.2020.00314 PMC7174690

[tjp16866-bib-0005] Beeler, G. W. , & Reuter, H. (1977). Reconstruction of the action potential of ventricular myocardial fibres. The Journal of Physiology, 268(1), 177–210. 874889 10.1113/jphysiol.1977.sp011853 PMC1283659

[tjp16866-bib-0006] Bers, D. M. (2000). Calcium fluxes involved in control of cardiac myocyte contraction. Circulation Research, 87(4), 275–281. 10948060 10.1161/01.res.87.4.275

[tjp16866-bib-0007] Bett, G. C. L. , Morales, M. J. , Beahm, D. L. , Duffey, M. E. , & Rasmusson, R. L. (2006). Ancillary subunits and stimulation frequency determine the potency of chromanol 293B block of the KCNQ1 potassium channel. The Journal of Physiology, 576(3), 755–767. 16887873 10.1113/jphysiol.2006.116012 PMC1890396

[tjp16866-bib-0008] Bovo, E. , Dvornikov, A. V. , Mazurek, S. R. , de Tombe, P. P. , & Zima, A. V. (2013). Mechanisms of Ca^2+^ handling in zebrafish ventricular myocytes. Pflugers Archiv: European Journal of Physiology, 465(12), 1775–1784. 23821298 10.1007/s00424-013-1312-2 PMC4138713

[tjp16866-bib-0009] Brette, F. , Luxan, G. , Cros, C. , Dixey, H. , Wilson, C. , & Shiels, H. A. (2008). Characterization of isolated ventricular myocytes from adult zebrafish (Danio rerio). Biochemical and Biophysical Research Communications, 374(1), 143–146. 18602892 10.1016/j.bbrc.2008.06.109 PMC2581121

[tjp16866-bib-0010] Carro, J. , Rodríguez Matas, J. F. , Monasterio, V. , & Pueyo, E. (2017). Limitations in electrophysiological model development and validation caused by differences between simulations and experimental protocols. Progress in Biophysics and Molecular Biology, 129, 53–64. 27899270 10.1016/j.pbiomolbio.2016.11.006

[tjp16866-bib-0011] Cestariolo, L. , Ferrero, J. M. , Long, Z. , Quinn, T. A. , & Rodriguez Matas, J. F. (2023). Towards the development of an in silico model for the zebrafish action potential. In 2023 Computing in Cardiology (CinC), 50, 1–4.

[tjp16866-bib-0012] Cestariolo, L. , Luraghi, G. , L'Eplattenier, P. , & Rodriguez Matas, J. F. (2023). A finite element model of the embryonic zebrafish heart electrophysiology. Computer Methods and Programs in Biomedicine, 229, 1–8. 10.1016/j.cmpb.2022.107281 36470034

[tjp16866-bib-0013] Cestariolo, L. , Martinez, M. B. , Ferrero, J. M. , & Rodriguez Matas, J. F. (2022). A model for zebrafish ventricular action potential. In 2022 Computing in Cardiology (CinC), 49, 1–4.

[tjp16866-bib-0014] Chiang, D. Y. , Verkerk, A. O. , Victorio, R. , Shneyer, B. I. , van der Vaart, B. , Jouni, M. , Narendran, N. , Kc, A. , Sampognaro, J. R. , Vetrano‐Olsen, F. , Oh, J. S. , Buys, E. , de Jonge, B. , Shah, D. A. , Kiviniemi, T. , Burridge, P. W. , Bezzina, C. R. , Akhmanova, A. , & MacRae, C. A. (2024). The role of MAPRE2 and microtubules in maintaining normal ventricular conduction. Circulation Research, 134(1), 46–59. 38095085 10.1161/CIRCRESAHA.123.323231 PMC11889334

[tjp16866-bib-0015] Chopra, S. S. , Watanabe, H. , Zhong, T. P. , & Roden, D. M. (2007). Molecular cloning and analysis of zebrafish voltage‐gated sodium channel beta subunit genes: Implications for the evolution of electrical signaling in vertebrates. BioMed Central Evolutionary Biology, 7(113), 1–24. 17623065 10.1186/1471-2148-7-113 PMC1971062

[tjp16866-bib-0016] Crowcombe, J. , Dhillon, S. S. , Hurst, R. M. , Egginton, S. , Müller, F. , Sík, A. , & Tarte, E. (2016). 3D finite element electrical model of larval zebrafish ECG signals. PLoS ONE, 11(11), e0165655. 27824910 10.1371/journal.pone.0165655 PMC5100939

[tjp16866-bib-0017] Crumb, W. J. , Vicente, J. , Johannesen, L. , & Strauss, D. G. (2016). An evaluation of 30 clinical drugs against the comprehensive in vitro proarrhythmia assay (CiPA) proposed ion channel panel. Journal of Pharmacological and Toxicological Methods, 81, 251–262. 27060526 10.1016/j.vascn.2016.03.009

[tjp16866-bib-0018] Davidson, S. M. , & Duchen, M. R. (2018). Imaging mitochondrial calcium fluxes with fluorescent probes and single‐ or two‐photon confocal microscopy. Methods in Molecular Biology, 1782, 171–186. 29851000 10.1007/978-1-4939-7831-1_10

[tjp16866-bib-0019] De la Cruz, A. , Wu, X. , Rainer, Q. C. , Hiniesto‐Iñigo, I. , Perez, M. E. , Edler, I. , Liin, S. I. , & Larsson, H. P. (2023). Pharmacological screening of Kv7.1 and Kv7.1/KCNE1 activators as potential antiarrhythmic drugs in the zebrafish heart. International Journal of Molecular Sciences, 24(12092), 1–22. 10.3390/ijms241512092 PMC10418701 37569465

[tjp16866-bib-0020] Demir, S. S. , Clark, J. W. , Murphey, C. R. , & Giles, W. R. (1994). A mathematical model of a rabbit sinoatrial node cell. American Journal of Physiology, 266, C832–C852. 8166247 10.1152/ajpcell.1994.266.3.C832

[tjp16866-bib-0021] Genge, C. E. , Muralidharan, P. , Kemp, J. , Hull, C. M. , Yip, M. , Simpson, K. , Hunter, D. V. , & Claydon, T. W. (2024). Zebrafish cardiac repolarization does not functionally depend on the expression of the hERG1b‐like transcript. Pflugers Archiv: European Journal of Physiology, 476(1), 87–99. 37934265 10.1007/s00424-023-02875-z

[tjp16866-bib-0022] González‐Rosa, J. M. (2022). Zebrafish models of cardiac disease: From fortuitous mutants to precision medicine. Circulation Research, 130(12), 1803–1826. 35679360 10.1161/CIRCRESAHA.122.320396

[tjp16866-bib-0023] Gut, P. , Reischauer, S. , Stainier, D. Y. R. , & Arnaout, R. (2017). Little fish, big data: Zebrafish as a model for cardiovascular and metabolic disease. Physiological Reviews, 97(3), 889–938. 28468832 10.1152/physrev.00038.2016 PMC5817164

[tjp16866-bib-0024] Hassinen, M. , Haverinen, J. , Hardy, M. E. , Shiels, H. A. , & Vornanen, M. (2015). Inward rectifier potassium current (IK1) and Kir2 composition of the zebrafish (Danio rerio) heart. Pflugers Archiv: European Journal of Physiology, 467(12), 2437–2446. 25991088 10.1007/s00424-015-1710-8

[tjp16866-bib-0025] Hassinen, M. , Haverinen, J. , & Vornanen, M. (2015). Molecular basis and drug sensitivity of the delayed rectifier (IKr) in the fish heart. Comparative Biochemistry and Physiology. Toxicology & Pharmacology: CBP, 176–177, 44–51. 10.1016/j.cbpc.2015.07.005 26215639

[tjp16866-bib-0026] Haverinen, J. , Dzhumaniiazova, I. , Abramochkin, D. V. , Hassinen, M. , & Vornanen, M. (2021). Effects of Na^+^ channel isoforms and cellular environment on temperature tolerance of cardiac Na^+^ current in zebrafish (Danio rerio) and rainbow trout (Oncorhynchus mykiss). Journal of Experimental Biology, 224(8), jeb241067. 33914031 10.1242/jeb.241067

[tjp16866-bib-0027] Haverinen, J. , Hassinen, M. , Dash, S. N. , & Vornanen, M. (2018). Expression of calcium channel transcripts in the zebrafish heart: Dominance of T‐type channels. Journal of Experimental Biology, 221(10), jeb179226. 29739832 10.1242/jeb.179226

[tjp16866-bib-0028] Haverinen, J. , Hassinen, M. , Korajoki, H. , & Vornanen, M. (2018). Cardiac voltage‐gated sodium channel expression and electrophysiological characterization of the sodium current in the zebrafish (Danio rerio) ventricle. Progress in Biophysics and Molecular Biology, 138, 59–68. 29655910 10.1016/j.pbiomolbio.2018.04.002

[tjp16866-bib-0029] Hove‐Madsen, L. , Prat‐Vidal, C. , Llach, A. , Ciruela, F. , Casadó, V. , Lluis, C. , Bayes‐Genis, A. , Cinca, J. , & Franco, R. (2006). Adenosine A2A receptors are expressed in human atrial myocytes and modulate spontaneous sarcoplasmic reticulum calcium release. Cardiovascular Research, 72(2), 292–302. 17014834 10.1016/j.cardiores.2006.07.020

[tjp16866-bib-0030] Howe, K. , Clark, M. D. , Torroja, C. F. , Torrance, J. , Berthelot, C. , Muffato, M. , Collins, J. E. , Humphray, S. , McLaren, K. , Matthews, L. , McLaren, S. , Sealy, I. , Caccamo, M. , Churcher, C. , Scott, C. , Barrett, J. C. , Koch, R. , Rauch, G. J. , White, S. , … Stemple, D. L. (2013). The zebrafish reference genome sequence and its relationship to the human genome. Nature, 496(7446), 498–503. 23594743 10.1038/nature12111 PMC3703927

[tjp16866-bib-0031] Hu, N. , Yost, H. J. , & Clark, E. B. (2001). Cardiac morphology and blood pressure in the adult zebrafish. Anatomical Record, 264(1), 1–12. 11505366 10.1002/ar.1111

[tjp16866-bib-0032] Kikuchi, K. , Holdway, J. E. , Werdich, A. A. , Anderson, R. M. , Fang, Y. , Egnaczyk, G. F. , Evans, T. , MacRae, C. A. , Stainier, D. Y. R. , & Poss, K. D. (2010). Primary contribution to zebrafish heart regeneration by gata4(^+^) cardiomyocytes. Nature, 464(7288), 601–605. 20336144 10.1038/nature08804 PMC3040215

[tjp16866-bib-0033] Kompella, S. N. , Brette, F. , Hancox, J. C. , & Shiels, H. A. (2021). Phenanthrene impacts zebrafish cardiomyocyte excitability by inhibiting IKr and shortening action potential duration. Journal of General Physiology, 153(2), e202012733. 33475719 10.1085/jgp.202012733 PMC7829948

[tjp16866-bib-0034] Koopman, C. D. , De Angelis, J. , Iyer, S. P. , Verkerk, A. O. , Da Silva, J. , Berecki, G. , Jeanes, A. , Baillie, G. J. , Paterso, S. , Uribe, V. , Ehrlich, O. V. , Robinson, S. D. , Garric, L. , Petrou, S. , Simons, C. , Vetter, I. , Hogan, B. M. , de Boer, T. P. , Bakkers, J. , & Smith, K. A. (2021). The zebrafish grime mutant uncovers an evolutionarily conserved role for Tmem161b in the control of cardiac rhythm. Proceedings of the National Academy of Sciences of the United States of America, 118(9), e2018220118. 33597309 10.1073/pnas.2018220118 PMC7936323

[tjp16866-bib-0035] Kopton, R. A. , Baillie, J. S. , Rafferty, S. A. , Moss, R. , Zgierski‐Johnston, C. M. , Prykhozhij, S. V. , Stoyek, M. R. , Smith, F. M. , Kohl, P. , Quinn, T. A. , & Schneider‐Warme, F. (2018). Cardiac electrophysiological effects of light‐activated chloride channels. Frontiers in Physiology, 9(1806), 1–16. 30618818 10.3389/fphys.2018.01806 PMC6304430

[tjp16866-bib-0037] Lin, E. , Craig, C. , Lamothe, M. , Sarunic, M. V. , Beg, M. F. , & Tibbits, G. F. (2015). Construction and use of a zebrafish heart voltage and calcium optical mapping system, with integrated electrocardiogram and programmable electrical stimulation. American Journal of Physiology‐Regulatory, Integrative and Comparative Physiology, 308(9), R755–R768. 25740339 10.1152/ajpregu.00001.2015 PMC4421745

[tjp16866-bib-0038] MacDonald, E. A. , Stoyek, M. R. , Rose, R. A. , & Quinn, T. A. (2017). Intrinsic regulation of sinoatrial node function and the zebrafish as a model of stretch effects on pacemaking. Progress in Biophysics and Molecular Biology, 130(B), 198–211. 28743586 10.1016/j.pbiomolbio.2017.07.012

[tjp16866-bib-0039] Nemtsas, P. , Wettwer, E. , Christ, T. , Weidinger, G. , & Ravens, U. (2010). Adult zebrafish heart as a model for human heart? An electrophysiological study. Journal of Molecular and Cellular Cardiology, 48(1), 161–171. 19747484 10.1016/j.yjmcc.2009.08.034

[tjp16866-bib-0040] O'Hara, T. , Virág, L. , Varró, A. , & Rudy, Y. (2011). Simulation of the undiseased human cardiac ventricular action potential: Model formulation and experimental validation. PLoS Computational Biology, 7(5), e1002061. 21637795 10.1371/journal.pcbi.1002061 PMC3102752

[tjp16866-bib-0042] van Opbergen, C. J. M. , van der Voorn, S. M. , Vos, M. A. , de Boer, T. P. , & van Veen, T. A. B. (2018). Cardiac Ca^2+^ signalling in zebrafish: Translation of findings to man. Progress in Biophysics and Molecular Biology, 138, 45–58. 29746887 10.1016/j.pbiomolbio.2018.05.002

[tjp16866-bib-0043] Pozzan, T. , & Rudolf, R. (2008). Measurements of mitochondrial calcium in vivo. Biochimica Et Biophysica Acta, 1787(11), 1317–1323. 19100709 10.1016/j.bbabio.2008.11.012

[tjp16866-bib-0044] Qian, S. , & Tarte, E. (2019). Finite element modelling of discontinuous action potential propagation in larval zebrafish and human cardiac tissue. Physical Biology, 17(1), 1–14. 10.1088/1478-3975/ab4d62 31610528

[tjp16866-bib-0045] Quinn, T. A. , & Kohl, P. (2013). Combining wet and dry research: Experience with model development for cardiac mechano‐electric structure‐function studies. Cardiovascular Research, 97(4), 601–611. 23334215 10.1093/cvr/cvt003 PMC3583260

[tjp16866-bib-0046] Rafferty, S. A. , & Quinn, T. A. (2018). A beginner's guide to understanding and implementing the genetic modification of zebrafish. Progress in Biophysics and Molecular Biology, 138, 3–19. 30032905 10.1016/j.pbiomolbio.2018.07.005

[tjp16866-bib-0047] Ravens, U. (2018). Ionic basis of cardiac electrophysiology in zebrafish compared to human hearts. Progress in Biophysics and Molecular Biology, 138, 38–44. 29913181 10.1016/j.pbiomolbio.2018.06.008

[tjp16866-bib-0048] Rayani, K. , Lin, E. , Craig, C. , Lamothe, M. , Shafaattalab, S. , Gunawan, M. , Li, A. Y. , Hove‐Madsen, L. , & Tibbits, G. F. (2018). Zebrafish as a model of mammalian cardiac function: Optically mapping the interplay of temperature and rate on voltage and calcium dynamics. Progress in Biophysics and Molecular Biology, 138, 69–90. 30017908 10.1016/j.pbiomolbio.2018.07.006

[tjp16866-bib-0049] Romero, L. , Pueyo, E. , Fink, M. , & Rodríguez, B. (2009). Impact of ionic current variability on human ventricular cellular electrophysiology. American Journal of Physiology‐Heart and Circulatory Physiology, 297(4), H1436–H1445. 19648254 10.1152/ajpheart.00263.2009

[tjp16866-bib-0050] Rush, S. , & Larsen, H. (1978). A practical algorithm for solving dynamic membrane equations. Institute of Electrical and Electronics Engineers Transactions on Bio‐Medical Engineering, 25(4), 389–392. 10.1109/TBME.1978.326270 689699

[tjp16866-bib-0051] Scholz, E. P. , Niemer, N. , Hassel, D. , Zitron, E. , Bürgers, H. F. , Bloehs, R. , Seyler, C. , Scherer, D. , Thomas, D. , Kathöfer, S. , Katus, H. A. , Rottbauer, W. A. , & Karle, C. A. (2009). Biophysical properties of zebrafish ether‐à‐go‐go related gene potassium channels. Biochemical and Biophysical Research Communications, 381(2), 159–164. 19232322 10.1016/j.bbrc.2009.02.042

[tjp16866-bib-0052] Shiels, H. A. , & Galli, G. L. J. (2014). The sarcoplasmic reticulum and the evolution of the vertebrate heart. Physiology (Bethesda, Md.), 29(6), 456–469. 25362639 10.1152/physiol.00015.2014

[tjp16866-bib-0054] Stamm, C. , Friehs, I. , Choi, Y.‐H. , Zurakowski, D. , Mcgowan, F. X. , & Del Nido, P. J. (2003). Cytosolic calcium in the ischemic rabbit heart: Assessment by pH‐and temperature‐adjusted rhod‐2 spectrofluorometry. Cardiovascular Research, 59(3), 695–704. 14499871 10.1016/s0008-6363(03)00467-x

[tjp16866-bib-0055] Stoyek, M. R. , Doane, S. E. , Dallaire, S. E. , Long, Z. D. , Ramia, J. M. , Cassidy‐Nolan, D. L. , Poon, K.‐L. , Brand, T. , & Quinn, T. A. (2024). POPDC1 variants cause atrioventricular node dysfunction and arrhythmogenic changes in cardiac electrophysiology and intracellular calcium handling in zebrafish. Genes, 15(280), 1–23. 10.3390/genes15030280 PMC10969970 38540339

[tjp16866-bib-0056] Stoyek, M. R. , MacDonald, E. A. , Mantifel, M. , Baillie, J. S. , Selig, B. M. , Croll, R. P. , Smith, F. M. , & Quinn, T. A. (2022). Drivers of sinoatrial node automaticity in zebrafish: Comparison with mechanisms of mammalian pacemaker function. Frontiers in Physiology, 13(818122), 1–19. 10.3389/fphys.2022.818122 PMC8919049 35295582

[tjp16866-bib-0057] Stoyek, M. R. , & Quinn, T. A. (2018). One fish, two fish, red fish, blue fish*: Zebrafish as a model for cardiac research. Progress in Biophysics and Molecular Biology, 138, 1–2. 30514520 10.1016/j.pbiomolbio.2018.11.003

[tjp16866-bib-0059] Tomek, J. , Bueno‐Orovio, A. , Passini, E. , Zhou, X. , Minchole, A. , Britton, O. , Bartolucci, C. , Severi, S. , Shrier, A. , Virag, L. , Varro, A. , & Rodriguez, B. (2019). Development, calibration, and validation of a novel human ventricular myocyte model in health, disease, and drug block. eLife, 8, e48890. 31868580 10.7554/eLife.48890 PMC6970534

[tjp16866-bib-0060] Trayanova, N. A. , Lyon, A. , Shade, J. , & Heijman, J. (2024). Computational modeling of cardiac electrophysiology and arrhythmogenesis: Toward clinical translation. Physiological Reviews, 104(3), 1265–1333. 38153307 10.1152/physrev.00017.2023 PMC11381036

[tjp16866-bib-0061] Tsai, C.‐T. , Wu, C.‐K. , Chiang, F.‐T. , Tseng, C.‐D. , Lee, J.‐K. , Yu, C.‐C. , Wang, Y.‐C. , Lai, L.‐P. , Lin, J.‐L. , & Hwang, J.‐J. (2011). In‐vitro recording of adult zebrafish heart electrocardiogram‐A platform for pharmacological testing. Clinica Chimica Acta, 412(21–22), 1963–1967. 10.1016/j.cca.2011.07.002 21767531

[tjp16866-bib-0062] ten Tusscher, K. H. W. J. , Noble, D. , Noble, P. J. , & Panfilov, A. V. (2004). A model for human ventricular tissue. American Journal of Physiology‐Heart and Circulatory Physiology, 286(4), H1573–H1589. 14656705 10.1152/ajpheart.00794.2003

[tjp16866-bib-0063] ten Tusscher, K. H. W. J. , & Panfilov, A. V. (2006). Alternans and spiral breakup in a human ventricular tissue model. American Journal of Physiology‐Heart and Circulatory Physiology, 291(3), H1088–H1100. 16565318 10.1152/ajpheart.00109.2006

[tjp16866-bib-0064] Varró, A. , Tomek, J. , Nagy, N. , Virág, L. , Passini, E. , Rodriguez, B. , & Baczkó, I. (2021). Cardiac transmembrane ion channels and action potentials: Cellular physiology and arrhythmogenic behavior. Physiological Reviews, 101(3), 1083–1176. 33118864 10.1152/physrev.00024.2019

[tjp16866-bib-0065] Vornanen, M. , & Hassinen, M. (2016). Zebrafish heart as a model for human cardiac electrophysiology. Channels, 10(2), 101–110. 26671745 10.1080/19336950.2015.1121335 PMC4960994

[tjp16866-bib-0066] Wang, J. , Panáková, D. , Kikuchi, K. , Holdway, J. E. , Gemberling, M. , Burris, J. S. , Singh, S. P. , Dickson, A. L. , Lin, Y. F. , Khaled Sabeh, M. , Werdich, A. A. , Yelon, D. , MacRae, C. A. , & Poss, K. D. (2011). The regenerative capacity of zebrafish reverses cardiac failure caused by genetic cardiomyocyte depletion. Development (Cambridge, England), 138(16), 3421–3430. 21752928 10.1242/dev.068601 PMC3143562

[tjp16866-bib-0067] Yue, L. , Feng, J. , Li, G. R. , & Nattel, S. (1996). Transient outward and delayed rectifier currents in canine atrium: Properties and role of isolation methods. The American journal of physiology, 270(6), H2157–H2168. 8764269 10.1152/ajpheart.1996.270.6.H2157

[tjp16866-bib-0068] Zhang, H. , Holden, A. V. , Kodama, I. , Honjo, H. , Lei, M. , Varghese, T. , & Boyett, M. R. (2000). Mathematical models of action potentials in the periphery and center of the rabbit sinoatrial node. American Journal of Physiology‐Heart and Circulatory Physiology, 279(1), H397–H421. 10899081 10.1152/ajpheart.2000.279.1.H397

[tjp16866-bib-0069] Zhang, P. C. , Llach, A. , Sheng, X. Y. , Hove‐Madsen, L. , & Tibbits, G. F. (2011). Calcium handling in zebrafish ventricular myocytes. American Journal of Physiology‐Regulatory, Integrative and Comparative Physiology, 300(1), R56–R66. 20926764 10.1152/ajpregu.00377.2010

