# Supplementary material for: Mathematical model of the zebrafish ventricular cardiomyocyte action potential and calcium transient
Source: J Physiol. 2025 Jul 31;604(13):5391–420. doi: 10.1113/JP287624 (PMC13327776; doi:10.1113/JP287624)
Supplement: Supplementary file 1 — Peer Review History [file TJP-604-5391-s001.pdf]

The Journal of Physiology JP

Mathematical Model of the Zebrafish Ventricular Cardiomyocyte Action Potential and Calcium Transient

Ludovica Cestariolo, Zachary David Long, Arie O Verkerk, Jose Maria Ferrero, T Alexander Quinn, and Jose Felix Rodriguez Matas
DOI: 10.1113/JP287624

Corresponding author(s): Ludovica Cestariolo (ludovica.cestariolo@polimi.it)

The following individual(s) involved in review of this submission have agreed to reveal their identity: Ronald Wilders (Referee #2)

Review Timeline:

Submission Date:	08-Oct-2024
Editorial Decision:	02-Dec-2024
Revision Received:	06-Mar-2025
Editorial Decision:	31-Mar-2025
Revision Received:	28-Apr-2025
Editorial Decision:	23-May-2025
Revision Received:	20-Jun-2025
Accepted:	27-Jun-2025

Senior Editor: Bjorn Knollmann

Reviewing Editor: Axel Loewe

Transaction Report:

(Note: With the exception of the correction of typographical or spelling errors that could be a source of ambiguity, letters and reports are not edited. Depending on transfer agreements, referee reports obtained elsewhere may or may not be included in this compilation. Referee reports are anonymous unless the Referee chooses to sign their reports.)

Dear Dr Cestariolo,

Re: JP-RP-2024-287624 "A mathematical model of the zebrafish ventricular action potential from whole-heart voltage-calcium data" by Ludovica Cestariolo, Jose Maria Ferrero, Zachary David Long, T Alexander Quinn, and Jose Felix Rodriguez Matas

Thank you for submitting your manuscript to The Journal of Physiology. It has been assessed by a Reviewing Editor and by 2 expert referees and we are pleased to tell you that it is potentially acceptable for publication following satisfactory major revision.

The referee reports are copied at the end of this email.

Please address all the points raised and incorporate all requested revisions or explain in your Response to Referees why a change has not been made. We hope you will find the comments helpful and that you will be able to return your revised manuscript within 9 months. If you require longer than this, please contact journal staff: jp@physoc.org. Please note that this letter does not constitute a guarantee for acceptance of your revised manuscript.

Your revised manuscript should be submitted online using the link in your Author Tasks: Link Not Available. This link is accessible via your account as Corresponding Author; it is not available to your co-authors. If this presents a problem, please contact journal staff (jp@physoc.org). Image files from the previous version are retained on the system. Please ensure you replace or remove any files that are being revised.

If you do not wish to submit a revised version of your manuscript, you must inform our journal staff (jp@physoc.org) or reply to this email to request withdrawal. Please note that a manuscript must be formally withdrawn from the peer review process at one journal before it may be submitted to another journal.

TRANSPARENT PEER REVIEW POLICY: To improve the transparency of its peer review process, The Journal of Physiology publishes online as supporting information the peer review history of all articles accepted for publication. Readers will have access to decision letters, including Editors' comments and referee reports, for each version of the manuscript, as well as any author responses to peer review comments. Referees can decide whether or not they wish to be named on the peer review history document.

ABSTRACT FIGURES: Authors are expected to use The Journal's premium BioRender account to create/redraw their Abstract Figures. Information on how to access this account is here: https://physoc.onlinelibrary.wiley.com/journal/14697793/biorender-access.

This will enable Authors to create and download high-resolution figures. If authors have used the free BioRender service, they can use the instructions provided in the link above to download a high-resolution version suitable for publication.

The link provided should only be used for the purposes of this submission. Authors will be charged for figures created on this account if they are not related to this manuscript submission.

LANGUAGE EDITING AND SUPPORT FOR PUBLICATION: If you would like help with English language editing, or other article preparation support, Wiley Editing Services offers expert help, including English Language Editing, as well as translation, manuscript formatting, and figure formatting at www.wileyauthors.com/eeo/preparation. You can also find resources for Preparing Your Article for general guidance about writing and preparing your manuscript at www.wileyauthors.com/eeo/prepresources.

RIGOUR AND REPRODUCIBILITY: We recommend authors consult The Journal's Rigour and Reproducibility requirements to ensure that their manuscript is suitable for publication: https://physoc.onlinelibrary.wiley.com/pb-assets/hub-assets/physoc/documents/TJP-Rigour-and-Reproducibility-Requirements.pdf

REVISION CHECKLIST:

Check that your Methods section conforms to journal policy: https://jp.msubmit.net/cgi-bin/main.plex?form_type=display_requirements#methods.

Check that data presented conforms to the statistics policy: https://jp.msubmit.net/cgi-bin/main.plex?form_type=display_requirements#statistics.

Upload a full Response to Referees file. To create your 'Response to Referees': copy all the reports, including any comments from the Senior and Reviewing Editors, into a Microsoft Word, or similar, file and respond to each point, using font or background colour to distinguish comments and responses and upload as the required file type.

Please upload two versions of your manuscript text: one with all relevant changes highlighted and one clean version with no changes tracked. The manuscript file should include all tables and figure legends, but each figure/graph should be uploaded

as separate, high-resolution files.

You may also upload:

- 'Potential Cover Art' for consideration as the issue's cover image
- Appropriate Supporting Information (Video, audio or data set: see https://jp.msubmit.net/cgi-bin/main.plex?form_type=display_requirements#supp).

We look forward to receiving your revised submission.

If you have any queries, please reply to this email and we will be pleased to advise.

Yours sincerely,

Bjorn Knollmann
Senior Editor
The Journal of Physiology

REQUIRED ITEMS

- Author photo and profile. First or joint first authors are asked to provide a short biography (no more than 100 words for one author or 150 words in total for joint first authors) and a portrait photograph. These should be uploaded and clearly labelled together in a Word document with the revised version of the manuscript. See Information for Authors for further details.

- You must start the Methods section with a paragraph headed Ethical approval (https://jp.msubmit.net/cgi-bin/main.plex?form_type=display_requirements#methods).

Research must comply with The Journal's policies regarding animal experiments (https://physoc.onlinelibrary.wiley.com/hub/animal-experiments) and adherence to these policies must be stated in the manuscript.

Authors should confirm in their Methods section that their experiments were carried out according to the guidelines laid down by their institution's animal welfare committee, including an ethics approval reference number. The Methods section must contain a statement about access to food, water and housing, details of the anaesthetic regime: anaesthetic used, dose and route of administration, and method of killing the experimental animals.

- Please upload separate high-quality figure files via the submission form.

- Please ensure that any tables are editable and in Word format, and wherever possible, embedded in the article file itself.

- Your paper contains Supporting Information of a type that we no longer publish, including supplementary tables and figures. Any information essential to an understanding of the paper must be included as part of the main manuscript and figures. The only Supporting Information that we publish are video and audio, 3D structures, program codes and large data files. Your revised paper will be returned to you if it does not adhere to our Supporting Information Guidelines.

- Papers must comply with the Statistics Policy: https://jp.msubmit.net/cgi-bin/main.plex?form_type=display_requirements#statistics.

In summary:

- If n {less than or equal to} 30, all data points must be plotted in the figure in a way that reveals their range and distribution. A bar graph with data points overlaid, a box and whisker plot or a violin plot (preferably with data points included) are acceptable formats.

- If n > 30, then the entire raw dataset must be made available either as supporting information, or hosted on a not-for-profit repository, e.g. FigShare, with access details provided in the manuscript.

- 'n' clearly defined (e.g. x cells from y slices in z animals) in the Methods. Authors should be mindful of pseudoreplication.

- All relevant 'n' values must be clearly stated in the main text, figures and tables.

- The most appropriate summary statistic (e.g. mean or median and standard deviation) must be used. Standard Error of the Mean (SEM) alone is not permitted.

- Exact p values must be stated. Authors must not use 'greater than' or 'less than'. Exact p values must be stated to three significant figures even when 'no statistical significance' is claimed.

- Please include an Abstract Figure file, as well as the Figure Legend text within the main article file. The Abstract Figure is a piece of artwork designed to give readers an immediate understanding of the research and should summarise the main conclusions. If possible, the image should be easily 'readable' from left to right or top to bottom. It should show the physiological relevance of the manuscript so readers can assess the importance and content of its findings. Abstract Figures should not merely recapitulate other figures in the manuscript. Please try to keep the diagram as simple as possible and without superfluous information that may distract from the main conclusion(s). Abstract Figures must be provided by authors no later than the revised manuscript stage and should be uploaded as a separate file during online submission labelled as File Type 'Abstract Figure'. Please also ensure that you include the figure legend in the main article file. All Abstract Figures should be created using BioRender. Authors should use The Journal's premium BioRender account to export high-resolution images. Details on how to use and access the premium account are included as part of this email.

EDITOR COMMENTS

Reviewing Editor: Methods Details:
Authors should publish the model in CellML format in the CellML repository and all computational code as supplementary material

Comments for Authors to ensure the paper complies with the Statistics Policy (Required):
Some experimental data are only reported as mean (e.g. figure 2 and following). If in some cases this is because literature data were used and only mean values were reported there, this should be clearly stated.

Comments to the Author (Required):
The two expert reviewers had different views of the paper. Reviewer #1 highlighted the contributions presented in the manuscript and pointed out details to be addressed.

Both referees point out that the decision for the (rather old) parent model needs to be better motivated.

Referee #2 mentions a number of key issues that should be addressed to make this manuscript suitable for further consideration.

Importantly, model definition and simulation code need to be made available according to the Journal's guidelines.

Writing should be improved focussing on clear, concise and unambiguous formulations (e.g. key points 2 and 3 of referee #2).

Please also see 'Required Items' above.

Senior Editor:

Comments for Authors to ensure the paper complies with the Statistics Policy (Required):
Please adhere to journal statistics policy

Comments to the Author:

Both reviewers and reviewing editor raise substantial concerns that would require extensive rewriting before further consideration. I concur.

REFEREE COMMENTS

Referee #1:

Review JP-RP-2024-287624

Reflecting on the manuscript by Cestariolo et al., I think this paper is a great contribution to readers interested in using the zebrafish as a model for cardiac electrophysiology. This reviewer has done himself some late-night experiments trying to do what the authors did, with much less success.

In their work, the authors have adapted a leading mathematical model of the human ventricular action potential to reproduce to a large extent the action potentials observed in adult zebrafish ventricle. The approach taken is valid, and the results from the model compare well with the experimental data presented in the manuscript, and other published zebrafish action potential data. I would like to encourage the authors to publish the full Matlab implementation of the model code for re-use by others.

Minor points

Line 77: Zebrafish do not have a similar physiology and genetics to human. There are useful similarities off course, but this is overstating. They miss half a heart and have complicated gene duplications that can make genetics comparisons difficult.

Line 342: 'zebrafish activation channels' probably refers to activation gates?

About calcium handling: there are some more details about calcium handling in zebrafish cardiomyocytes in literature, e.g. Bovo et al. 2013. It would be good to give some more attention to that, and show how the model behaves:

- What is the fractional SR calcium release?

- Which contributes most to calcium influx, LTCC/TTCC, NCX or RyR?

- How do the absolute calcium levels of the model compare with experimental findings of the authors / in literature?

Line 383: how much bigger is GCaL in zebrafish compared to human?

Line 480: There are indications in literature that the Na+-Ca2+ exchanger current is much larger in zebrafish, reviewed in Van Opbergen et al., PMID: 29746887

Line 535: Please clarify if the number of recordings means number of hearts or impalements.

Referee #2:

General

The authors have constructed a mathematical model for the electrophysiological behaviour of an isolated zebrafish ventricular myocyte. Unfortunately, as detailed in my specific comments below, the authors have started from an over 20-year-old human ventricular cell model (their "parent model") because of its "high computational cost-effectiveness and justified by the similarities between humans and zebrafish". However, in doing so, they should have carefully checked the availability of experimental data obtained in (adult) zebrafish ventricular myocytes and adapted their model equations based on these data. This is not the case. For example, the zebrafish sodium current is now based on experimental data from a single study in an expression system at room temperature, whereas there are experimental data from several studies on isolated adult zebrafish ventricular myocytes, even at physiological temperature.

Specific

- Title

What are "whole-heart voltage-calcium data"? Are they voltage clamp and calcium transient data? And were they obtained at the whole heart level? The title should be reworded.

- Key Points

1) "We have developed the first zebrafish-specific biophysical computational model of the ventricular action potential". It appears that you published an earlier version of the model in 2022 (DOI: 10.22489/CinC.2022.221), although at that time it was based on the Ten Tusscher and Panfilov (2006) human ventricular myocyte model rather than the Ten Tusscher et al. (2004) model. Furthermore, you published another version of the model in 2023 (DOI: 10.22489/CinC.2023.323), also based on the Ten Tusscher et al. (2004) model. You should at least refer to the 2022 and 2023 models and discuss any differences from the current model.

2) If "The model identified L-type calcium current (ICaL) as a key current for the zebrafish action potential", what are the key currents other than ICaL? Or is ICaL the sole key current rather than "a key current"? Please clarify in the text.

3) What exactly is meant by "the model suggested an IKs to IKr delayed rectifier potassium current ratio of ~1:12"? Does this refer to the current amplitude during an action potential (at a given rate) or is this the conductance? However, in the latter case, it is simply what you put into the model rather than what the model "suggested". Please clarify this in the text.

- Missing computation codes and detailed sensitivity analysis

I checked the "Information for Authors" of The Journal of Physiology and found out that it contains "Extra information for modelling papers". This extra information reads: "As in any experimental study, results from the model should add new scientific insights. The model should be based on physiological data where the parameters can be directly linked to experimental data. The model should be described in such a way that the results can be reproduced, i.e. all parameters are listed and any computation codes made available. Where possible there should be a detailed sensitivity analysis of the conclusions reached against the parameters proposed within the bounds of the data used to constrain them. Papers that develop integrative physiological models which link functions together or observations across scales would be encouraged." I am not sure whether the statement that "the Matlab simulation scripts are available upon request" is sufficient to meet the requirement that "any computation codes [are] made available". However, the manuscript is not accompanied by the required "computation codes". You should provide these computation codes, so that apparent errors in the listed equations (see below) can be checked against the model codes.

- Abstract

1) Is it possible to be more specific than "published data from zebrafish"? Are these single cell and/or whole heart current and/or voltage clamp experimental data?

2) Is it possible to be more specific than "a broad set of new experimental data, including measurements of action potential and calcium transient duration from steady-state and restitution pacing protocols"? Are these patch clamp and optical mapping data obtained at the single cell level, as I would expect when constructing a single cell model? The list of keywords below the abstract suggests that the experimental data were obtained using microelectrode recordings rather than patch clamp recordings. Is this correct?

3) What are the criteria for the model to be "sufficiently computationally efficient"? I could not find these anywhere in the manuscript.

- Introduction

1) Line 92: It is not very common to write "wet-dry investigations" when referring to a combination of in vitro and in silico experiments, which you probably do. Consider rewording.

2) Lines 99-102: "For this, the Ten Tusscher and Panfilov formulation of the human ventricular AP (TP04) (Ten Tusscher et al., 2004) - chosen for its high computational cost-effectiveness and justified by the similarities between humans and zebrafish - was selected as a parent model." This 'parent model' was developed over 20 years ago, based on the experimental data that were available at the time. Since then, several human ventricular cell models have been developed that are based on more recent experimental data. It may well be that these models contain descriptions of individual ionic currents that are almost as computationally inexpensive, but are much more accurate. Have you checked these newer models for such improved ionic current formulations? What was the outcome? These more recent models do include not only the model by O'Hara et al. (2011), which is listed in your references, but also the models by Tomek et al. (2019; DOI: 10.7554/eLife.48890) and Bartolucci et al. (2020, DOI: 10.3389/fphys.2020.00314), which are not.

- Methods

1) Line 159: "Intracellular microelectrode recordings of V were performed as previously described (Stoyek et al., 2024)." This is the first appearance of "V". What does it denote?

2) Line 178: "RMP (stable Vm during diastole)" This is the first appearance of "Vm". Apparently , it denotes membrane potential. Is this correct? What is the difference between V and Vm?

3) Line 187: "the excitation-contraction uncoupler {plus minus}blebbistatin". Apparently, you mean (+/–)-blebbistatin. Write it as such.

4) Line 190: "10 µM Rhod-2 AM (ab1427870, abcam, Cambridge, UK; Kd≈2.30 µM)". This is the first appearance of "Kd". What does it denote? The name of the company is Abcam (see their website), although their logo shows "abcam".

5) Line 191: "5 µM Fluo-5F AM (20560, AAT Bioquest, Pleasanton, USA; ; Kd≈0.57 µM)". Is something missing between the two semicolons?

6) Figure 1: "Tropin" and "Citosol" should probably read "Troponin" and "Cytosol".

7) Legend to Figure 1: "Schematic representation of the adult zebrafish ventricular cardiomyocyte." This legend needs an explanation of all the abbreviations shown and the role of "Calsequestrin", "Calmodulin" and "Tropin" play in the model.

8) Lines 275-276: "The model was implemented in Matlab. For the 0D simulations, a stiff solver with an adaptive time step was used." Which version of MATLAB? Which "stiff solver"?

9) Lines 260-261: "m, h, and j for INa, dT and fT for ICaT, dL, fL, and fCa for ICaL, xs for IKs, and xr1 and xr2 for IKr". This is the first appearance of these abbreviations. They should either be explained here or the reader should be referred to the section where the explanation can readily be found.

10) Lines 266-268: "The effective tissue conductivity was assumed to be σ = 3.012 · 10–4 mS, which results in a conduction velocity of 1.27 cm/s, which is consistent with our measurements (1.25 cm/s) and data reported in the literature (Panáková et al., 2010)." Was this value of σ chosen because it yields a conduction velocity near 1.25 cm/s? What are the "data reported in the literature"? Are these "data reported in the literature" data on conductivity or on conduction velocity?

11) Lines 284-286: "To calibrate model parameters, an initial Montecarlo simulation was performed to identify the most suitable model, which was then followed by a manual tuning of the parameters." This is a rather vague description. Given the aforementioned requirement that "the model should be described in such a way that the results can be reproduced", a more detailed description should be provided. Also, write "Monte Carlo" (as in the next section) instead of "Montecarlo".

12) Lines 291-293: "Monte Carlo method was used with 5000 combinations of 17 parameters representing the maximum conductance of major currents and determinants of Ca2+ dynamics (i.e., GNa, GCaL, GCaT, GKr, GKs, GK1, GpCa, GbNa, GbCa, GNaK, GNaCa, among others)." What are these six "others" among the 17 model parameters? Why was this set of parameters chosen?

13) Lines 301-302: "for the block of IKr with chromanol 293B, IKs with E4031". Chromanol 293B blocks IKs, whereas E4031

blocks IKr. Please correct.

14) Lines 319-320: "For most of the currents, the same formulation proposed in the original TP04 human ventricular AP model was utilised." This is most unfortunate because, as stated above, the "TP04 human ventricular AP model" was developed over 20 years ago, based on the experimental data that were available at that time.

15) Lines 326-328: "The voltage-gated fast Na+ channels play a crucial role in the rapid upstroke of the ventricular cardiomyocyte AP (Phase 0), and they are partly responsible for determining the velocity of impulse transmission between cells (Vornanen & Hassinen, 2016)." This is one of those (there are more) annoying references in the manuscript. Annoying, in this particular case, because Vornanen & Hassinen (2016) simply make a similar statement, referring to two papers that, when checked, appear to be irrelevant to AP conduction velocity. Authors should provide references to relevant papers that they themselves have checked, and avoid reference after reference that ends up in irrelevant papers.

16) Lines 341-343: "using the steady-state of zebrafish activation channels expressed in Chinese Hamster Ovary (CHO) cells as reported in Chopra et al., 2007." I have several comments. First, it would be helpful to write "as reported in Chopra et al., 2007 (their Figure 6 and Table 6)", so that your readers know which data you are referring to. Second, and more importantly, you should inform your readers how you have dealt with differences between the experimental conditions and your model. For example, the voltage clamp experiments of Chopra et al. (2007) were performed at room temperature, whereas your model simulates a myocyte at 301 K (28{degree sign}C). How did you correct for the associated differences in sodium current kinetics? Third, and perhaps most importantly, I do not understand why you based your model on 2007 data obtained in an expression system (expressing only NaV1.5, and therefore not NaV1.4) when there are more recent data obtained in studies of isolated adult zebrafish ventricular myocytes. One such study is that of Haverinen et al. (2018). In their paper (DOI: 10.1016/j.pbiomolbio.2018.04.002), they even present experimental data on the fast time constant of inactivation, which the authors now base on the recovery from inactivation obtained with their 'parent model'. Other more recent studies with data on the sodium current in isolated adult zebrafish ventricular myocytes are those by Haverinen et al. (2021) and Chiang et al. (2024) with DOI codes of 10.1242/jeb.241067 and 10.1161/CIRCRESAHA.123.323231, respectively.

17) Lines 351-352: "the formulations for τh and τj were obtained by translating and scaling the TP04 equations". This process requires a better explanation than "by translating and scaling". How were the original equations translated and scaled? Did this process include a correction for the differences in temperature? If so, what was the Q10; and why that value?

18) Figure 2: Again, I have several comments. First, when including experimental data, you should present all values as mean {plus minus} standard error of the mean (SEM), rather than just the mean values. Second, the labels should be more specific. This applies not only to Figure 2, but also to other figures containing experimental data. The ordinate label of panel A should read something like "steady-state (in)activation" and it should be indicated which is the activation curve and which is the inactivation curve. Panels B, D and E should not all three be labelled "t [ms]", but something like "Activation time constant [ms]", "Fast inactivation time constant [ms]" and "Slow inactivation time constant [ms]". Panel C lacks an ordinate label. Its ordinate label should read something like "Normalized current". Third, the slope of the inactivation curves changes suddenly near −30 mV, which seems rather unphysiological. I understand that this is probably due to the 'parent model', but this should be further encouragement to reformulate the associated equations.

19) Lines 361-363: "Since in the adult human heart, ICaT is mainly limited to the sinoatrial node and conductive pathways (Vornanen & Hassinen, 2016)". What about the AV node?

20) Lines 369-371: "In order to fit the experimental behaviour of ICaT in zebrafish with the rabbit model formulation, data from Haverinen et al., 2018 were taken, and the experimental patch-clamp protocol was modelled to fit the current-voltage curve as shown in Figure 3". What exactly did you do "to fit the current-voltage curve"? Why did you not consider the electrophysiological data from Nemtsas et al. (2010; DOI: 10.1016/j.yjmcc.2009.08.034)?

21) Figure 3: Why is the "computational current-voltage (I-V) curve" not shown as a smooth curve? It seems to consist of straight lines that connect simulation results obtained at −80 mV, −70 mV, −60 mV, etc. And is this really the best fit that you could obtain? At −50 mV, for example, the experimental current density is >3 times larger than the "numerical" current density.

22) Lines 395-396: "experimental data from Kompella et al., 2021 and Zhang et al., 2011". There are more data available on the L-type calcium current than those obtained by Kompella et al. (2021) and Zhang et al. (2011). Just one example, resulting from a straightforward Google Scholar search, is the study by Koopman et al. (2021; DOI: 10.1073/pnas.2018220118), which provides experimental data on the current density. In that same study, experimental data on the current density of the rapid delayed rectifier K+ current inward rectifier K+ current are shown. Why were such other data not considered?

23) Figures 4-6: Why are experimental steady-state current-voltage relationships not shown? Such experimental data are available.

- Results

1) Figure 8, Table 2: Your simulation data agree well with your microelectrode recordings. However, there are many more zebrafish action potential recordings available, including patch clamp recordings from isolated myocytes at physiological temperature. How do your simulation data agree with these patch clamp data?

2) Figure 9, Table 3: Your simulated calcium transients agree well with your calcium transient recordings. However, there are many more calcium transient recordings available in the scientific literature. How do your simulation data agree with these additional data?

3) Lines 590-592: "Unfortunately, there are no available experimental recordings for these currents to allow for a direct comparison with the model". I tend to disagree. The paper by Kompella et al. (2021), which is in your reference list, shows action potential clamp recordings of the rapid delayed rectifier potassium current. There may also be other studies that show such recordings.

- Discussion

Lines 679-681: "Here we describe the development of the first computational model of the zebrafish ventricular AP, considering the most recent patch-clamp data available in the literature for parameterisation of the major ionic currents." This is not true, as explained in my comments above. The authors should have done a solid search for such "recent patch-clamp data" and would have found many more studies than the ones they refer to.

- Appendix 1

I have not checked all of the equations listed in Appendix 1 against the 'parent model' equations, which will probably be publicly available somewhere. However, I did notice an obvious error in one of the first lines of the appendix. This is in the Nernst potential for ECa, where the charge of the calcium ions is not correctly included. Given this obvious mistake, I doubt that the remaining equations listed in Appendix 1 are all correct. This is another reason why the computation codes should be made publicly available.

- Appendix 2

Similarly, I have not checked all of the parameters listed in Appendix 2 against the parameters of the 'parent model'. However, just looking at the first few lines of that appendix, I noticed that the value of the parameter σ is listed here as the computer code-like "3.012e-04 mS" (without superscript), whereas it appears as "3.012 · 10−4 mS" (to be preferred) in the body text. Given the unit of mS, it should be conductance, but given the Greek σ to denote it, it should be conductivity. This is confusing and should be checked carefully.

- References

The list of references is sloppy in numerous respects:

(a) The references are not completely in journal style. In particular, attention to punctuation is required.

(b) The alphabetical order is inconsistent. De la Cruz et al. (2023), for example, appears between Kompella et al. (2021) and Nemtsas et al. (2010). Similarly, Tsai et al. (2011) should appear after Ten Tusscher et al. (2004), not before. Van Opbergen et al. (2018) should be alphabetically ordered based on "Van" rather "Opbergen".

(c) No attention has been paid to subscripts, superscripts and italics in the titles of papers.

(d) The format of the paper titles is not consistent. Some appear in Sentence case, others in Title Case.

(e) The naming of journals is not consistent. Pflügers Archiv appears as "Pflugers Arch", but also as "Pflugers Arch Eur J Physiol".

(f) The format of author names is inconsistent. In the first references, as an example, the author "Abramochkin DV" appears as "Abramochkin D V.".

(g) Babcock et al. (1997): plus sign missing in the title.

(h) Carro et al. (2016): no journal name, volume number, page numbers.

(i) Chopra et al. (2007): no volume and page numbers.

(j) De la Cruz et al. (2023): no volume and page numbers; DOI is 10.3390/IJMS241512092, not "10.3390/IJMS241512092/S1".

(k) Demir et al. (1994): no volume and page numbers.

(l) Gut et al. (2017): incomplete page numbers.

(m) Haverinen et al. (2018): no volume and page numbers.

(n) Howe et al. (2013): incomplete page numbers.

(o) Howe et al. (2013): the journal instructions state "When the work has 21 or more authors, include only the first 19 names, an ellipsis, and the final name (with no ampersand)."

(p) O'Hara et al. (2011): the first author's name is O'Hara, not "O'hara".

(q) Panáková et al. (2010): the journal name is Nature, not "Nat 2010 4667308".

(r) Pozzan & Rudolf (2008): no journal name, volume number, page numbers.

(s) Romero et al. (2009): journal name is Am J Physiol Heart Circ Physiol, not "Am J Physiol Hear Circ Physiol".

(t) Stamm et al. (2003): title starts with Cytosolic, not "C ytosolic".

(u) Stoyek et al. (2024): no volume and page numbers.

(v) Sun et al. (1997): no journal name, volume number, page numbers.

(w) Ten Tusscher et al. (2004): no volume and page numbers.

(x) Tsai et al. (2011): no journal name, volume number, page numbers.

(y) Zhang et al. (2000): no volume and page numbers.

(z) Zhang et al. (2011): no volume and page numbers.

- Acknowledgments

"The authors thank Prof. Arie Verkerk for sharing patch-clamp inactivation data of L-type calcium channels." Where exactly in the manuscript were these "patch-clamp inactivation data" used? Under which conditions were these data obtained?

- Supporting Material, Figure S1

The legend to Figure S1, or the figure itself, should make clear what the two coloured lines are. Are they "Model 0D" and "Model 1D", as in Figure 11?

- Supporting Material, Figure S2

The legend to Figure S2 should clarify the conditions under which the calcium transients were obtained. The main manuscript only tells us that "Figure S2 in the Supporting Material shows computational results with increased Ca2+ buffering to mimic the effect of Rhod-2", without quantifying the stimulus frequency at which these transients were obtained. Apparently, it is 1 Hz, since section 2.4 of the main manuscript states that "an abbreviated pacing protocol was used to compare Ca2+ transients measurements to Rhod-2: a train of 100 stimuli was delivered to the heart, with cycle lengths of 1000, 500, and 250 ms", in relation to the duration of the transient observed in the figure.

- Supporting Material, Figure S3

The legend to Figure S3 should clarify the conditions under which [Na+]i and [K+]i were obtained. The main manuscript says: "In particular, the ability of the model to stabilize the intracellular Na+ and intracellular K+ at different stimulation frequencies was verified (see Figure S3 in the Supporting Material)." So, where are these "different stimulation frequencies" in Figure S3? Or is Figure S3 just for a single stimulation frequency? And, if so, what is this specific stimulation frequency? Why do [Na+]i and [K+]i start at around 11.5 and 109 mM, respectively? Are these the concentration values obtained under steady-state resting conditions?

END OF COMMENTS

We thank the editors and reviewers for their insightful and critical review of our manuscript; their feedback has helped us to improve the overall quality, importance, and potential impact of the study. We hope the extensive revisions, including enhanced explanations and additional analysis, address the reviewers' concerns.

EDITOR COMMENTS

Reviewing Editor:

Methods Details:
- Authors should publish the model in CellML format in the CellML repository and all computational code as supplementary material

We absolutely agree. The 0D model will be provided in CellML format in the CellML repository and the MATLAB code for both the 0D and 1D models will be available on GitHub (https://github.com/jfrodrig1971/Zebrafish) in accordance with the journal guidelines to ensure reproducibility and clarity. At the moment, the listings have been generated but remain private; they will be made public upon acceptance of the work.

Comments for Authors to ensure the paper complies with the Statistics Policy (Required):
- Some experimental data are only reported as mean (e.g., figure 2 and following). If, in some cases, this is because literature data were used and only mean values were reported there, this should be clearly stated.

We have remade all of the figures, correctly incorporating the experimental data as mean ± standard deviation where available (in the cases where mean values were only available, this is now clearly stated).

Comments to the Author (Required):
- The two expert reviewers had different views of the paper. Reviewer #1 highlighted the contributions presented in the manuscript and pointed out details to be addressed.
- Both referees point out that the decision for the (rather old) parent model needs to be better motivated.
- Referee #2 mentions a number of key issues that should be addressed to make this manuscript suitable for further consideration.
- Importantly, model definition and simulation code need to be made available according to the Journal's guidelines.
- Writing should be improved, focussing on clear, concise, and unambiguous formulations (e.g., key points 2 and 3 of referee #2).
- Please also see 'Required Items' above.

Thank you for this concise summary of the reviewers' main concerns. The important points raised by the reviewers are addressed in the point-by-point response below. The 'Required Items' of the Journal have now also been carefully addressed in our revision.

Senior Editor:

Comments for Authors to ensure the paper complies with the Statistics Policy (Required):
- Please adhere to journal statistics policy

We have remade all of the figures, correctly incorporating the experimental data as mean ± standard deviation where available (in the cases where mean values were only available, this is now clearly stated).

Comments to the Author:
- Both reviewers and reviewing editor raise substantial concerns that would require extensive rewriting before further consideration. I concur.

We appreciate the comment and agree. We have now made extensive revisions to the paper to address the important comments from the reviewers' and reviewing editor, as outlined above and in the point-by-point response below.

REFEREE COMMENTS

Referee #1:

Reflecting on the manuscript by Cestariolo et al., I think this paper is a great contribution to readers interested in using the zebrafish as a model for cardiac electrophysiology. This reviewer has done himself some late-night experiments trying to do what the authors did, with much less success.

In their work, the authors have adapted a leading mathematical model of the human ventricular action potential to reproduce to a large extent the action potentials observed in adult zebrafish ventricle. The approach taken is valid, and the results from the model compare well with the experimental data presented in the manuscript, and other published zebrafish action potential data. I would like to encourage the authors to publish the full Matlab implementation of the model code for re-use by others.

We thank the reviewer for the kind words and positive feedback on our work. We truly appreciate the thoughtful comments and are glad that they found our study valuable. The 0D model will be provided in CellML and the MATLAB code for both the 0D and 1D models will be available on GitHub (as per the journal guidelines) to ensure reproducibility and clarity. At the moment, the listings have been generated but remain private; they will be made public upon acceptance of the work.

Minor points:

1) Line 77: Zebrafish do not have a similar physiology and genetics to human. There are useful similarities off course, but this is overstating. They miss half a heart and have complicated gene duplications that can make genetics comparisons difficult.

We appreciate the reviewer's comment and agree that we overstated the similarities and neglected to expand on limitations. That said, while they do have different general physiology, their cardiac electrophysiology is quite comparable to that of human (which is the primary focus of this paper). We have revised the Introduction and Discussion to remove the overstatements and focus on highlighting not only the important advantages of the zebrafish but also the key differences compared to mammals.

Lines 106-135 now read: "*The zebrafish (Danio rerio) has emerged as a powerful experimental model for cardiac research, in part due to its remarkable similarities to human cardiac function and its high degree of genetic homology (Gut et al., 2017), with most zebrafish cardiac genes having human orthologues with analogous function (Howe et al., 2013). Zebrafish offer distinct advantages over traditional mammalian models, being more cost-effective and requiring less intensive husbandry and experimental resources. Furthermore, the ease of introducing targeted genetic modifications facilitates the study of underlying pathways and mechanisms (Rafferty & Quinn, 2018). These specific advantages have driven the popularity of zebrafish in cardiac (patho-)physiology research and high-throughput pharmacological screening, positioning them as an essential tool for advancing our understanding of cardiovascular health and disease (Stoyek & Quinn, 2018; González-Rosa, 2022). The zebrafish is particularly useful for studies of cardiac electrophysiology, as resting heart rate, mechanisms of automaticity, sarcolemmal ion channels, action potential (AP) morphologies, calcium (Ca^{2+}) handling proteins, and intra- and extracardiac regulatory control are comparable to human (Nemtsas et al., 2010; MacDonald et al., 2017; Stoyek et al., 2022). There are of course limitations to their use for the study of some aspects of cardiac physiology, as the zebrafish heart is composed of two chambers (one atrium and one ventricle, unlike the four chambered heart of most mammals), operates at relatively low pressures (Hu et al., 2001), and cardiomyocytes (CM) in zebrafish also have poorly developed traverse tubules and ryanodine receptors that are less sensitive to Ca^{2+} than in mammals, such that even with much higher sarcoplasmic reticulum (SR) Ca^{2+} levels, the release of Ca^{2+} from the SR during excitation (via Ca^{2+}-induced Ca^{2+}-release) is limited (Brette et al., 2008; Bovo et al., 2013), and partly driven by reverse-mode (Na^{+})-Ca^{2+} exchanger current, which is much higher than in mammals (Zhang et al., 2011). Also, important to consider for studies involving genetic manipulation is the duplication of the zebrafish genome, which means that even though over 70% of zebrafish genes are orthologous to human, 24% of human genes have two orthologues in the zebrafish, leading to redundancy of gene function that can make interpretation of results difficult (Howe et al., 2013).*".

2) Line 342: 'zebrafish activation channels' probably refers to activation gates?
Amended.

3) About calcium handling: there are some more details about calcium handling in zebrafish cardiomyocytes in literature, e.g. Bovo et al. 2013. It would be good to give some more attention to that, and show how the model behaves:

- What is the fractional SR calcium release?
- Which contributes most to calcium influx, LTCC/TTCC, NCX or RyR?
- How do the absolute calcium levels of the model compare with experimental findings of the authors / in literature?

We very much appreciate this comment. Considering differences in cardiac calcium handling between zebrafish and mammals is an important aspect to include in the current study, which was largely neglected in the original paper. In our revision, we have added a paragraph to the Introduction highlighting the differences in calcium handling of zebrafish hearts compared to mammals (lines 125-130). More importantly, we have now also carefully assessed calcium handling in the model (including fractional SR calcium release and contributions to calcium influx), found in Section 3.5: Calcium Handling in the Zebrafish Model (lines 849-892) and in Figure 15 of the Results. Finally, we have added a new

section to the Discussion comparing the calcium handling characteristics of our model to what is seen in literature, including the findings of Bovo et al. 2013 (lines 969-974). We hope that these changes and additions address the reviewers' concerns. We note, however, that there is only one paper that we are aware of that reports absolute intracellular calcium concentration in the zebrafish heart (Zhang et al., 2011), in which systolic calcium was calculated based on the LTCC and not actually measured. While we make note of this finding in our paper, we are not certain of its relevance to the current study.

4) Line 383: how much bigger is GCaL in zebrafish compared to human?

G_{CaL} in the zebrafish is ~5x larger than in human. This is now noted in the manuscript (lines 532).

5) Line 480: There are indications in literature that the Na+-Ca2+ exchanger current is much larger in zebrafish, reviewed in Van Opbergen et al., PMID: 29746887

We thank the reviewer for the observation. We have now included this potential limitation of the zebrafish in lines 969-974: "*In contrast, Ca^{2+} release from the SR plays a smaller role in the CaT of zebrafish, even though SR Ca^{2+} concentration is high, due to a relatively small fractional release, as demonstrated previously in zebrafish isolated ventricular CM (Bovo et al., 2013). This finding helps explain why I_{NaCa} is larger in zebrafish than human, despite a conserved genetic sequence for NCX1 (van Opbergen et al., 2018).*".

6) Line 535: Please clarify if the number of recordings means number of hearts or impalements.

The captions of Table 2 and Table 3 have been updated to clarify this point.

Referee #2:

The authors have constructed a mathematical model for the electrophysiological behaviour of an isolated zebrafish ventricular myocyte. Unfortunately, as detailed in my specific comments below, the authors have started from an over 20-year-old human ventricular cell model (their "parent model") because of its "high computational cost-effectiveness and justified by the similarities between humans and zebrafish". However, in doing so, they should have carefully checked the availability of experimental data obtained in (adult) zebrafish ventricular myocytes and adapted their model equations based on these data. This is not the case. For example, the zebrafish sodium current is now based on experimental data from a single study in an expression system at room temperature, whereas there are experimental data from several studies on isolated adult zebrafish ventricular myocytes, even at physiological temperature.

We appreciate the reviewer's perspective on the use of the original Ten Tusscher formulation of the human ventricular action potential as our base model but note that this is an opinion not shared by all (including the first reviewer, who described it as "a leading mathematical model of the human ventricular action potential"). We absolutely agree that the model formulation should be updated based on all available, relevant experimental data in zebrafish, which we attempted to do in our original submission and we apologize if we missed some key findings. Based on the reviewer's detailed suggestions, we have made further revisions to the model using additional data, as outlined in the point-by-point response below, which we hope satisfies the reviewer's concerns.

Specific:

- Title

1) What are "whole-heart voltage-calcium data"? Are they voltage clamp and calcium transient data? And were they obtained at the whole heart level? The title should be reworded.

The title has been changed to: "*A Mathematical Model of the Zebrafish Ventricular Action Potential Based on Voltage and Calcium Measurements in the Whole Heart*".

- Key Points

1) "We have developed the first zebrafish-specific biophysical computational model of the ventricular action potential". It appears that you published an earlier version of the model in 2022 (DOI: 10.22489/CinC.2022.221), although at that time it was based on the Ten Tusscher and Panfilov (2006) human ventricular myocyte model rather than the Ten Tusscher et al. (2004) model. Furthermore, you published another version of the model in 2023 (DOI: 10.22489/CinC.2023.323), also based on the Ten Tusscher et al. (2004) model. You should at least refer to the 2022 and 2023 models and discuss any differences from the current model.

We thank the reviewer for this comment. We have made an addition to the Methods and Discussion sections to discuss the relevant differences between our previous preliminary work and the current paper (lines 333-336 and lines 958-964). In our initial work (2022), we used Ten Tusscher 2006 as the parent model, considering the availability of data and information related only to the main ionic currents involved in actional potential formation. The Ten Tusscher model was chosen because of its relative simplicity, and we used its more recent version (*i.e.*, 2006), which included updates to the calcium handling, as we assumed machinery in this is conserved in zebrafish. However, once the numerical results were compared with experimentally obtained calcium transients and their restitution curves, it became evident that this assumption was incorrect. Consequently, in our subsequent work (2023), we based our model on the 2004 version of the Ten Tusscher model, finding that the simpler calcium handling formulation allowed for an improved fit to the experimental data.

2) If "The model identified L-type calcium current (I_{CaL}) as a key current for the zebrafish action potential", what are the key currents other than I_{CaL}? Or is I_{CaL} the sole key current rather than "a key current"? Please clarify in the text.

We are sorry we were not more precise in this statement. What we meant was that our findings support the experimental suggestion that I_{CaL} has a more prominent role in the AP upstroke and in excitation-contraction coupling in zebrafish than in human. The key point has been modified to read: *"The model reinforces experimental findings, highlighting a key role of L-type calcium current in the action potential upstroke and intracellular calcium current."*.

3) What exactly is meant by "the model suggested an I_{Ks} to I_{Kr} delayed rectifier potassium current ratio of ~1:12"? Does this refer to the current amplitude during an action potential (at a given rate) or is this the conductance? However, in the latter case, it is simply what you put into the model rather than what the model "suggested". Please clarify this in the text.

We appreciate the reviewer's comment. Here we were referring to the maximal current amplitude during 2 Hz pacing. For the sake of simplicity, the key point has been modified: "*Despite conflicting evidence regarding the existence of the slow delayed rectifier potassium current in zebrafish, the model suggested its important role in repolarisation.*".

- Missing computation codes and detailed sensitivity analysis

1) I checked the "Information for Authors" of The Journal of Physiology and found out that it contains "Extra information for modelling papers". This extra information reads: "As in any experimental study, results from the model should add new scientific insights. The model should be based on physiological data where the parameters can be directly linked to experimental data. The model should be described in such a way that the results can be reproduced, i.e. all parameters are listed and any computation codes made available. Where possible there should be a detailed sensitivity analysis of the conclusions reached against the parameters proposed within the bounds of the data used to constrain them. Papers that develop integrative physiological models which link functions together or observations across scales would be encouraged." I am not sure whether the statement that "the Matlab simulation scripts are available upon request" is sufficient to meet the requirement that "any computation codes [are] made available". However, the manuscript is not accompanied by the required "computation codes". You should provide these computation codes, so that apparent errors in the listed equations (see below) can be checked against the model codes.

The 0D model will be provided in CellML format in the CellML repository and the MATLAB code for both the 0D and 1D models will be available on GitHub (https://github.com/jfrodrig1971/Zebrafish) in accordance with the journal guidelines to ensure reproducibility and clarity. At the moment, the listings have been generated but remain private; they will be made public upon acceptance of the work. The link to the repository is given in the current version of the manuscript.

- Abstract

1) Is it possible to be more specific than "published data from zebrafish"? Are these single cell and/or whole heart current and/or voltage clamp experimental data?

The data used to parameterise the model was based on published voltage-clamp data in zebrafish isolated cardiomyocytes. This is now explicitly stated in the Abstract (line 86).

2) Is it possible to be more specific than "a broad set of new experimental data, including measurements of action potential and calcium transient duration from steady-state and restitution pacing protocols"? Are these patch clamp and optical mapping data obtained at the single cell level, as I would expect when constructing a single cell model? The list of keywords below the abstract suggests that the experimental data were obtained using microelectrode recordings rather than patch clamp recordings. Is this correct?

We apologize for the confusion. Per our methods, all experiments conducted in this study were completed in whole explanted zebrafish hearts. While intracellular (microelectrode) recordings are single cell, this is done on the whole organ level. Doing these experiments in the whole heart allowed us to validate our tissue model. The text has been modified to make this more clear (lines 87-90): *"The principal ionic current conductances in the model were calibrated and validated using new experimental data, including microelectrode measurements of membrane potential and optical measurements of intracellular calcium with steady-state and restitution pacing protocols."*.

3) What are the criteria for the model to be "sufficiently computationally efficient"? I could not find these anywhere in the manuscript.

The reviewer is right, the proposed model requires a time step for explicit integration of 0.02 ms, which is among the largest for most existing action potential models and keeps the number of state variables to a minimum. However, it is not a sufficient criterion to judge the model as computationally efficient. We have removed the sentence from the manuscript since it is not relevant to the work.

- Introduction

1) Line 92: It is not very common to write "wet-dry investigations" when referring to a combination of in vitro and in silico experiments, which you probably do. Consider rewording.

While the term 'wet-dry' has been used by others in the literature, in the hopes of being succinct and clear, we have changed this to "*combined experimental-computational*".

2) Lines 99-102: "For this, the Ten Tusscher and Panfilov formulation of the human ventricular AP (TP04) (Ten Tusscher et al., 2004) - chosen for its high computational cost-effectiveness and justified by the similarities between humans and zebrafish - was selected as a parent model." This 'parent model' was developed over 20 years ago, based on the experimental data that were available at the time. Since then, several human ventricular cell models have been developed that are based on more recent experimental data. It may well be that these models contain descriptions of individual ionic currents that are almost as computationally inexpensive, but are much more accurate. Have you checked these newer models for such improved ionic current formulations? What was the outcome? These more recent models do include not only the model by O'Hara et al. (2011), which is listed in your references, but also the models by Tomek et al. (2019; DOI: 10.7554/eLife.48890) and Bartolucci et al. (2020, DOI: 10.3389/fphys.2020.00314), which are not.

While more recent human action potential models do exist, we have carefully chosen the TP04 model as the parent model for the development of our zebrafish-specific model. While indeed the TP04 model is now 20 years old, many of the ion current formulations in the newer models (such as O'Hara 2011) are based on or still use the identical formulation as the TP04 model. We subscribed to the commonly held adage that one should only make a model as complicated as necessary and believe that this applies in this case. By using a more complex parent model of the human cardiomyocyte as our base model, we would be adding unnecessary complexity. In our revised manuscript, we now include a section outlining the major differences between human and zebrafish, namely in the intracellular calcium handling mechanisms. Newer and more complex action potential models, such as the O'Hara model, offer sub-cellular compartmentalisation of calcium, that we do not know exists in the zebrafish. Thus, we believe that the TP04 model is an appropriate parent model to use for our novel computational model of the zebrafish action potential and have now added additional justification to the manuscript.

- Methods

1) Line 159: "Intracellular microelectrode recordings of V were performed as previously described (Stoyek et al., 2024)." This is the first appearance of "V". What does it denote?

We thank the reviewer for picking up on this error. The sentence now reads: *"Intracellular microelectrode recordings of V_m were performed as previously described (Stoyek et al., 2024)."* V_m is cited for the first time in line 153.

2) Line 178: "RMP (stable Vm during diastole)" This is the first appearance of "Vm". Apparently, it denotes membrane potential. Is this correct? What is the difference between V and Vm?

We thank the reviewer for picking up on this error. Both V and V_m stand for transmembrane potential. We corrected the text by using only V_m, which is cited for the first time in line 153.

3) Line 187: "the excitation-contraction uncoupler {plus minus}blebbistatin". Apparently, you mean (+/−)-blebbistatin. Write it as such.

Amended.

4) Line 190: "10 µM Rhod-2 AM (ab1427870, abcam, Cambridge, UK; Kd≈2.30 µM)". This is the first appearance of "Kd". What does it denote? The name of the company is Abcam (see their website), although their logo shows "abcam".

Amended.

5) Line 191: "5 µM Fluo-5F AM (20560, AAT Bioquest, Pleasanton, USA; ; Kd≈0.57 µM)". Is something missing between the two semicolons?

Thank you for the very careful reading of our manuscript! That was a typo, which is now corrected.

6) Figure 1: "Tropin" and "Citosol" should probably read "Troponin" and "Cytosol".

Calsequestrin, calmodulin, and troponin have been deleted from the image since they do not have a direct role in the model and may generate confusion.

7) Legend to Figure 1: "Schematic representation of the adult zebrafish ventricular cardiomyocyte." This legend needs an explanation of all the abbreviations shown and the role of "Calsequestrin", "Calmodulin" and "Tropin" play in the model.

Amended.

8) Lines 275-276: "The model was implemented in Matlab. For the 0D simulations, a stiff solver with an adaptive time step was used." Which version of MATLAB? Which "stiff solver"?

We used MATLAB R2022a for our simulations. We have modify the text to specify the version. Specifically, we utilized the ODE15s solver, which is a good option for most stiff systems and includes an adaptive time-stepping feature. This information, along with the MATLAB code for the model, will be provided in both CellML and GitHub, as per the journal guidelines, to ensure reproducibility and clarity. This is now addressed in lines 374-375 and 382-384.

9) Lines 260-261: "m, h, and j for INa, dT and fT for ICaT, dL, fL, and fCa for ICaL, xs for IKs, and xr1 and xr2 for IKr". This is the first appearance of these abbreviations. They should either be explained here or the reader should be referred to the section where the explanation can readily be found.

We thank the reviewer for picking up on this omission. We have added a reference to Section 2.8, where all of the ionic currents and respective gates are explained in detail.

10) Lines 266-268: "The effective tissue conductivity was assumed to be σ = 3.012 · 10−4 mS, which results in a conduction velocity of 1.27 cm/s, which is consistent with our measurements (1.25 cm/s) and data reported in the literature (Panáková et al., 2010)." Was this value of σ chosen because it yields a conduction velocity near 1.25 cm/s? What are the "data reported in the literature"? Are these "data reported in the literature" data on conductivity or on conduction velocity?

While we indeed set the tissue conductivity to generate a conduction velocity that matched values measured by us and reported in the literature, the paragraph in question has been updated - along with the model - as we have identified an error in the calculation of the experimentally measured conduction velocity. The paragraph now reads: *"The effective diffusion coefficient was set to* $D = 1.76 \cdot 10^{-2}$ *mm²/ms, resulting in a conduction velocity of 126.8 mm/s, which is consistent with our experimental measurements (125 mm/s) and conduction velocity reported in the literature (Kikuchi et al., 2010; Wang et al., 2011)"*. In addition, the reference to Panáková et al., 2010 has been removed, as this paper refers to conduction velocity measured in zebrafish embryo hearts.

11) Lines 284-286: "To calibrate model parameters, an initial Montecarlo simulation was performed to identify the most suitable model, which was then followed by a manual tuning of the parameters." This is a rather vague description. Given the aforementioned requirement that "the model should be described in such a way that the results can be reproduced", a more detailed description should be provided. Also, write "Monte Carlo" (as in the next section) instead of "Montecarlo".

We apologize for the lack of clarity. Once the steady-state variables and time constants for the ion channel gates were defined by fitting to the voltage-clamp data obtained from the literature, a Monte Carlo simulation was performed to identify the combination of ion channel conductances and Ca^{2+} dynamics and cytoplasmic volume parameters that most closely matched our experimental results. Then, the maximum conductance of the main ionic currents was manually adjusted to better fit the model's different restitution characteristics.

For clarity, we have modified the text in lines 398-409 to read: "*Next, to calibrate the ion channel conductances and Ca2+ handling parameters, a Monte Carlo simulation was conducted, involving variation of the maximum conductance of all currents and Ca2+ dynamics parameters to identify combinations that fit within the range of experimental data, while ensuring model stability. The protocol involved stimulating the model with 110 pulses at a frequency of 1 Hz, followed by a 3-second period without stimulation. The last 10 action potentials and the final 3 seconds of the simulation were saved for analysis. A given combination of parameters was considered valid if the results demonstrated the absence of alternants in the last ten action potentials and a lack of pacemaking behaviour following the cessation of stimulation. This process was followed by manual parameter tuning, where the maximum conductance of the principal ionic currents was adjusted to better fit the model's different restitution characteristics.*".

12) Lines 291-293: "Monte Carlo method was used with 5000 combinations of 17 parameters representing the maximum conductance of major currents and determinants of Ca2+ dynamics (i.e., GNa, GCaL, GCaT, GKr, GKs, GK1, GpCa, GbNa, GbCa, GNaK, GNaCa, among others)." What are these six "others" among the 17 model parameters? Why was this set of parameters chosen?

We identified and corrected a mistake regarding the parameters used. The analysis included 18 parameters selected for their direct influence on cardiomyocyte electrophysiological behaviour. In addition to the 11 ionic conductances, 7 parameters related to Ca^{2+} dynamics and cytoplasmic volume were considered. To improve clarity, the sentence has been rewritten to explicitly list all parameters: "*A the Monte Carlo method was used, involving 5000 combinations of 18 parameters representing the maximum conductance of major currents (i.e.,* G_{Na}, G_{CaL}, G_{CaT}, G_{Kr}, G_{Ks}, G_{K1}, G_{pCa}, G_{bNa}, G_{bCa}, G_{NaK}, G_{NaCa}*), the cytoplasmic volume,* V_c*, and key determinants of* Ca^{2+} *dynamics (i.e.,* V_{leak}, V_{mxu}, K_{up}, τ_g, buf_c*, and* $kbuf_c$*), which were varied within a range of ± 30% (except for* V_c*, which was varied within a range of ± 50%).*"

13) Lines 301-302: "for the block of IKr with chromanol 293B, IKs with E4031". Chromanol 293B blocks IKs, whereas E4031 blocks IKr. Please correct.

Amended.

14) Lines 319-320: "For most of the currents, the same formulation proposed in the original TP04 human ventricular AP model was utilised." This is most unfortunate because, as stated above, the "TP04 human ventricular AP model" was developed over 20 years ago, based on the experimental data that were available at that time.

We respectfully disagree with this perspective, as we believe that the TP04 model is an appropriate parent model as it is as complicated as necessary for the given purpose. Please see the response to Comment 2 regarding the Introduction (above), which deals with a similar comment from the reviewer.

15) Lines 326-328: "The voltage-gated fast Na^+ channels play a crucial role in the rapid upstroke of the ventricular cardiomyocyte AP (Phase 0), and they are partly responsible for determining the velocity of impulse transmission between cells (Vornanen & Hassinen, 2016)." This is one of those (there are more) annoying references in the manuscript. Annoying, in this particular case, because Vornanen & Hassinen (2016) simply make a similar statement, referring to two papers that, when checked, appear to be irrelevant to AP conduction velocity. Authors should provide references to relevant papers that they themselves have checked, and avoid reference after reference that ends up in irrelevant papers.

We apologise for the annoyance, and have now ensured that we are referencing the most relevant literature throughout the manuscript.

16) Lines 341-343: "using the steady-state of zebrafish activation channels expressed in Chinese Hamster Ovary (CHO) cells as reported in Chopra et al., 2007." I have several comments. First, it would be helpful to write "as reported in Chopra et al., 2007 (their Figure 6 and Table 6)", so that your readers know which data you are referring to. Second, and more importantly, you should inform your readers how you have dealt with differences between the experimental conditions and your model. For example, the voltage clamp experiments of Chopra et al. (2007) were performed at room temperature, whereas your model simulates a myocyte at 301 K (28{degree sign}C). How did you correct for the associated differences in sodium current kinetics? Third, and perhaps most importantly, I do not understand why you based your model on 2007 data obtained in an expression system (expressing only NaV1.5, and therefore not NaV1.4)

when there are more recent data obtained in studies of isolated adult zebrafish ventricular myocytes. One such study is that of Haverinen et al. (2018). In their paper (DOI: 10.1016/j.pbiomolbio.2018.04.002), they even present experimental data on the fast time constant of inactivation, which the authors now base on the recovery from inactivation obtained with their 'parent model'. Other more recent studies with data on the sodium current in isolated adult zebrafish ventricular myocytes are those by Haverinen et al. (2021) and Chiang et al. (2024) with DOI codes of 10.1242/jeb.241067 and 10.1161/CIRCRESAHA.123.323231, respectively.

We thank the reviewer for these comments. First, we have added a reference to Figure 6 and Table 6 in the work of Chopra et al. (2007) on line 474-475 for clarity.

The reviewer is right; first of all, data from Chopra et al. (2007) were collected at a different temperature; for this reason, the previous data has now been scaled to match our simulation temperature. Particularly, lines 483-488 now report: "*The experimental data from Chopra et al., 2007a, were fit with two exponentials, generating two distinct time constants. Additionally, scaling was applied as the experimental data were collected at 21-23 °C (Chopra et al., 2007a) rather than 28 °C. The curves were scaled with a factor of 0.758, corresponding to a Q_{10} = 1.74, as reported by Haverinen et al., 2018c*".

Moreover, the other papers identified by the reviewer have now been included in the experimental data used to derive the formulations for the channel inactivation time constants. The formulations for both state variables and time constants have been modified, as have all the results obtained using these adjustments (see the new Figure 2 in the revised manuscript). To be precise, in the case of time constants, Haverinen's paper presented the same type of data as Chopra's, specifically a recovery-from-inactivation curve that was used to derive the tau values. However, as with Chopra's data, these values are associated with a limited voltage range, making it impossible to fit the data alone. Therefore, the data were used, but as before, the TP04 formulation was utilized with appropriate modifications to fit the experimental data. Specifically, for τ_h the curve that best fits all the experimental data is the same TP04 formulation, but multiplied by a factor of 3.5, while for τ_f the TP04 formulation was shifted to the right by 9 mV and scaled with a factor of 0.8. All results have been updated to incorporate these adjustments.

17) Lines 351-352: "the formulations for τ_h and τ_j were obtained by translating and scaling the TP04 equations". This process requires a better explanation than "by translating and scaling". How were the original equations translated and scaled? Did this process include a correction for the differences in temperature? If so, what was the Q10; and why that value?

We apologize for the misunderstanding. The process was exactly as described. Since there were no data available for τ_h and τ_j, but only the recovery from inactivation curve evaluated at V = -30 mV (From Chopra et al., 2007, Fig 6), we derived the two time constants from this curve and thus obtained the two experimental values for τ_h and τ_j, as shown in Figures 2d and 2e. With only this data available, we used the original TP04 formulation, shifting it along the x-axis and scaling it to match this value. Given the lack of additional information, we opted to retain the original formulation as much as possible, which, considering the excellent results obtained, seems to suggest that the formulations for humans in the TP04 model and zebrafish are indeed very similar.

Further, as reported in the previous comment, since data from Chopra et al. (2007) were collected at a different temperature, the data has now been scaled to match our simulation temperature. Particularly, lines 483-488 now report: "*The experimental data from Chopra et al., 2007a, were fit with two exponentials, generating two distinct time constants. Additionally, scaling was applied as the experimental data were collected at 21-23 °C (Chopra et al., 2007a) rather than 28 °C. The curves were scaled with a factor of 0.758, corresponding to a Q_{10} = 1.74, as reported by Haverinen et al., 2018c*".

Moreover, as suggested in the previous comment, we integrated the works of Haverinen et al. (2018 and 2021) to derive the formulations for the inactivation time constants. However, as said in the previous comment, these values are associated with a limited voltage range, making it impossible to fit the data alone. Therefore, even with these new data, the TP04 formulation was utilized as a starting point. For clarity, lines 488-493 have been modified to explain in detail what has been done.

18) Figure 2: Again, I have several comments. First, when including experimental data, you should present all values as mean {plus minus} standard error of the mean (SEM), rather than just the mean values. Second, the labels should be more specific. This applies not only to Figure 2, but also to other figures containing experimental data. The ordinate label of panel A should read something like "steady-state (in)activation" and it should be indicated which is the activation curve and which is the inactivation curve. Panels B, D and E should not all three be labelled "t [ms]", but something like "Activation time constant [ms]", "Fast inactivation time constant [ms]" and "Slow inactivation time constant [ms]". Panel C lacks an ordinate label. Its ordinate label should read something like "Normalized current". Third, the slope of the inactivation curves changes suddenly near −30 mV, which seems rather unphysiological. I

understand that this is probably due to the 'parent model', but this should be further encouragement to reformulate the associated equations.

We appreciate the reviewer's comment. We have modified the labels of the figures as suggested. In addition, the figures now contain the suggested relevant information, to be in line with other papers presenting the development of a computational model. When possible, we have added to the figures the uncertainty values and noted what they represent in the figure caption. Regarding τ_h, the change in slope of the curve around –30 mV was, exactly as mentioned, due to the formulation of τ_h in the parent model. Without any additional experimental data to inform curve fitting, we find it best to keep with the parent model. With new experimental data now incorporated, the change in slope is the same as TP04 (- 40 mV).

19) Lines 361-363: "Since in the adult human heart, ICaT is mainly limited to the sinoatrial node and conductive pathways (Vornanen & Hassinen, 2016)". What about the AV node?

We thank the reviewer for this question. Since the AV node is considered by many to be part of the conductive pathways, we thought it was implicit in this statement. However, to avoid potential misunderstandings given the specific mention of the sinoatrial node, we now also explicitly mention the atrioventricular node.

20) Lines 369-371: "In order to fit the experimental behaviour of ICaT in zebrafish with the rabbit model formulation, data from Haverinen et al., 2018 were taken, and the experimental patch-clamp protocol was modelled to fit the current-voltage curve as shown in Figure 3". What exactly did you do "to fit the current-voltage curve"? Why did you not consider the electrophysiological data from Nemtsas et al. (2010; DOI: 10.1016/j.yjmcc.2009.08.034)?

We thank the reviewer for this question. To obtain the fitting of the I_{CaT} current versus voltage, we took the formulation from Demir et al. (1994) for the rabbit sinoatrial node and used it to numerically simulate a patch-clamp experiment by incrementing the voltage in steps. This explains why Figure 3 initially appeared jagged rather than smooth. (This has now been modified; please see the comment below). At this point, we manually adjusted the formulation of Demir et al. (1994) to match the experimental data.

Accordingly, lines 513-519 now read: *"To fit the experimental behaviour of I_{CaT} in zebrafish to the rabbit model formulation, data from Haverinen et al., 2021(found in Figure 3 of the paper) and Nemtsas et al., 2010b (found Figure 4 of the paper) were used. The experimental patch-clamp protocol was modelled by incrementing voltage in steps to fit the current-voltage curve (Figure 3). The formulation was then numerically simulating a patch-clamp experiment. The formulation was then manually adjusted to ensure that the simulated patch-clamp protocol produced an I-V relationship consistent with the experimental data."*.

Moreover, the reviewer is correct, and thus, as now indicated in the text, the data from Nemtsas et al. (2010b) has been integrated with the data from Haverinen et al. (2021) confirming the previously observed trend.

21) Figure 3: Why is the "computational current-voltage (I-V) curve" not shown as a smooth curve? It seems to consist of straight lines that connect simulation results obtained at −80 mV, −70 mV, −60 mV, etc. And is this really the best fit that you could obtain? At −50 mV, for example, the experimental current density is >3 times larger than the "numerical" current density.

This was a plotting error that was overlooked, which has now been fixed. As explained in the previous comment, no direct fitting of the I-V curve was performed. Instead, a patch-clamp protocol was numerically simulated, where the formulations for steady-state and time constants from Demir et al. (1994) were used as a starting point and modified to obtain an I-V curve consistent with the experimental data.

22) Lines 395-396: "experimental data from Kompella et al., 2021 and Zhang et al., 2011". There are more data available on the L-type calcium current than those obtained by Kompella et al. (2021) and Zhang et al. (2011). Just one example, resulting from a straightforward Google Scholar search, is the study by Koopman et al. (2021; DOI: 10.1073/pnas.2018220118), which provides experimental data on the current density. In that same study, experimental data on the current density of the rapid delayed rectifier K+ current inward rectifier K+ current are n. Why were such other data not considered?

We thank the reviewer for the comment. In the work of Koopman et al. (2021), particularly in Figure 6H, they evaluated the total potassium current (I_K) instead of the single contribution. This is also stated in the Methods section of Koopman et al (2021): "*[...] voltage clamp measurements were performed without specific channel blockers or modified solutions.*" For this reason, data from that paper were not considered in the formulation of our model. Regarding the L-type calcium data reported by Koopman et al. (2021), as explained in response to the next comment, in this case, the I-V relationship would represent a "result" rather than a tool for deriving the steady-state and time constants formulations.

23) Figures 4-6: Why are experimental steady-state current-voltage relationships not shown? Such experimental data are available.

The rationale behind Figures 2 to 7 is to display the experimental data that were used to fit the numerical curves. Specifically, in the case of I_{CaT}, since only data on the I-V relationship are available, these were used to modify the formulations of the $d_{T\infty}$ and $f_{T\infty}$ gates. For I_{K1}, as it does not involve gates, the I-V relationship was used to derive the equation for $x_{K1\infty}$. In all other cases, the I-V relationship would represent a "result" rather than a tool for deriving the steady-state and time constants formulations.

- Results

1) Figure 8, Table 2: Your simulation data agree well with your microelectrode recordings. However, there are many more zebrafish action potential recordings available, including patch clamp recordings from isolated myocytes at physiological temperature. How do your simulation data agree with these patch clamp data?

You are correct, there are other ventricular action potential data from the adult zebrafish available in the literature. For instance, there is data available from our group (Stoyek et al., 2022; doi: 10.3389/fphys.2022.818122, and Kopton et al., 2018; doi.org/10.3389/fphys.2018.01806), as well as others, such as Vornanen and Hassinen, 2016 (doi.org/10.1080/19336950.2015.1121335), Nemtsas et al., 2010 (doi.org/10.1016/j.yjmcc.2009.08.034), and Kompella et al., 2021 (doi.org/10.1085/jgp.202012733) that agree with our experimental measurements, and thus our newly developed model.

We now mention this experimental data in lines 924-928, presenting ranges showing that our data and model fit well within the previously reported experimental range.

2) Figure 9, Table 3: Your simulated calcium transients agree well with your calcium transient recordings. However, there are many more calcium transient recordings available in the scientific literature. How do your simulation data agree with these additional data?

Again, you are correct, there are other ventricular calcium transient data from the adult zebrafish available in the literature (although less than for the action potential). This includes data from Rayani et al., 2018 (doi.org/10.1016/j.pbiomolbio.2018.07.006), Lin et al., 2015 (doi.org/10.1152/ajpregu.00001.2015), and Stoyek et al., 2024 (doi.org/10.3390/genes15030280). In these cases, however, they used the relatively high affinity calcium-sensitive dye Rhod-2, which produced calcium transients that are similar to ours when we made recordings with the same dye. To our knowledge, however, no one has used a lower affinity calcium-sensitive dye such as Fluo-5F in the heart of adult zebrafish, which we used in the present study, and which produced shorter calcium transients due to a lower degree of calcium buffering.

We now mention this experimental data in lines 952-955, presenting ranges showing that our data and model fit well within the previously reported experimental range.

3) Lines 590-592: "Unfortunately, there are no available experimental recordings for these currents to allow for a direct comparison with the model". I tend to disagree. The paper by Kompella et al. (2021), which is in your reference list, shows action potential clamp recordings of the rapid delayed rectifier potassium current. There may also be other studies that show such recordings.

We apologize for the misunderstanding. When we stated that "there are no comparable current-related data," we were referring specifically to the absence of such data during the action potential, not during a voltage clamp. We did, in fact, cite the paper by Kompella et al. (2021) for I_{CaL}, where experimental data on steady-state activation and inactivation curves were reported and used in our manuscript. Kompella et al. also reported data on I_{K1}; specifically, in Figure 4, they presented an "AP-like voltage-clamp," which, however, is not suitable for comparison with the numerical data we obtained during the actual action potential.

- Discussion

Lines 679-681: "Here we describe the development of the first computational model of the zebrafish ventricular AP, considering the most recent patch-clamp data available in the literature for parameterisation of the major ionic currents." This is not true, as explained in my comments above. The authors should have done a solid search for such "recent patch-clamp data" and would have found many more studies than the ones they refer to.

The revised version of the manuscript now integrates the additional relevant patch-clamp data that was previously overlooked, as described above.

- Appendix 1

I have not checked all of the equations listed in Appendix 1 against the 'parent model' equations, which will probably be publicly available somewhere. However, I did notice an obvious error in one of the first lines of the appendix. This is in the Nernst potential for ECa, where the charge of the calcium ions is not correctly included. Given this obvious mistake, I doubt that the remaining equations listed in Appendix 1 are all correct. This is another reason why the computation codes should be made publicly available.

We apologize for the errors in this Appendix. In particular, V_m is now being used to indicate membrane potential and ionic charges have been added to the appropriate equations.

- Appendix 2

Similarly, I have not checked all of the parameters listed in Appendix 2 against the parameters of the 'parent model'. However, just looking at the first few lines of that appendix, I noticed that the value of the parameter σ is listed here as the computer code-like "3.012e-04 mS" (without superscript), whereas it appears as "3.012·10−4 mS" (to be preferred) in the body text. Given the unit of mS, it should be conductance, but given the Greek σ to denote it, it should be conductivity. This is confusing and should be checked carefully.

We apologize for the confusion. In the current version, the governing equations for the 0D and 1D models are written in a slightly different manner, in order to have ionic currents listed as mA/mF. In this formulation the parameter s is substituted by the parameter *D*, a "diffusion coefficient", which is proportional to tissue conductivity. The value of D has been set to obtain a conduction velocity similar to that measured in the experiments.

- References

The list of references is sloppy in numerous respects:

We thank the reviewer for their careful reading of our manuscript; we have revised the references.

(a) The references are not completely in journal style. In particular, attention to punctuation is required.

Amended.

(b) The alphabetical order is inconsistent. De la Cruz et al. (2023), for example, appears between Kompella et al. (2021) and Nemtsas et al. (2010). Similarly, Tsai et al. (2011) should appear after Ten Tusscher et al. (2004), not before. Van Opbergen et al. (2018) should be alphabetically ordered based on "Van" rather "Opbergen".

Amended.

(c) No attention has been paid to subscripts, superscripts and italics in the titles of papers.

Amended.

(d) The format of the paper titles is not consistent. Some appear in Sentence case, others in Title Case.

Amended.

(e) The naming of journals is not consistent. Pflügers Archiv appears as "Pflugers Arch", but also as "Pflugers Arch Eur J Physiol".

Amended.

(f) The format of author names is inconsistent. In the first references, as an example, the author "Abramochkin DV" appears as "Abramochkin D V.".

Amended.

(g) Babcock et al. (1997): plus sign missing in the title.

Amended.

(h) Carro et al. (2016): no journal name, volume number, page numbers.

Amended.

(i) Chopra et al. (2007): no volume and page numbers.

Amended.

(j) De la Cruz et al. (2023): no volume and page numbers; DOI is 10.3390/IJMS241512092, not "10.3390/IJMS241512092/S1".

Amended.

(k) Demir et al. (1994): no volume and page numbers.

Amended.

(l) Gut et al. (2017): incomplete page numbers.

Amended.

(m) Haverinen et al. (2018): no volume and page numbers.

Amended.

(n) Howe et al. (2013): incomplete page numbers.

Amended.

(o) Howe et al. (2013): the journal instructions state "When the work has 21 or more authors, include only the first 19 names, an ellipsis, and the final name (with no ampersand)."

Amended.

(p) O'Hara et al. (2011): the first author's name is O'Hara, not "O'hara".

Amended.

(q) Panáková et al. (2010): the journal name is Nature, not "Nat 2010 4667308".

This citation has been delated.

(r) Pozzan & Rudolf (2008): no journal name, volume number, page numbers.

Amended.

(s) Romero et al. (2009): journal name is Am J Physiol Heart Circ Physiol, not "Am J Physiol Hear Circ Physiol".

Amended.

(t) Stamm et al. (2003): title starts with Cytosolic, not "Cytosolic".

Amended.

(u) Stoyek et al. (2024): no volume and page numbers.

Amended.

(v) Sun et al. (1997): no journal name, volume number, page numbers.

Amended.

(w) Ten Tusscher et al. (2004): no volume and page numbers.

Amended.

(x) Tsai et al. (2011): no journal name, volume number, page numbers.

Amended.

(y) Zhang et al. (2000): no volume and page numbers.

Amended.

(z) Zhang et al. (2011): no volume and page numbers.

Amended.

- Acknowledgments

"The authors thank Prof. Arie Verkerk for sharing patch-clamp inactivation data of L-type calcium channels." Where exactly in the manuscript were these "patch-clamp inactivation data" used? Under which conditions were these data obtained?

We thank the reviewer for this comment. This novel data was used to fit the (in)-activation curves and the inactivation time constant of the LTCC in Figure 4. We have now included Dr. Verkerk as one of the authors of the paper, and provided details in the text on how this data was obtained (lines 166-168, lines 180-191, lines 215-228, and lines 242-253). Consequently, the acknowledgments section has been removed.

- Supporting Material, Figure S1

The legend to Figure S1, or the figure itself, should make clear what the two coloured lines are. Are they "Model 0D" and "Model 1D", as in Figure 11?

We apologize for the confusion. Yes, the colours are the same as in Figure 11. The supplementary figure has been now incorporated in Figure 11.

- Supporting Material, Figure S2

The legend to Figure S2 should clarify the conditions under which the calcium transients were obtained. The main manuscript only tells us that "Figure S2 in the Supporting Material shows computational results with increased Ca2+ buffering to mimic the effect of Rhod-2", without quantifying the stimulus frequency at which these transients were obtained. Apparently, it is 1 Hz, since section 2.4 of the main manuscript states that "an abbreviated pacing protocol was used to compare Ca2+ transients measurements to Rhod-2: a train of 100 stimuli was delivered to the heart, with cycle lengths of 1000, 500, and 250 ms", in relation to the duration of the transient observed in the figure.

The supplementary figure has been now incorporated into Figure 9. Regarding all of the plotted figures, they are presented for a BCL of 500 ms (2 Hz). Lines 708-709 now clarify what numerical model parameters were adjusted to mimic the calcium buffering with the higher affinity dye.

- Supporting Material, Figure S3

The legend to Figure S3 should clarify the conditions under which [Na+]i and [K+]i were obtained. The main manuscript says: "In particular, the ability of the model to stabilize the intracellular Na+ and intracellular K+ at different stimulation frequencies was verified (see Figure S3 in the Supporting Material)." So, where are these "different stimulation frequencies" in Figure S3? Or is Figure S3 just for a single stimulation frequency? And, if so, what is this specific stimulation frequency? Why do [Na+]i and [K+]i start at around 11.5 and 109 mM, respectively? Are these the concentration values obtained under steady-state resting conditions?

This is now clarified in the text (lines 1016-1018). Overall, the intracellular Na^+ and K^+ stabilised in the model at varying pacing frequencies, but the figure presents only the stabilisation during steady-state pacing at 2 Hz. The starting concentrations for intracellular Na^+ and K^+, being 7.99 and 112.47 mM, are the intracellular ion concentrations.

Dear Dr Cestariolo,

Re: JP-RP-2025-287624R1 "A Mathematical Model of the Zebrafish Ventricular Action Potential Based on Voltage and Calcium Measurements in the Whole Heart" by Ludovica Cestariolo, Zachary David Long, Arie O Verkerk, Jose Maria Ferrero, T Alexander Quinn, and Jose Felix Rodriguez Matas

Thank you for submitting your manuscript to The Journal of Physiology. It has been assessed by a Reviewing Editor and by 2 expert referees and we are pleased to tell you that it is potentially acceptable for publication following satisfactory major revision.

Please advise your co-authors of this decision as soon as possible.

The referee reports are copied at the end of this email.

Please address all the points raised and incorporate all requested revisions or explain in your Response to Referees why a change has not been made. We hope you will find the comments helpful and that you will be able to return your revised manuscript within 2 months. If you require longer than this, please contact journal staff: jp@physoc.org. Please note that this letter does not constitute a guarantee for acceptance of your revised manuscript.

Your revised manuscript should be submitted online using the link in your Author Tasks: Link Not Available. This link is accessible via your account as Corresponding Author; it is not available to your co-authors. If this presents a problem, please contact journal staff (jp@physoc.org). Image files from the previous version are retained on the system. Please ensure you replace or remove any files that are being revised.

If you do not wish to submit a revised version of your manuscript, you must inform our journal staff (jp@physoc.org) or reply to this email to request withdrawal. Please note that a manuscript must be formally withdrawn from the peer review process at one journal before it may be submitted to another journal.

TRANSPARENT PEER REVIEW POLICY: To improve the transparency of its peer review process, The Journal of Physiology publishes online as supporting information the peer review history of all articles accepted for publication. Readers will have access to decision letters, including Editors' comments and referee reports, for each version of the manuscript, as well as any author responses to peer review comments. Referees can decide whether or not they wish to be named on the peer review history document.

ABSTRACT FIGURES: Authors are expected to use The Journal's premium BioRender account to create/redraw their Abstract Figures. Information on how to access this account is here: https://physoc.onlinelibrary.wiley.com/journal/14697793/biorender-access.

This will enable Authors to create and download high-resolution figures. If authors have used the free BioRender service, they can use the instructions provided in the link above to download a high-resolution version suitable for publication.

The link provided should only be used for the purposes of this submission. Authors will be charged for figures created on this account if they are not related to this manuscript submission.

LANGUAGE EDITING AND SUPPORT FOR PUBLICATION: If you would like help with English language editing, or other article preparation support, Wiley Editing Services offers expert help, including English Language Editing, as well as translation, manuscript formatting, and figure formatting at www.wileyauthors.com/eeo/preparation. You can also find resources for Preparing Your Article for general guidance about writing and preparing your manuscript at www.wileyauthors.com/eeo/prepresources.

RIGOUR AND REPRODUCIBILITY: We recommend authors consult The Journal's Rigour and Reproducibility requirements to ensure that their manuscript is suitable for publication: https://physoc.onlinelibrary.wiley.com/pb-assets/hub-assets/physoc/documents/TJP-Rigour-and-Reproducibility-Requirements.pdf

REVISION CHECKLIST:

Check that your Methods section conforms to journal policy: https://jp.msubmit.net/cgi-bin/main.plex?form_type=display_requirements#methods.

Check that data presented conforms to the statistics policy: https://jp.msubmit.net/cgi-bin/main.plex?form_type=display_requirements#statistics.

Upload a full Response to Referees file. To create your 'Response to Referees': copy all the reports, including any comments from the Senior and Reviewing Editors, into a Microsoft Word, or similar, file and respond to each point, using font or background colour to distinguish comments and responses and upload as the required file type.

Please upload two versions of your manuscript text: one with all relevant changes highlighted and one clean version with no changes tracked. The manuscript file should include all tables and figure legends, but each figure/graph should be uploaded as separate, high-resolution files.

You may also upload:

- 'Potential Cover Art' for consideration as the issue's cover image
- Appropriate Supporting Information (Video, audio or data set: see https://jp.msubmit.net/cgi-bin/main.plex?form_type=display_requirements#supp).

We look forward to receiving your revised submission.

If you have any queries, please reply to this email and we will be pleased to advise.

Yours sincerely,

Bjorn Knollmann
Senior Editor
The Journal of Physiology

REQUIRED ITEMS

(1) You must start the Methods section with a paragraph headed Ethical approval (https://jp.msubmit.net/cgi-bin/main.plex?form_type=display_requirements#methods).

Research must comply with The Journal's policies regarding animal experiments (https://physoc.onlinelibrary.wiley.com/hub/animal-experiments) and adherence to these policies must be stated in the manuscript.

Authors should confirm in their Methods section that their experiments were carried out according to the guidelines laid down by their institution's animal welfare committee, including an ethics approval reference number. The Methods section must contain a statement about access to food, water and housing, details of the anaesthetic regime: anaesthetic used, dose and route of administration, and method of killing the experimental animals.

(2) Please provide a legend to accompany your Abstract figure; this can be included in the main article (Word) file, along with the other figure legends.

EDITOR COMMENTS

Reviewing Editor:

The revised version of your manuscript was reviewed by the two expert referees again and again significant number of issues remain that need to be addressed for further consideration of this manuscript, including:

- discrepancies between responses and data presented in the manuscript and between different parts of the manuscript. It is surprising that none of the 6 authors have identified these during the revision process;

- suboptimal reflection of the content of the manuscript in the title and key points;

- code not being available to the reviewers.

Please also see 'Required Items' above.

Senior Editor:

While somewhat improved, the revised manuscript still has major deficiencies, some of which had been raised in the first

review.

REFEREE COMMENTS

Referee #1:

I think this manuscript will be of great benefit to this research field, and my comments raised in the first round have all been addressed. The additional data included in the revised manuscript only further enhance the work.

Referee #2:

EVALUATION

- GENERAL

A manuscript that describes a newly developed mathematical model should be unambiguous in all respects. A trained biomathematician should be able to program the model from the description of the model provided in the manuscript. This is still not the case, as detailed in my specific comments below.

In their response to my original comments, the authors stated that they "were referring to the maximal current amplitude during 2 Hz pacing" when they brought up a "IKs to IKr delayed rectifier potassium current ratio of ~1:12". Such statement regarding this ratio also appears in the revised manuscript ("In our computational model, the IKs:IKr density ratio is 1:12"). However, this is at odds with Figure 10, in which the current amplitude of both IKs and IKr is 1.0 µA/µF during 2 Hz pacing. An obvious discrepancy like this, which should have been noted by at least one of the six authors (all involved in review and editing, according the "Authorship Contribution Statement" listed at the end of the manuscript), reduces my confidence in the quality of the manuscript.

An important shortcoming of the model is that it does not include the late sodium current of zebrafish ventricular cardiomyocytes. This is an important current that has been linked to various ventricular arrhythmias in the human heart and is therefore important to be included in a model of zebrafish ventricular myocytes if regarded "a useful tool for future studies of cardiac electrophysiology and its dysfunction" (Discussion, last line).

- SPECIFIC

- Title

The authors have changed the title from "A mathematical model of the zebrafish ventricular action potential from whole-heart voltage-calcium data" to "A mathematical model of the zebrafish ventricular action potential based on voltage and calcium measurements in the whole heart". The new title suggests that the model is purely based on voltage and calcium measurements in the whole heart (without measurements of the underlying ion currents). However, when going through the manuscript one learns that the model is also based on patch-clamp data obtained from isolated zebrafish ventricular cardiomyocytes and "from zebrafish activation gates expressed in Chinese Hamster Ovary (CHO) cells". Actually, the patch-clamp data shown in Figure 4 seem to have been obtained as part of the present study. The title should be reworded accordingly.

- Key Points

"We developed the first zebrafish-specific computational ventricular action potential model using newly generated whole-heart voltage-calcium experimental data." Which are these "whole-heart voltage-calcium experimental data"? Are these the "voltage and calcium measurements in the whole heart" of the updated title of the manuscript? If so, what about your newly generated experimental data on isolated cells?

- Abstract

1) At the bottom lines of the Abstract you have written: "Overall, the novel zebrafish-specific computational ventricular action potential model presented here provides a valuable tool for studying cardiomyocyte electrophysiology using zebrafish", from which I understand your novel model is a single cell model (as your 'parent model' is). On the other hand, in the Abstract you also write: "The principal ionic current conductances in the model were calibrated and validated using new experimental data, including microelectrode measurements of membrane potential and optical measurements of intracellular calcium with steady-state and restitution pacing protocols." So, it appears that you have developed a single cell model using new experimental data from tissue, i.e., microelectrode measurements of membrane potential and optical measurements of intracellular calcium. However, a few lines before you were, as a Key Point, "highlighting the importance of tissue-based simulations for accurate comparison with tissue-derived experimental data". How should this be interpreted by an interested reader? Now that the '0D' single cell model is, at least partly, based on tissue data, what is the added value of running '1D' simulations?

2) In the Abstract you also write: "The model was then used to explore components underlying the zebrafish action potential and calcium transient, highlighting that the L-type calcium current plays a key role in the action potential upstroke and intracellular calcium transient and that both the rapid and slow delayed rectifier potassium currents make significant contributions to repolarisation." At the same time, as listed in Section 3.4 (entitled "Sensitivity Analysis"), "the only parameters that seem to not be greatly affected by ICaL are those related to Phase 0 (i.e., AP upstroke speed and time to peak). In contrast, ICaT primarily influences the speed of the AP upstroke." Thus, the T-type calcium current seems more important in the action potential upstroke than the L-type calcium current, as opposed to the claim in the Abstract. These contradictory statements require a more detailed explanation.

3) Lines 86-87: "reparametrised using published voltage-clamp data to adjust the biophysical properties of the principal ionic currents". This reads as if you completely ignore your own as yet unpublished patch-clamp data on ICaL that you used to construct your model. Actually, these single cell data are also ignored in your response, when you write: "Per our methods, all experiments conducted in this study were completed in whole explanted zebrafish hearts. While intracellular (microelectrode) recordings are single cell, this is done on the whole organ level. Doing these experiments in the whole heart allowed us to validate our tissue model." This apparent contradiction requires a reformulation of the abstract.

- Introduction

1) Lines 147-148: It is confusing to read "the Ten Tusscher and Panfilov formulation of the human ventricular AP (TP04; Ten Tusscher et al., 2004)". When reading "the Ten Tusscher and Panfilov formulation of the human ventricular AP", I expect that you refer to the Ten Tusscher and Panfilov (2006) reformulation of the Ten Tusscher et al. (2004) model rather than the Ten Tusscher et al. (2004) model. Consider rewording.

2) Lines 147-150: "For this, the Ten Tusscher and Panfilov formulation of the human ventricular

AP (TP04; Ten Tusscher et al., 2004) was modified by reparametrizing the biophysical properties of the principal ionic currents (fast Na+ current, INa); L- and T-type Ca2+ currents, ICaL and ICaT; (...)". This reads as if ICaT is present in the TP04 model, which it is not. Consider rewording.

3) Lines 136-162: You should clarify that "the developed model" is a '0D' model of a single isolated ventricular cardiomyocyte that can be used in a multicellular context, as you did with your '1D' simulations.

- Methods

1) Lines 166-168: "Patch-clamp experiments were approved by the Institutional Animal Care and Use Committee of Brigham and Women's Hospital and Harvard Medical School in accordance with local ethical guidelines." As a reader, I would accordingly expect that the patch-clamp experiments were carried out at the Brigham and Women's Hospital and Harvard Medical School. However, none of the authors seem to work, or have worked, at this institute (unlike the Dalhousie University, which is also mentioned in this novel section on Ethical Approval). How should I, as a reader, interpret this statement? This also holds for the "Institutional Ethics Committee" at the end of the manuscript (lines 1369-1374).

2) Section 2.2, "Experimental Preparation". What is the ratio behind the differences in the composition of the extracellular solution in experiments on isolated cells (Section 2.2.1) vs. whole hearts (Section 2.2.2)? How have these differences been accounted for?

3) Section 2.2.2, "ECG signals were recorded at 2 kHz". To which end? Can such ECG signal be shown as part of one of the figures?

4) Section 2.2.3, "Cm and series resistance were compensated for by at least 60%". It seems that this is the first appearance of "Cm". What does it denote? And what was its mean {plus minus} SD value?

5) Section 2.3, line 250, "a Boltzmann function". Is the "V" listed in the Boltzmann equation equivalent to the Vm listed elsewhere?

6) Section 2.5, "The computational model of the zebrafish ventricular AP developed in the current study was based on the TP04 formulation of the human ventricular AP (Ten Tusscher et al., 2004)". You included the T-type calcium current (ICaT), as it is observed in zebrafish ventricular cardiomyocytes rather than human ones, and left out the transient outward current (Ito), as it is observed in human ventricular cardiomyocytes rather than zebrafish ones. But what about the late sodium current (INaL) that has been observed in both human and zebrafish ventricular cardiomyocytes? Why is this current not included in the model? I realize that this current is not included in the TP04 'parent model' of human ventricular cardiomyocytes, but this parent model dates back to 2004. Actually, INaL is included in more recent models of human ventricular cardiomyocytes, like those by Tomek et al. (2019) and Bartolucci et al. (2020), which I already referred to in my initial review. The late sodium current of zebrafish ventricular myocytes was identified and functionally described by I. Dzhumaniiazova, M. Vornanen, O. B. Pustovit, Yu. I. Voronkov & D. V. Abramochkin in 2022 (DOI: 10.1134/S0022093022070031).

7) Section 2.5, lines 407-409: "This process was followed by manual parameter tuning, where the maximum conductance of the principal ionic currents was adjusted to better fit the model's different restitution characteristics." This is a rather vague description. A more precise description is required, so that future researchers can understand and repeat your approach.

8) Section 2.6, lines 388-391: "The effective diffusion coefficient was set to D = 1.76 · 10−2) mm2/ms, resulting in a conduction velocity of 126.8 mm/s, which is consistent with our experimental measurements (125 mm/s) and conduction velocity reported in the literature (Kikuchi et al., 2010; Wang et al., 2011)." Where can one find these "experimental measurements"? It seems that they are not part of the manuscript.

9) Section 2.6, lines 428-429: "Results were analysed in terms of the partial correlation of each model parameter with the

principal AP characteristics." Which are these "principal AP characteristics"? How did you compute this "partial correlation"? As above, a more precise description is required, so that future researchers can understand and repeat your approach.

10) Section 2.8, Figure 2. The font size of the legends as well as the size of the associated symbols is too low to be well appreciated by the reader. Both sizes should be increased. This does not only hold for Figure 2 but also for several of the subsequent figures.

11) Section 2.8, "Model Ionic Currents", lines 457-500. The time to peak and the activation time constant of the fast sodium current are presented as interchangeable in this (sub)section. To which extent is this actually true, both computationally and experimentally?

12) Section 2.8, "Model Ionic Currents". It seems that Figure 4 shows the newly obtained patch-clamp data on ICaL that are touched upon in Section 2.3 ("ICaL Measurements"). However, from Section 2.3 one learns that "the first pulse (300 ms with test potentials between −60 and + 70 mV) served to activate ICaL, from which densities and voltage-dependency of activation were analysed". These newly obtained patch-clamp data on ICaL are highly important, also outside the scope of creating a computer model, and deserve more attention, for example by showing typical current traces and extending the experimental data to the full range of test potentials. There is enough white space in Figure 4 to realize that.

13) Section 2.8, "Model Ionic Currents", lines 593-595: "The formulation for the IKs current in the model has been slightly modified from the original TP04 formulation by removing an activation gate to better fit the experimental data (...)". This may be somewhat confusing. It seems that there is still an activation gate, but that its kinetics are linear instead of quadratic.

14) Section 2.8, "Model Ionic Currents", lines 597-599: "EKs is a reversal potential determined by assuming a large permeability to K+ and a small permeability to Na+, as in the original TP04 model, (...)". The equations listed in Appendices 1 and 2 learn that this relative permeability amounts to 0.03. Yet, the "Intracellular Sodium and Potassium Dynamics" of lines 1129 and 1130 (as well as those of lines 641 and 644) treat IKs as if it is a pure K+ current, ignoring the Na+ component. This is an obvious discrepancy and should be repaired.

15) Section 2.8, "Model Ionic Currents", lines 601-604: "Steady-state activation, xS∞, and values of the activation gate time constant τxS were directly estimated from current recordings presented in the work of Abramochkin et al., 2018 (found in Figure 3 of the paper). Figure 6 shows the data and fits used for the model (see Appendix 1 for the detailed formulation)." The recordings of Abramochkin et al. (2018) were made at 25{degree sign}C, whereas the model temperature is 28{degree sign}C. It is unclear, from the body text as well as the appendix, how this difference was accounted for. There are also differences in sodium and potassium concentrations. This should be clarified. Throughout the manuscript, there are more instances at which it remains unclear how such differences in model vs. experimental conditions were accounted for. The manuscript should be checked for these instances and updated with clarifications.

16) Section 2.8, Figure 7. Physiologically, the fitted IK1 is only relevant in the voltage range from around −85 to +30 mV. It would therefore be helpful, using the white space that is available, to include an inset that shows IK1 on an enlarged scale limited to this voltage range.

- Results

1) Section 3.1, lines 673-675: "Figure 8 shows the comparison between the experimental AP recordings and the output of the calibrated 0D and 1D computational models during steady-state pacing at 2-Hz pacing." What is meant here by "calibrated 0D and 1D computational models"? Were the models adjusted for differences in ion concentrations or other parameters? Any changes should be specified. This also holds for the body text referring to Figure 9.

2) Figure 8. This figure demonstrates that your 0D and 1D simulation data agree well with your microelectrode recordings. However, it seems that you also have experimental AP recordings from single isolated myocytes at physiological temperature, which have been included in the paper by Koopman et al. (2021). How do your 0D simulation data agree with these patch clamp data? Why are these, or similar, single cell AP recordings not included in Figure 8?

3) Section 3.1, lines 752-755: "The current density of ICaL in the models, however, is higher than for human, with peak and plateau values almost double those found in recent human ventricular AP models (O'Hara et al., 2011), such that it will have a larger direct role in the CaT." Why do you only refer to O'Hara et al. (2011) when making a statement regarding "recent human ventricular AP models"? What about more recent human ventricular AP models?

4) Section 3.2, lines 766-768: "Figure 11 shows the restitution behaviour of various AP characteristics for the 0D and 1D computational models obtained with the S1-S2 pacing protocol, compared to averages of the experimental results". It would be valuable to specify, both here and in the legend to Figure 11, that these "experimental results" were obtained from whole hearts. A similar suggestion holds for Figures 12 and 13.

5) Section 3.4, lines 823-825: "Also different from what has been observed in human ventricular models (Romero et al., 2009), the Na+-K+ pump had a mild influence on AP repolarisation." What about more recent "human ventricular models", including the O'Hara et al. (2011) model that you are already referring to?

6) Section 3.4, lines 842-845: "In the model, the IKs:IK1 current density ratio is 1:5, while the IKs:IKr density is 1:12, in agreement with reports from the literature (Hassinen et al., 2015b, 2015a; Abramochkin et al., 2018). Yet, despite the relatively small density of IKs, it still has a large effect on repolarisation behaviour." The information regarding the two current density ratios is confusing. Which type of 'ratio' are you actually referring to? Apparently not to the current density ratios during the action potentials of Figure 10, in which these ratios are both near 1:1, so that it should not at all be surprising that IKs "still has a large effect on repolarisation behaviour". These lines require rephrasing.

7) Section 3.6, lines 904-908: "Table 4 shows the effect of simulated block of IKr (as with E4031) or IKs (as with chromanol 293B), or combined block of INa, IKr, and IKs (as with quinidine), on APD in the computational model. For block of IKr and IKs with E4031 or chromanol 293B, APD prolongation in the model fell within the experimental range previously reported for zebrafish (Tsai et al., 2011; Abramochkin et al., 2018; De la Cruz et al., 2023)." What exactly do you mean by "APD in the computational model"? Which APD (APD90 perhaps?) do you mean? And which is "the computational model"? 0D? 1D? And why are only the APD prolongation data by Tsai et al. (2011) listed in Table 4, while you also refer to those by Abramochkin et al. (2018) and by De la Cruz et al. (2023) in the body text?

8) Table 4, "Response to channel specific block." Please provide references for the amount of block used in the simulations (i.e. the "78% IKr", etc.) listed in the table. Check the concentrations of each of the blockers. It seems highly unlikely that these are "channel specific" at a concentration in the millimolar range, as listed in the table.

- Discussion

1) Lines 914-919: "Here we present the development of the first computational model of the zebrafish ventricular AP, including biophysical parameterisation of the principal ionic currents using patch-clamp data from the literature, as well as isolated cell (0D) and tissue (1D) model calibration and validation from newly generated microelectrode recordings of the AP and fluorescent recordings of the CaT during steady-state and restitution pacing protocols in the adult isolated heart." This suggests that you newly generated data are limited to microelectrode recordings in the adult isolated zebrafish heart. However, your manuscript also includes newly generated data obtained in patch-clamp experiments. These are important data and should also draw the attention of the reader.

2) Lines 923-924: "After model calibration using the steady-state pacing data, most AP characteristics were also within previously reported experimental ranges: (...)". But, perhaps even more importantly, which were not and how can that be

explained?

3) Lines 976-977: "(...) this concentration is similar to that reported in human (1.0· - 1.5· µM (Bers, 2000))." What is meant by the bullets in "1.0· - 1.5· µM"? Or should this simply read "1.0-1.5 µM"?

4) Lines 986-987: "It was further shown that IKs is one order of magnitude lower than IKr, the most prominent repolarising current in zebrafish (Vornanen & Hassinen, 2016)." See my comment above regarding Section 3.4, lines 842-845. It should be specified in which respect IKs is "one order of magnitude lower than IKr".

5) Lines 1005-1006: "However, upstroke speed and APA differed between the two, such that only the 1D simulations of restitution matched the experimental data." It should be specified which are "the experimental data".

6) Lines 1008-1009: "In the current study, all experimental measurements came from whole hearts, (...)." What, again, about the single cell data obtained in patch-clamp experiments?

7) Lines 1016-1018: "At a pacing rate of 2 Hz, after 100 s, intracellular Na+ stabilised at 7.99 mM, and intracellular K+ stabilised at 112.47 mM (see Figure A1 and Table A2 in Appendix 2)." But what if IKs is not treated as if it is a pure K+ current, thus not ignoring the Na+ component (see Section 2.8, "Model Ionic Currents", lines 597-599)?

- Appendix 2

From the values of the "Membrane capacitance" and "Capacitive area" listed in Appendix 2 one can compute that single zebrafish ventricular cardiomyocytes exhibit a membrane capacitance near 70 pF. Is this in line with your experimental data on such cardiomyocytes?

- References

Unfortunately, the list of references is still sloppy in several respects. For example, the newly added "Koopman CD et al. (2021)" should read "Koopman CD, De Angelis J, Iyer SP, Verkerk AO, Da Silva J, Berecki G, Jeanes A, Baillie GJ, Paterson S, Uribe V, Ehrlich OV, Robinson SD, Garric L, Petrou S, Simons C, Vetter I, Hogan BM, de Boer TP, Bakkers J, Smith KA (2021)". As another example, "Front Physiol; DOI: 10.3389/fphys.2018.01806" should be listed as "Front Physiol 9, 1806".

END OF COMMENTS

We thank the editors and reviewers for their additional suggestions on our manuscript, which have helped us to further improve our study. We hope our further revisions will now have addressed their concerns.

EDITOR COMMENTS

Reviewing Editor:

The revised version of your manuscript was reviewed by the two expert referees again and again significant number of issues remain that need to be addressed for further consideration of this manuscript, including:

- discrepancies between responses and data presented in the manuscript and between different parts of the manuscript. It is surprising that none of the 6 authors have identified these during the revision process;
- suboptimal reflection of the content of the manuscript in the title and key points;
- code not being available to the reviewers.

Please also see 'Required Items' above.

Senior Editor:

While somewhat improved, the revised manuscript still has major deficiencies, some of which had been raised in the first review.

We appreciate that Reviewer #2 still has concerns that need to be addressed related to their initial review, as summarised above, and hope that this revision will now adequately address those concerns. We would like to note, however, that many of the other issues raised by the reviewer in this round were not raised in their first review, so this is our first opportunity to respond to them. We hope the reviewer will not continue to find new issues with the manuscript going forward and instead focus on our responses to the concerns already raised.

In relation to the code not being available to the reviewer, in accordance with the Journal's guidelines, the 0D model will be provided in CellML format on www.cellml.org, and the MATLAB code for both the 0D and 1D models together with a copy of the CellML code will be available on GitHub (https://github.com/jfrodrig1971/Zebrafish). The link to the GitHub repository is included in the manuscript and will be made public once the paper has been accepted. A copy of the CellML code has been included in the submission as "Supporting information for review purposes only".

REFEREE COMMENTS

Referee #1:

I think this manuscript will be of great benefit to this research field, and my comments raised in the first round have all been addressed. The additional data included in the revised manuscript only further enhances the work.

We thank the reviewer for their kind words and for the previous review, which was of great value and helped us improve our work.

Referee #2:

We thank the reviewer for their time and effort in re-reviewing our manuscript and for their constructive and insightful comments and suggestions. Our response to each comment is provided below. Changes made to the manuscript are detailed here and appear in the revised manuscript as highlighted text.

GENERAL

A manuscript that describes a newly developed mathematical model should be unambiguous in all respects. A trained biomathematician should be able to program the model from the description of the model provided in the manuscript. This is still not the case, as detailed in my specific comments below.

We agree and hope our revision has satisfied this requirement.

In their response to my original comments, the authors stated that they "were referring to the maximal current amplitude during 2 Hz pacing" when they brought up an "I_{Ks} to I_{Kr} delayed rectifier potassium current ratio of ~1:12". Such statement regarding this ratio also appears in the revised manuscript ("In our computational model, the I_{Ks}:I_{Kr} density ratio is 1:12"). However, this is at odds with Figure 10, in which the current amplitude of both I_{Ks} and I_{Kr} is 1.0 µA/µF during 2 Hz pacing. An obvious discrepancy like this, which should have been noted by at least one of the six authors (all involved in review and editing, according to the "Authorship Contribution Statement" listed at the end of the manuscript), reduces my confidence in the quality of the manuscript.

We appreciate the reviewer's careful examination of our work and for pointing out this apparent discrepancy. Upon review, it became clear that imprecise wording in our previous description has created some confusion. When we stated that the I_{Ks}:I_{Kr} density ratio is 1:12, we were referring to the ratio of maximal conductances used in the model, rather than the steady-state current amplitudes during pacing. We have clarified the wording in the manuscript to make it clear that the 1:12 ratio pertains to the ratio of maximal conductances (G_{Ks}:G_{Kr}), rather than the current amplitudes during pacing, and hope this clears up the misunderstanding.

An important shortcoming of the model is that it does not include the late sodium current of zebrafish ventricular cardiomyocytes. This is an important current that has been linked to various ventricular arrhythmias in the human heart and is therefore important to be included in a model of zebrafish ventricular myocytes if regarded "a useful tool for future studies of cardiac electrophysiology and its dysfunction" (Discussion, last line).

While this issue was not raised in the initial review, we appreciate the comment and have addressed it in our detailed response below.

<u>*SPECIFIC*</u>

Title

1) The authors have changed the title from "A mathematical model of the zebrafish ventricular action potential from whole-heart voltage-calcium data" to "A mathematical model of the zebrafish ventricular action potential based on voltage and calcium measurements in the whole heart". The new title suggests that the model is purely based on voltage and calcium measurements in the whole heart (without measurements of the underlying ion currents). However, when going through the manuscript, one learns that the model is also based on patch-clamp data obtained from isolated zebrafish ventricular cardiomyocytes and "from zebrafish activation gates expressed in Chinese Hamster Ovary (CHO) cells." Actually, the patch-clamp data shown in Figure 4 seem to have been obtained as part of the present study. The title should be reworded accordingly.

We thank the reviewer for this suggestion. We have modified the title of the manuscript to the following one: "**A zebrafish ventricular action potential mathematical model integrating whole-heart voltage and calcium measurements and single-cell electrophysiology**". While it is true that we performed patch-clamp experiments to characterize the I_{CaL} current, data that were fundamental for deriving the steady-state activation and inactivation curves, as well as the τ_{fL} curve, all other ionic currents in the model were sourced from previously published literature. In contrast, the voltage and calcium measurements used for model validation were entirely obtained in-house from whole-heart experiments. We believe that referencing these whole-heart data in the title more accurately reflects the primary experimental contribution of this study and helps to avoid potential confusion regarding which patch-clamp data were newly generated and which were taken from existing sources.

Key Points

2) "We developed the first zebrafish-specific computational ventricular action potential model using newly generated whole-heart voltage-calcium experimental data." Which are these "whole-heart voltage-calcium experimental data"? Are these the "voltage and calcium measurements in the whole heart" of the updated title of the manuscript? If so, what about your newly generated experimental data on isolated cells?

We apologize for being unclear, but the reviewer is right. With "whole-heart voltage-calcium experimental data", we refer to voltage and calcium measurements obtained from the entire heart. While we also conducted patch-clamp experiments specifically for the I_{CaL} current – data that were crucial for deriving the steady-state activation and inactivation curves, as well as the τ_{fL} curve – all other ionic current data incorporated into the model were sourced from existing literature. In contrast, the voltage and calcium measurements from whole-heart experiments were entirely obtained in our laboratory and played a central role in the calibration and validation of our model. Therefore, we believed that highlighting this aspect in the key point was important. To more fully represent what we have done, we have updated this key point, which now reads:

"*We have developed the first zebrafish-specific computational ventricular action potential model, based on existing and new patch clamp data from single cells, with model calibration and validation performed using newly generated voltage and calcium measurements in the whole heart.*"

Abstract

3) At the bottom lines of the Abstract you have written: "Overall, the novel zebrafish-specific computational ventricular action potential model presented here provides a valuable tool for studying cardiomyocyte electrophysiology using zebrafish", from which I understand your novel model is a single cell model (as your 'parent model' is). On the other hand, in the Abstract you also write: "The principal ionic current conductances in the model were calibrated and

validated using new experimental data, including microelectrode measurements of membrane potential and optical measurements of intracellular calcium with steady-state and restitution pacing protocols." So, it appears that you have developed a single cell model using new experimental data from tissue, i.e., microelectrode measurements of membrane potential and optical measurements of intracellular calcium. However, a few lines before you were, as a Key Point, "highlighting the importance of tissue-based simulations for accurate comparison with tissue-derived experimental data". How should this be interpreted by an interested reader? Now that the '0D' single cell model is, at least partly, based on tissue data, what is the added value of running '1D' simulations?

We decided to replace the word "*cardiomyocyte*" with "*cardiac*" in the bottom lines of the Abstract, to avoid confusion regarding the development of both single-cell and tissue models. We used patch-clamp data to derive the steady-state and time-constant curves of the different gating variables. This formulation was used to develop both single-cell and tissue models, which were validated using tissue data. As shown in the Results section, this validation process clearly demonstrated that the 1D model better fits tissue data than the 0D model, as it accounts for the electrotonic coupling. Thus, as stated in the key points: "*While single-cell and tissue model simulations produced similar results, depolarisation-related parameters (i.e., action potential upstroke speed and amplitude) varied, highlighting the importance of tissue-based simulations for accurate comparison with tissue-derived experimental data.*"

4) In the Abstract you also write: "The model was then used to explore components underlying the zebrafish action potential and calcium transient, highlighting that the L-type calcium current plays a key role in the action potential upstroke and intracellular calcium transient and that both the rapid and slow delayed rectifier potassium currents make significant contributions to repolarisation." At the same time, as listed in Section 3.4 (entitled "Sensitivity Analysis"), "the only parameters that seem to not be greatly affected by I_{CaL} are those related to Phase 0 (i.e., AP upstroke speed and time to peak). In contrast, I_{CaT} primarily influences the speed of the AP upstroke." Thus, the T-type calcium current seems more important in the action potential upstroke than the L-type calcium current, as opposed to the claim in the Abstract. These contradictory statements require a more detailed explanation.

The reviewer is right; this wording was a mistake. We have updated the abstract to make the role of calcium currents more clear: I_{CaT} is crucial during the initial phase of the AP (*Phase 0*) for the upstroke, while I_{CaL} is important for sustaining the plateau phase (*Phase 2*).

5) Lines 86-87: "reparametrised using published voltage-clamp data to adjust the biophysical properties of the principal ionic currents". This reads as if you completely ignore your own as yet unpublished patch-clamp data on I_{CaL} that you used to construct your model. Actually, these single cell data are also ignored in your response, when you write: "*Per our methods, all experiments conducted in this study were completed in whole explanted zebrafish hearts. While intracellular (microelectrode) recordings are single cell, this is done on the whole organ level. Doing these experiments in the whole heart allowed us to validate our tissue model.*" This apparent contradiction requires a reformulation of the abstract.

We agree, our previous wording did not adequately reflect the incorporation of our own patch-clamp data for I_{CaL} into the model. We have revised the abstract to explicitly state that the model incorporates both published voltage-clamp data and our own patch-clamp recordings.

Introduction

6) Lines 147-148: It is confusing to read "the Ten Tusscher and Panfilov formulation of the human ventricular AP (TP04; Ten Tusscher et al., 2004)". When reading "the Ten Tusscher and Panfilov formulation of the human ventricular AP", I expect that you refer to the Ten Tusscher and Panfilov (2006) reformulation of the Ten Tusscher et al. (2004) model rather than the Ten Tusscher et al. (2004) model. Consider rewording.

Thank you for the comment; we have revised the text to make this more clear.

7) Lines 147-150: "For this, the Ten Tusscher and Panfilov formulation of the human ventricular AP (TP04; Ten Tusscher et al., 2004) was modified by reparametrizing the biophysical properties of the principal ionic currents (fast Na^+ current, I_{Na}); L- and T-type Ca^{2+} currents, I_{CaL} and I_{CaT}; (...)". This reads as if I_{CaT} is present in the TP04 model, which it is not. Consider rewording.

The reviewer is right; we have rephrased this sentence.

8) Lines 136-162: You should clarify that "the developed model" is a '0D' model of a single isolated ventricular cardiomyocyte that can be used in a multicellular context, as you did with your '1D' simulations.

We thank the reviewer for this suggestion. We agree that it is important to clearly distinguish that the developed model is a 0D single-cell model, which can be incorporated into a multicellular framework, as demonstrated in our 1D

simulations. To address this, we have revised the text to explicitly state that our model represents a 0D isolated ventricular cardiomyocyte, with the capability to be used in multicellular contexts.

Methods

9) Lines 166-168: "Patch-clamp experiments were approved by the Institutional Animal Care and Use Committee of Brigham and Women's Hospital and Harvard Medical School in accordance with local ethical guidelines." As a reader, I would accordingly expect that the patch-clamp experiments were carried out at the Brigham and Women's Hospital and Harvard Medical School. However, none of the authors seem to work, or have worked, at this institute (unlike the Dalhousie University, which is also mentioned in this novel section on Ethical Approval). How should I, as a reader, interpret this statement? This also holds for the "Institutional Ethics Committee" at the end of the manuscript (lines 1369-1374).

We thank the reviewer for pointing out this issue, and we understand the confusion. Indeed, none of the co-authors is working at the mentioned US institutions. The fish used in Amsterdam for the patch clamp studies were originally sent from Harvard for the study of Chiang *et al.* (2024). Upon arrival in the Netherlands, the fish were kept in the quarantine room of the Hubrecht Institute in Utrecht for several weeks. On the day of cell isolation, fish were transported to Amsterdam. Since the cell isolations for that study resulted in an excess of available cells, and the experiments were quick, the additional cells were used for I_{CaL} measurements for the current study (performed by Dr. Arie Verkerk). In the previous version of our manuscript, we neglected to report the relevant approval for the patch clamp experiments, which came from the Dutch Central Authority for Scientific Procedures on Animals, following the guidelines of the and the Utrecht and Amsterdam animal welfare commissions. We have rewritten the ‘2.1 Ethical Approval’ section, so that it now includes the relevant information; we apologise for not including the correct information in the first instance. Please note that we have also modified section ‘2.2.1 Cell Preparation’ to include relevant information regarding the experiments. Regarding the statement ‘Institutional Ethics Committee’ at the end of the manuscript, this has also been updated.

10) Section 2.2, "Experimental Preparation". What is the ratio behind the differences in the composition of the extracellular solution in experiments on isolated cells (Section 2.2.1) vs. whole hearts (Section 2.2.2)? How have these differences been accounted for?

The differences in the composition of the extracellular solutions used in experiments on isolated cells versus whole hearts reflect different requirements for experiments performed in these two distinct experimental preparations and are in accordance with the standard practices of the two laboratories. We acknowledge that variations in extracellular ionic composition potentially influence electrophysiological properties, however this is common to all experimental work performed in different experimental preparations and across labs and is not unique to our study. Addressing the effects of these differences is a much bigger issue that is well beyond the scope of the current study. Please note that key parameters (such as the $CaCl_2$ concentration of 1.8 mM) are the same in both solutions, such that critical biophysiological properties of I_{CaL} were hopefully not affected.

11) Section 2.2.2, "ECG signals were recorded at 2 kHz". To which end? Can such ECG signal be shown as part of one of the figures?

The ECG signals were used exclusively as event markers to confirm proper pacing capture throughout the experiments and were not intended to serve as primary data for analysis. This is now indicated in the text, and accordingly, we have decided not to include a representative ECG trace in the figures.

12) Section 2.2.3, "C_m and series resistance were compensated for by at least 60%". It seems that this is the first appearance of "C_m". What does it denote? And what was its mean ± SD value?

“C_m” stands for cell membrane capacitance, and it is defined at its first usage in the previous sentence. The C_m was 27.2 ± 8.8 pF (mean ± SD), which is now included in the text (line 247).

13) Section 2.3, line 250, "a Boltzmann function". Is the "V" listed in the Boltzmann equation equivalent to the V_m listed elsewhere?

It is. This has been amended.

14) Section 2.5, "The computational model of the zebrafish ventricular AP developed in the current study was based on the TP04 formulation of the human ventricular AP (Ten Tusscher et al., 2004)". You included the T-type calcium current (I_{CaT}), as it is observed in zebrafish ventricular cardiomyocytes rather than human ones and left out the transient outward current (I_{to}), as it is observed in human ventricular cardiomyocytes rather than zebrafish ones. But what about the late sodium current (I_{NaL}) that has been observed in both human and zebrafish ventricular cardiomyocytes? Why is

this current not included in the model? I realize that this current is not included in the TP04 'parent model' of human ventricular cardiomyocytes, but this parent model dates back to 2004. Actually, I_{NaL} is included in more recent models of human ventricular cardiomyocytes, like those by Tomek et al. (2019) and Bartolucci et al. (2020), which I already referred to in my initial review. The late sodium current of zebrafish ventricular myocytes was identified and functionally described by I. Dzhumaniiazova, M. Vornanen, O. B. Pustovit, Yu. I. Voronkov & D. V. Abramochkin in 2022 (DOI: 10.1134/S0022093022070031).

We appreciate the reviewer's interest in having the late sodium current (I_{NaL}) included in our model. While indeed, the paper by Dzhumaniiazova et al. (2022) reports I_{NaL} in zebrafish ventricular myocytes, and we could have added this current to our model as we did for the T-type calcium current, we chose not to for specific reasons.

Firstly, the functional role of I_{NaL} in zebrafish under physiological conditions remains poorly understood. In this regard, the study by Dzhumaniiazova et al. does not provide sufficient information to create a proper mathematical formulation for this current. They present only the dose-dependent effects of TTX and Ranolazine on the fast sodium current (I_{NaF}) and I_{NaL}, and use this to report a $Peak_{INaL}/Peak_{INaF}$ ratio of ~1% (a value consistent with that reported for human cardiomyocytes; *e.g.*, see Maltsev et al., 1998, doi: 10.1161/01.CIR.98.23.2545). However, the $Peak_{INaL}/Peak_{INaF}$ ratio of Dzhumaniiazova et al. may be an overestimation, since they recorded $Peak_{INaF}$ at -100 mV, a potential at which Na^+ channels are largely inactive (Haverinen et al., 2018). Moreover, there were most likely differences in the driving force for $Peak_{INaL}$ and $Peak_{INaF}$ in their measurements, as $PeakI_{NaL}$ was not measured at a fixed voltage.

Furthermore, Dzhumaniiazova et al. provide only a representative I–V curve for I_{NaL} (Figure 1b in the manuscript), obtained using a depolarizing voltage ramp protocol. The I–V curve indicates that the current is present only between – 60 and – 40 mV (or – 70 mV to – 40 mV, according to the text on page S25). This range is not only much narrower than that reported for human cardiomyocytes (– 60 to + 60 mV, as in Maltsev et al., 1998, doi: 10.1161/01.CIR.98.23.2545), but also significantly narrower than the activation range of I_{NaF} in zebrafish, which spans from – 60 to + 40 mV (Haverinen et al., 2021, doi: 10.1242/jeb.241067). Importantly, the depolarizing RAMP used in the study by Dzhumaniiazova et al. typically results in a smaller voltage range of I_{Na} activation than a repolarizing RAMP, such that a depolarizing RAMP represents the I_{Na} window current (Swan et al., 2014), rather than I_{NaL}.

Ultimately, the findings of Dzhumaniiazova et al. suggest that the contribution of I_{NaL} to the zebrafish action potential is likely to be small, if not negligible, and mostly confined to the depolarization phase, with minimal influence on the plateau phase. Therefore, including it in the current model would add complexity without providing a commensurate benefit in terms of model accuracy or predictive capability.

We acknowledge that I_{NaL} may play a more significant role under certain pathological conditions or in response to specific pharmacological agents. As such, we consider the incorporation of I_{NaL} as a potential avenue for future model refinement when more comprehensive data on this current becomes available.

15) Section 2.5, lines 407-409: "This process was followed by manual parameter tuning, where the maximum conductance of the principal ionic currents was adjusted to better fit the model's different restitution characteristics." This is a rather vague description. A more precise description is required, so that future researchers can understand and repeat your approach.

We apologize if the procedure was not clear. The first step of the work involved reformulation of the main currents in the model for which patch-clamp data were available in the literature or obtained by us (the L-type calcium current). With this reformulation, we performed a Monte Carlo analysis to assess the model's stability. In particular, we evaluated which combinations of maximal current conductances and Ca^{2+} dynamics parameters did not result in the emergence of alternants or automaticity and produced action potentials and calcium transients with features matching our experimental measurements. The model turned out to be very stable, with a large number of viable parameter combinations. We then manually tuned the G_{max} values to select the model that visually best represented the experimental results. The final conductance values are the ones reported in Table A1 in Appendix 2. This has now been clarified in lines 429-436. From this point, we performed a further sensitivity analysis to study the influence of the currents on the action potential.

As shown in the figure below, this final analysis also confirms the model's stability, allowing for variations in parameters, while still remaining within experimental ranges.

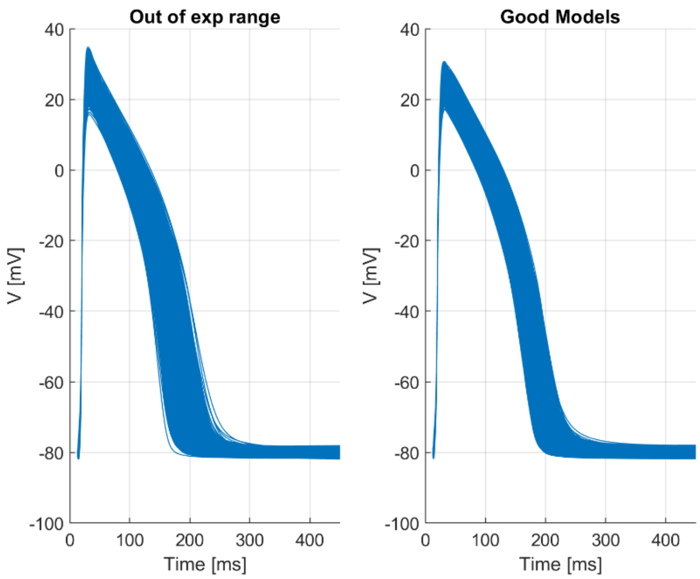

16) Section 2.6, lines 388-391: "The effective diffusion coefficient was set to D = 1.76 · 10−2) mm2/ms, resulting in a conduction velocity of 126.8 mm/s, which is consistent with our experimental measurements (125 mm/s) and conduction velocity reported in the literature (Kikuchi et al., 2010; Wang et al., 2011)." Where can one find these "experimental measurements"? It seems that they are not part of the manuscript.

The experimental conduction velocity measurements were obtained as part of our whole heart restitution experiments and served as an important validation of our model's predicted conduction velocity. We have added a few lines to the text to explain this.

17) Section 2.6, lines 428-429: "Results were analysed in terms of the partial correlation of each model parameter with the principal AP characteristics." Which are these "principal AP characteristics"? How did you compute this "partial correlation"? As above, a more precise description is required, so that future researchers can understand and repeat your approach.

The "principal AP characteristics" refer to RMP, APA, $APD_{20\text{-}90}$, dV/dt_{max}, V_{max}, t_{peak}, and AP triangulation. To quantify the unique relationship between each model parameter and these AP characteristics, we computed partial correlation coefficients using standard linear partial correlation analysis. In this approach, for each AP characteristic, we controlled for the influence of the other model parameters via a regression-based method to isolate the direct association between the parameter of interest and the AP characteristic. This yields a partial correlation coefficient that reflects the independent contribution of each parameter, along with corresponding significance metrics. We have included these details in the revised manuscript.

18) Section 2.8, Figure 2. The font size of the legends as well as the size of the associated symbols is too low to be well appreciated by the reader. Both sizes should be increased. This does not only hold for Figure 2 but also for several of the subsequent figures.

We thank the reviewer for this suggestion, however, as the figures will be available in full resolution and size in the final submission and the online version, we believe that the font sizes and symbols are adequate for clear interpretation. That said, we will remain open to adjustments if during the production process it becomes clear that modifications are needed for optimal readability.

19) Section 2.8, "Model Ionic Currents", lines 457-500. The time to peak and the activation time constant of the fast sodium current are presented as interchangeable in this (sub)section. To which extent is this actually true, both computationally and experimentally?

We thank the reviewer for pointing out the ambiguity in our description. To clarify, the activation time constant of the fast sodium current in our model was derived from the experimental time-to-peak data. We have now revised the manuscript to clearly differentiate between these two measures and to explain that the experimental time-to-peak data were used to inform the determination of the activation time constant, rather than treating them as interchangeable.

20) Section 2.8, "Model Ionic Currents". It seems that Figure 4 shows the newly obtained patch-clamp data on I_{CaL} that are touched upon in Section 2.3 ("ICaL Measurements"). However, from Section 2.3 one learns that "the first pulse (300

ms with test potentials between − 60 and + 70 mV) served to activate I_{CaL}, from which densities and voltage-dependency of activation were analysed". These newly obtained patch-clamp data on I_{CaL} are highly important, also outside the scope of creating a computer model, and deserve more attention, for example by showing typical current traces and extending the experimental data to the full range of test potentials. There is enough white space in Figure 4 to realize that.

We agree with the reviewer and have now included a new panel in the figure (Panel A) with the voltage clamp protocol as well as a typical current trace of I_{CaL}. Furthermore, we have indicated how some of the parameters were analysed. The Methods and Results sections have also been updated to describe and to refer to the new panel in Figure 4.

21) Section 2.8, "Model Ionic Currents", lines 593-595: "The formulation for the I_{Ks} current in the model has been slightly modified from the original TP04 formulation by removing an activation gate to better fit the experimental data (...)". This may be somewhat confusing. It seems that there is still an activation gate, but that its kinetics are linear instead of quadratic.

We apologize for the confusion. Yes, the formulation of the I_{Ks} current still includes an activation gate, and indeed, in the original TP04 model the open probability of I_{Ks} is governed by a squared activation gate (x_s^2), while in our model we reduced this to a linear form (x_s) to better match the experimental data. We have revised the text to clarify that the number of gates has not been reduced, but rather the activation kinetics have been simplified from a quadratic to a linear form.

22) Section 2.8, "Model Ionic Currents", lines 597-599: "EKs is a reversal potential determined by assuming a large permeability to K+ and a small permeability to Na+, as in the original TP04 model, (...)". The equations listed in Appendices 1 and 2 learn that this relative permeability amounts to 0.03. Yet, the "Intracellular Sodium and Potassium Dynamics" of lines 1129 and 1130 (as well as those of lines 641 and 644) treat IKs as if it is a pure K+ current, ignoring the Na+ component. This is an obvious discrepancy and should be repaired.

We appreciate the reviewer's observation regarding the treatment of I_{Ks} in our model. As noted, both the reversal potential for I_{Ks} (E_{Ks}) and the equations governing intracellular sodium and potassium dynamics are unchanged from the original TP04 model. In the TP04 model, as in many other AP models, I_{Ks} is modelled as a mixed K^+/Na^+ current, with a relative sodium permeability of 0.03. However, the intracellular sodium and potassium dynamics equations treat I_{Ks} as a pure K^+ current, effectively ignoring the small Na^+ component. This simplification is consistent with the original TP04 model and is based on the rationale that the sodium permeability of I_{Ks} is minimal and has a negligible effect on intracellular sodium concentration dynamics. While this approach introduces a minor inconsistency, it is a common modelling practice aimed at reducing complexity without significantly compromising accuracy.

For instance, this approximation is adopted in several models including O'Hara et al. (2011) and Tomek et al. (2019), which formulate I_{Ks} as follows:

$$I_{Ks} = GKs \cdot KsCa \cdot xs1 \cdot xs2 \cdot (V_m - E_{Ks}),$$

with $E_{Ks} = \frac{R \cdot T}{zk \cdot F} \ln \frac{[K^+]_o + pKNa[Na^+]_o}{[K^+]_i + pKNa[Na^+]_i}$, where $pKNa = 0.0183$, yet then treat I_{Ks} as a pure K^+ current for the intracellular sodium and potassium dynamics equations as in TP04, and as we do in our model.

23) Section 2.8, "Model Ionic Currents", lines 601-604: "Steady-state activation, $x_{S\infty}$, and values of the activation gate time constant τ_{xS} were directly estimated from current recordings presented in the work of Abramochkin et al., 2018 (found in Figure 3 of the paper). Figure 6 shows the data and fits used for the model (see Appendix 1 for the detailed formulation)." The recordings of Abramochkin et al. (2018) were made at 25 °C, whereas the model temperature is 28 °C. It is unclear, from the body text as well as the appendix, how this difference was accounted for. There are also differences in sodium and potassium concentrations. This should be clarified. Throughout the manuscript, there are more instances at which it remains unclear how such differences in model vs. experimental conditions were accounted for. The manuscript should be checked for these instances and updated with clarifications.

We thank the reviewer for this comment. We acknowledge that the temperature and ionic conditions under which the experimental data were collected (e.g., 25°C in Abramochkin et al., 2018) differ from those used in our model simulations (28°C). In the specific case of the activation kinetics derived from Abramochkin et al. (2018), we did not apply a formal Q_{10}-based correction to adjust the time constants for temperature differences between 25°C and 28°C, as temperature-dependent scaling factors for the kinetics of the slow delayed rectifier potassium current (I_{Ks}) in zebrafish are not available in the literature. Applying a generic Q_{10} value without species- and current-specific validation could introduce greater inaccuracies. Moreover, this approach aligns with common practices in prior modelling studies that rely on published data without full physiological matching.

Regarding ionic concentrations, we recognize that variations in extracellular and intracellular ion concentrations can affect action potential properties. However, in the context of our model, we maintained consistent ionic concentrations to match the conditions under which the whole-heart experimental data were obtained. This decision was made to ensure the validity of the parameterization and to avoid introducing additional variables that could complicate the interpretation of the results.

24) Section 2.8, Figure 7. Physiologically, the fitted I_{K1} is only relevant in the voltage range from around −85 to +30 mV. It would therefore be helpful, using the white space that is available, to include an inset that shows I_{K1} on an enlarged scale limited to this voltage range.

We thank the reviewer for this constructive suggestion. We agree that an inset highlighting the physiologically relevant voltage range for I_{K1} (from approximately – 90 to + 30 mV) would enhance the clarity of Figure 7. This is now included in the revised manuscript.

Results

25) Section 3.1, lines 673-675: "Figure 8 shows the comparison between the experimental AP recordings and the output of the calibrated 0D and 1D computational models during steady-state pacing at 2-Hz pacing." What is meant here by "calibrated 0D and 1D computational models"? Were the models adjusted for differences in ion concentrations or other parameters? Any changes should be specified. This also holds for the body text referring to Figure 9.

By "*calibrated*", we meant that the models were obtained after a careful tuning of the ionic current conductances, as explained in more detail above . In any case, we agree that the term may be confusing, so it has been removed.

26) Figure 8. This figure demonstrates that your 0D and 1D simulation data agree well with your microelectrode recordings. However, it seems that you also have experimental AP recordings from single isolated myocytes at physiological temperature, which have been included in the paper by Koopman et al. (2021). How do your 0D simulation data agree with these patch clamp data? Why are these, or similar, single cell AP recordings not included in Figure 8?

In modern AP models, such as O'Hara et al. (2011) and Tomek et al. (2019), while single currents kinetics are adjusted based on single-cell patch clamp experiments, microelectrode AP recordings from ventricular tissue preparations at physiological temperature are used to validate AP characteristics (for instance, see Figure 6 in O'Hara et al, 2011doi:10.1371/journal.pcbi.1002061.g006). We have adopted the same procedure in our model. Microelectrode measurements of AP from tissue preparations are believed to be more reliable than those obtained in single cell, as as cell isolation is a disruptive and potentially damaging process that can compromise the density and function of ionic channels in the sarcolemma. For this reason, we chose not to include AP recordings from single isolated myocytes and instead focus on our tissue measurements.

27) Section 3.1, lines 752-755: "The current density of I_{CaL} in the models, however, is higher than for human, with peak and plateau values almost double those found in recent human ventricular AP models (O'Hara et al., 2011), such that it will have a larger direct role in the CaT." Why do you only refer to O'Hara et al. (2011) when making a statement regarding "recent human ventricular AP models"? What about more recent human ventricular AP models?

We thank the reviewer for this observation. In fact, both the Tomek "ToR-ORd" model (Tomek et al., 2019) and the Bartolucci model (Bartolucci et al., 2020) are built directly on the O'Hara–Rudy dynamic (ORd) framework and retain its original maximal conductance for the L-type calcium current. As a result, the peak and plateau I_{CaL} densities are identical across these three models. Therefore, our reference to O'Hara et al. (2011) appropriately represents the I_{CaL} current density in contemporary human ventricular AP models. However, for the sake of clarity, we have added reference to both the Tomek and Bartolucci ventricular models.

28) Section 3.2, lines 766-768: "Figure 11 shows the restitution behaviour of various AP characteristics for the 0D and 1D computational models obtained with the S1-S2 pacing protocol, compared to averages of the experimental results". It would be valuable to specify, both here and in the legend to Figure 11, that these "experimental results" were obtained from whole hearts. A similar suggestion holds for Figures 12 and 13.

We thank the reviewer for this valuable suggestion. We have revised the manuscript to clarify that the experimental results presented in Section 3.2 and in Figures 11, 12, and 13 were obtained from whole heart experiments.

29) Section 3.4, lines 823-825: "Also different from what has been observed in human ventricular models (Romero et

al., 2009), the Na^+-K^+ pump had a mild influence on AP repolarisation." What about more recent "human ventricular models", including the O'Hara et al. (2011) model that you are already referring to?

The decision to cite the work by Romero et al. (2009) was motivated by the fact that in that study the impact of ionic currents was tested in the Ten Tusscher model, which is our parent model. Moreover, in that study the influence of ionic current variability was assessed through partial correlation analysis, as in our work. The text now makes this point clear.

30) Section 3.4, lines 842-845: "In the model, the I_{Ks}:I_{K1} current density ratio is 1:5, while the I_{Ks}:I_{Kr} density is 1:12, in agreement with reports from the literature (Hassinen et al., 2015b, 2015a; Abramochkin et al., 2018). Yet, despite the relatively small density of I_{Ks}, it still has a large effect on repolarisation behaviour." The information regarding the two current density ratios is confusing. Which type of 'ratio' are you actually referring to? Apparently not to the current density ratios during the action potentials of Figure 10, in which these ratios are both near 1:1, so that it should not at all be surprising that I_{Ks} "still has a large effect on repolarisation behaviour". These lines require rephrasing.

We apologize for the confusion. As mentioned above, the ratios mentioned in Section 3.4 (i.e. I_{Ks}:I_{K1} of 1:5 and I_{Ks}:I_{Kr} of 1:12) refer to the maximal conductance values used in our ionic current formulations, not to the instantaneous current densities observed during the action potential (as shown in Figure 10). We have revised the text to clearly indicate that these ratios reflect the parameterisation of maximum conductances, rather than the dynamic current density ratios.

31) Section 3.6, lines 904-908: "Table 4 shows the effect of simulated block of I_{Kr} (as with E4031) or I_{Ks} (as with chromanol 293B), or combined block of I_{Na}, I_{Kr}, and I_{Ks} (as with quinidine), on APD in the computational model. For block of I_{Kr} and I_{Ks} with E4031 or chromanol 293B, APD prolongation in the model fell within the experimental range previously reported for zebrafish (Tsai et al., 2011; Abramochkin et al., 2018; De la Cruz et al., 2023)." What exactly do you mean by "APD in the computational model"? Which APD (APD_{90} perhaps?) do you mean? And which is "the computational model"? 0D? 1D? And why are only the APD prolongation data by Tsai et al. (2011) listed in Table 4, while you also refer to those by Abramochkin et al. (2018) and by De la Cruz et al. (2023) in the body text?

The work by Tsai et al. (2011), along with that of Crumb et al. (2016; for quinidine), was used to set the percentage of channel block, and therefore allow for a direct comparison of results (the values provided in the table). In the other cases, since drug concentration conditions or percentage block were not comparable, a quantitative comparison was not possible – only a qualitative one (hence the choice to cite those references in the text rather than in the table). In the manuscript, "APD in the computational model" refers specifically to the action potential duration measured at 90% repolarisation (APD_{90}) within the one-dimensional (1D) tissue model. We have modified Table 4 to make this clear.

32) Table 4, "Response to channel specific block." Please provide references for the amount of block used in the simulations (i.e. the "78% I_{Kr}", etc.) listed in the table. Check the concentrations of each of the blockers. It seems highly unlikely that these are "channel specific" at a concentration in the millimolar range, as listed in the table.

There was a mistake in the table, which indicated mM instead of µM, which has been corrected. As for the percentage of block, it was computed using Equation 4, and as stated in Section 2.9, the other parameters are from the work by Tsai et al. (2011) and Crumb et al. (2016).

Discussion

33) Lines 914-919: "Here we present the development of the first computational model of the zebrafish ventricular AP, including biophysical parameterisation of the principal ionic currents using patch-clamp data from the literature, as well as isolated cell (0D) and tissue (1D) model calibration and validation from newly generated microelectrode recordings of the AP and fluorescent recordings of the CaT during steady-state and restitution pacing protocols in the adult isolated heart." This suggests that you newly generated data are limited to microelectrode recordings in the adult isolated zebrafish heart. However, your manuscript also includes newly generated data obtained in patch-clamp experiments. These are important data and should also draw the attention of the reader.

We thank the reviewer for this comment. We agree that we should also highlight our newly generated patch-clamp data, so this deserves explicit emphasis. We have revised the manuscript accordingly.

34) Lines 923-924: "After model calibration using the steady-state pacing data, most AP characteristics were also within previously reported experimental ranges: (...)". But, perhaps even more importantly, which were not and how can that be explained?

We apologise; this statement was an error held over from a previous revision. With recent updates to the models, all AP features are within the experimental data. We have corrected the text.

35) Lines 976-977: "(...) this concentration is similar to that reported in human (1.0· - 1.5· μM (Bers, 2000))." What is meant by the bullets in "1.0· - 1.5· μM"? Or should this simply read "1.0-1.5 μM"?

Apologies, these were added in error; this has now been amended.

36) Lines 986-987: "It was further shown that I_{Ks} is one order of magnitude lower than I_{Kr}, the most prominent repolarising current in zebrafish (Vornanen & Hassinen, 2016)." See my comment above regarding Section 3.4, lines 842-845. It should be specified in which respect IKs is "one order of magnitude lower than I_{Kr}".

In our manuscript, we noted that I_{Ks} is approximately one order of magnitude lower than I_{Kr}, referencing Vornanen & Hassinen (2016). This comparison pertains to the maximal conductance values of these currents in zebrafish ventricular myocytes. Specifically, the maximal conductance of I_{Ks} is about ten times lower than that of I_{Kr}, indicating a significantly smaller contribution of I_{Ks} to the overall repolarizing current. We have revised the manuscript to explicitly state that this comparison refers to the maximal conductance values.

37) Lines 1005-1006: "However, upstroke speed and APA differed between the two, such that only the 1D simulations of restitution matched the experimental data." It should be specified which are "the experimental data".

This has now been clarified.

38) Lines 1008-1009: "In the current study, all experimental measurements came from whole hearts, (...)." What, again, about the single cell data obtained in patch-clamp experiments?

This has now been corrected.

39) Lines 1016-1018: "At a pacing rate of 2 Hz, after 100 s, intracellular Na^+ stabilised at 7.99 mM, and intracellular K^+ stabilised at 112.47 mM (see Figure A1 and Table A2 in Appendix 2)." But what if I_{Ks} is not treated as if it is a pure K^+ current, thus not ignoring the Na^+ component (see Section 2.8, "Model Ionic Currents", lines 597-599)?

Our model does not treat I_{Ks} as a pure K^+ current in terms of reversal potential; it incorporates a small Na^+ permeability, as described in Section 2.8. However, consistent with the original TP04 formulation and several modern AP models (e.g., O'Hara et al., 2011; Tomek et al., 2019), the contribution of Na^+ through I_{Ks} is indeed not included in the intracellular Na^+ and K^+ balance equations. This simplification is justified by the minimal impact of the Na^+ component of I_{Ks} and is a common practice, so the impact of Na^+ conductance via I_{Ks} on the stability of the intracellular ion concentrations can be ignored.

Appendix 2

40) From the values of the "Membrane capacitance" and "Capacitive area" listed in Appendix 2 one can compute that single zebrafish ventricular cardiomyocytes exhibit a membrane capacitance near 70 pF. Is this in line with your experimental data on such cardiomyocytes?

The membrane capacitance we have measured using patch clamp in the current study is 27.2 ± 8.8 pF, which is now included in section 2.2.3. This value is close to that reported in Nemtsas et al (2010) (30.6±1.5 pF), and to values measured previously by Dr. Verkerk in WT zebrafish ventricular CMs in the studies of Koopman et al., 2021 (23.3 ± 5.2 pF), Kamel et al., 2021 (22.3 ± 3.9 pF), and Chiang et al., 2024 (21.6 ± 5.6 pF). Thus, ventricular zebrafish cardiomyocytes have a relatively low membrane capacitance, which can be explained by their narrow morphology, as reviewed previously (See Verkerk and Ramme et al., 2012).

We thank the reviewer for the observation regarding membrane capacitance. To address this discrepancy, we revisited the work by Brette et al. (2008), one of the few studies reporting morphological characteristics of zebrafish myocytes. In Brette's study, cell volume ranges from 900 to 4200 μm^3, while cell surface area varies between 1016 and 2625 μm^2. By considering the lower bound values—i.e., 975 μm^3 for cell volume and 1500 μm^2 for cell surface area—and assuming that the cytoplasmic volume accounts for 40% of the cell volume and that the capacitive area is twice the cell surface area, we obtain a surface-to-volume ratio of 7.7, which aligns with the current model settings.

Accordingly, we have updated the following model parameters in Appendix 2:

- Capacitive surface area (Ac): 3000 μm^2
- Cytoplasmic volume (Vc): 390 μm^3

These parameters yield a membrane capacitance of 30 pF, which falls within the range of experimental values.

References

41) Unfortunately, the list of references is still sloppy in several respects. For example, the newly added "Koopman CD et al. (2021)" should read "Koopman CD, De Angelis J, Iyer SP, Verkerk AO, Da Silva J, Berecki G, Jeanes A, Baillie GJ, Paterson S, Uribe V, Ehrlich OV, Robinson SD, Garric L, Petrou S, Simons C, Vetter I, Hogan BM, de Boer TP, Bakkers J, Smith KA (2021)". As another example, "Front Physiol; DOI: 10.3389/fphys.2018.01806" should be listed as "Front Physiol 9, 1806".

We thank the reviewer for pointing to this issue. We have followed the Journal's reference style and have corrected any mistakes in the revised version of the manuscript.

Dear Dr Cestariolo,

Re: JP-RP-2025-287624R2 "A Zebrafish Ventricular Action Potential Mathematical Model Integrating Whole-Heart Voltage and Calcium Measurements and Single-Cell Electrophysiology" by Ludovica Cestariolo, Zachary David Long, Arie O Verkerk, Jose Maria Ferrero, T Alexander Quinn, and Jose Felix Rodriguez Matas

Thank you for submitting your manuscript to The Journal of Physiology. It has been assessed by a Reviewing Editor and by 1 expert referee. You are now invited to respond to the comments of the reviewers and submit a further revised version for consideration.
The referee reports are copied at the end of this email.

Please address all the points raised and incorporate all requested revisions or explain in your Response to Referees why a change has not been made. We hope you will find the comments helpful and that you will be able to return your revised manuscript within 4 weeks. If you require longer than this, please contact journal staff: jp@physoc.org.

Your revised manuscript should be submitted online using the link in your Author Tasks Link Not Available. This link is accessible via your account as Corresponding Author; it is not available to your co-authors. If this presents a problem, please contact journal staff (jp@physoc.org). Image files from the previous version are retained on the system. Please ensure you replace or remove any files that are being revised.

If you do not wish to submit a revised version of your manuscript, you must inform our journal staff (jp@physoc.org) or reply to this email to request withdrawal. Please note that a manuscript must be formally withdrawn from the peer review process at one journal before it may be submitted to another journal.

TRANSPARENT PEER REVIEW POLICY: To improve the transparency of its peer review process The Journal of Physiology publishes online, as supporting information, the peer review history of all articles accepted for publication. Readers will have access to decision letters, including Editors' comments and referee reports, for each version of the manuscript, as well as any author responses to peer review comments. Referees can decide whether or not they wish to be named on the peer review history document.

ABSTRACT FIGURES: Authors are expected to use The Journal's premium BioRender account to create/redraw their Abstract Figures. Information on how to access this account is here: https://physoc.onlinelibrary.wiley.com/journal/14697793/biorender-access.

This will enable Authors to create and download high-resolution figures. If authors have used the free BioRender service, they can use the instructions provided in the link above to download a high-resolution version suitable for publication.
The link provided should only be used for the purposes of this submission. Authors will be charged for figures created on this account if they are not related to this manuscript submission.

LANGUAGE EDITING AND SUPPORT FOR PUBLICATION: If you would like help with English language editing, or other article preparation support, Wiley Editing Services offers expert help, including English Language Editing, as well as translation, manuscript formatting, and figure formatting at www.wileyauthors.com/eeo/preparation. You can also find resources for Preparing Your Article for general guidance about writing and preparing your manuscript at www.wileyauthors.com/eeo/prepresources.

RIGOUR AND REPRODUCIBILITY: We recommend authors consult The Journal's Rigour and Reproducibility requirements to ensure that their manuscript is suitable for publication: https://physoc.onlinelibrary.wiley.com/pb-assets/hub-assets/physoc/documents/TJP-Rigour-and-Reproducibility-Requirements.pdf

REVISION CHECKLIST:
Check that your Methods section conforms to journal policy: https://jp.msubmit.net/cgi-bin/main.plex?form_type=display_requirements#methods

Check that data presented conforms to the statistics policy: https://jp.msubmit.net/cgi-bin/main.plex?form_type=display_requirements#statistics

Upload a full Response to Referees file. To create your 'Response to Referees' copy all the reports, including any comments from the Senior and Reviewing Editors, into a Microsoft Word, or similar, file and respond to each point, using font or background colour to distinguish comments and responses and upload as the required file type.

Please upload two versions of your manuscript text: one with all relevant changes highlighted and one clean version with no changes tracked. The manuscript file should include all tables and figure legends, but each figure/graph should be uploaded as separate, high-resolution files. The journal is now integrated with Wiley's Image Checking service. For further details, see: https://www.wiley.com/en-us/network/publishing/research-publishing/trending-stories/upholding-image-integrity-wileys-image-screening-service

You may also upload:
- 'Potential Cover Art' for consideration as the issue's cover image
- Appropriate Supporting Information (Video, audio or data set: see https://jp.msubmit.net/cgi-bin/main.plex?form_type=display_requirements#supp).

We look forward to receiving your revised submission.

If you have any queries, please reply to this email and we will be pleased to advise.

Yours sincerely,

Bjorn Knollmann
Senior Editor
The Journal of Physiology

REQUIRED ITEMS

- You must start the Methods section with a paragraph headed Ethical approval (https://jp.msubmit.net/cgi-bin/main.plex?form_type=display_requirements#methods).

Research must comply with The Journal's policies regarding animal experiments (https://physoc.onlinelibrary.wiley.com/hub/animal-experiments) and adherence to these policies must be stated in the manuscript.

Authors should confirm in their Methods section that their experiments were carried out according to the guidelines laid down by their institution's animal welfare committee, including an ethics approval reference number. The Methods section must contain a statement about access to food, water and housing, details of the anaesthetic regime: anaesthetic used, dose and route of administration, and method of killing the experimental animals.

EDITOR COMMENTS

Reviewing Editor:

The second revision of your manuscript was reassessed by the expert referee who pointed out a number of remaining open issues including:

- Discussion of the rationale for changing key model parameters between revisions

- Discrepancies between Figures 7 and 10

- Many comments point out the need for rewording with attention to detail

- Fix non-resolving DOI and broken link to the code repository

Please also see 'Required Items' above.

Senior Editor:

I concur with the reviewing editor. I would like to emphasize to the authors to carefully address the concerns, which were already raised in the first review. If the revision is again unsatisfactory, the manuscript will be rejected.

REFEREE COMMENTS

Referee #2:

EVALUATION

- GENERAL

First of all, I would like to apologize for my late response. I received the request to review this R2 version of the manuscript on the very first day of a period of the academic year that is always extremely busy, with several courses running in parallel, each with their own coordination, lecturing, and exam obligations.

I have carefully read the updated manuscript and came across several inconsistencies-both within the manuscript itself and between the manuscript and the accompanying response letter-that require your attention. Some of these need an explanation. As with the previous version of the manuscript, it surprises me that none of the six authors noted these inconsistencies. In addition, I have some suggestions.

SPECIFIC

- Title

The title of the manuscript has been changed to "A Zebrafish Ventricular Action Potential Mathematical Model Integrating Whole-Heart Voltage and Calcium Measurements and Single-cell Electrophysiology". Why not a simple and more attractive title like "Computer Model of a Zebrafish Ventricular Cardiomyocyte"?

- Key Points

Only now I realize that "The model reinforces experimental findings, highlighting a key role of calcium currents in the action potential upstroke and intracellular calcium transient." can easily be misinterpreted. One may understand that multiple (sarcolemmal) calcium currents play a key role in the action potential upstroke as well as in the intracellular calcium transient, whereas it is the T-type calcium current that contributes to the action potential upstroke and the L-type calcium current that contributes to the intracellular calcium transient. This misinterpretation can be resolved by some straightforward rephrasing, as you already did for the abstract.

- Key Points

The "key role of calcium currents in the action potential upstroke" feels at odds with the Results section, where it reads: "the contribution of voltage-gated Na^+ channels in zebrafish ventricular CM, which are responsible for the rapid upstroke of the AP (Phase 0) and influence the speed of impulse transmission across the heart, is evident".

- Abstract Figure

Now that it has become clear that your manuscript contains newly obtained patch clamp data, I would expect this part of your study to be highlighted in the Abstract Figure as well. However, it is not. Consider updating, also paying attention to font size: if printed at 100% using a high-quality laser printer, the text in this figure is barely legible (as it is with some of the other figures).

- Keywords

Now that it has become clear that your manuscript contains newly obtained patch clamp data, I would expect 'patch clamp' or something similar among your keywords.

- Introduction

1) Lines 142-143: "partly driven by reverse-mode (Na+)-Ca2+ exchanger current". Is there a specific reason to use parentheses? Anywhere in the manuscript, it reads "Na+-Ca2+ exchanger current", without the parentheses.

2) Line 152: "no zebrafish-specific models exist". What about your own (preliminary) models and the accompanying two four-page papers that are listed in the References as well as the study by Qian & Tarte that is not (Qian S, Tarte E. Finite element modelling of discontinuous action potential propagation in larval zebrafish and human cardiac tissue. Phys Biol. 2019;17:016001)? Consider rewording.

- Methods

1) Lines 236-237: "Isolated cells were placed in a recording chamber on the stage of an inverted microscope (Diaphot, Nikon) and superfused with HEPES-buffered saline ICaL measurements." It seems that this sentence requires some rewording.

2) Line 247: "and was calculated as 27.2 {plus minus} 8.8 pF (n = 6)." Now that the use of standard deviation (SD) is only introduced in the next section, it requires some explanation here that 8.8 is the SD. One option is to move "Data are presented as mean {plus minus} standard deviation (SD)." from the next page to over here.

3) Lines 343-344: "figures showing all individual data points are available on Figshare (DOI: https://doi.org/10.6084/m9.figshare.27997817)." It seems that this DOI cannot (yet) be found in the DOI system. Is this on purpose?

4) Lines 382-383: "Istim is the externally applied stimulation current". I may have overlooked it, but could some information be included on the amplitude and duration of this stimulus current?

5) Lines 406-409: "The parameters used for the computational simulations are reported in Appendix 2 and the scripts for the MATLAB models and simulations, along with a CellML version of the 0D model, are available on GitHub (https://github.com/jfrodrig1971/Zebrafish)." It seems that these are not (yet) available on GitHub. Is this on purpose?

6) Lines 421-422: "First, the biophysical properties of the principal ionic currents were reparametrised using published and measured voltage-clamp data from zebrafish (as described in Section 2.8)." It seems to me that this reparameterization is described in Section 2.10 rather than in Section 2.8.

7) Line 430: "demonstrated, firstly, the absence of alternants in the last ten action potentials." I would write "alternans" instead of "alternants".

8) Line 456: "principal AP characteristics (i.e., RMP, APA, APD20-90," What is meant by "APD20-90"? It seems that it does not appear elsewhere in the manuscript. Do you perhaps mean APD20-APD90, or, more precisely, APD20, APD50, APD80,

and APD90, as listed in Table 2?

9) Lines 476-478, Table 1: the IC50 values are listed without mentioning the unit in which they are expressed. Supposedly, they are expressed in µM.

10) Lines 542-545, formulation of the T-type calcium current: ECaT appears in Equation 6 as well as in the associated text, with a reference to Appendix 1. However, in Appendix 1, the formulation of the T-type calcium current is with ECa rather than ECaT. Please correct.

11) Lines 699-702: "Finally, regarding the volumes of the various intracellular compartments, the cytoplasmic volume Vc is set to 390 µm3, following reported dimensions of the zebrafish ventricular CM (Brette et al., 2008) and assuming that Vc corresponds to 40% of the cell volume." I am confused here. In the original manuscript, I read: "Finally, regarding the volumes of the various intracellular compartments, the cytoplasmic volume Vc is set to 0.0024 µm3, following the dimensions of the zebrafish cardiomyocyte (Brette et al., 2008)". In the R1 version, it said: "Finally, regarding the volumes of the various intracellular compartments, the cytoplasmic volume Vc is set to 900 µm3, following reported dimensions of the zebrafish ventricular CM (Brette et al., 2008) and assuming that Vc corresponds to 50% of the cell volume." So, the cytoplasmic volume varies from 0.0024 through 390 to 900 µm3 between different versions of the manuscript, each time based on the same dimensions reported by Brette et al. (2008). Of note, I only now realize that it was changed from 0.0024 µm3 in the original manuscript to 900 µm3 in the R1 manuscript without specifying why. This requires a thorough explanation. At the same time, the rationale of the assumption that Vc corresponds to 50% (revision #1) or 40% (revision #2) of the cell volume could be explained. At the same time, the capacitive area, Ac, was changed from 3470 µm3 in the revised version of the manuscript to 1500 µm2 in the present version.

12) Lines 704-706: "The capacitive area, Ac, is assumed to be twice the surface area of the cell, as in (O'Hara et al., 2011), with the surface area of the cell set to 1500 µm2, corresponding to the lower bound value reported for the zebrafish (Brette et al., 2008)." If I understand correctly, this implies that the single cell capacitance amounts to 30 pF, as mentioned in the response letter. This nicely fits the experimental value of 27.2 {plus minus} 8.8 pF observed in the patch-clamp experiments (data included at line 247 of the present manuscript). It would be nice to point the reader to this similarity.

- Results

1) Line 711: "during steady-state pacing at 2-Hz pacing". Change to "during steady-state pacing at 2 Hz".

2) Lines 728-729: "Isolated hearts from-six experimental animals were used". Remove the underscore.

3) Figure 10: It would be helpful to include a panel with the associated action potentials, so that the current traces can readily be interpreted in relation to the action potentials.

4) Figure 10: How should Figure 10F be interpreted versus Figure 7? Figure 10F shows an IK1 trace during the action potential with a maximum amplitude that is well above 1.0 µA/µF, whereas the computational current-voltage relationship of Figure 7 demonstrates a maximum amplitude of IK1 near 0.7 µA/µF, as becomes clear in the newly included inset. This requires some explanation.

5) Lines 806-807: "figures showing individual experimental curves are available on Figshare (DOI: https://doi.org/10.6084/m9.figshare.27997817)". It seems that this DOI cannot (yet) be found in the DOI system. Is this on purpose?

6) Lines 883-886: "In the model, the IKs:IK1 maximal conductance ratio (GKs:GK1) is 1:5, while the IKs:IKr maximal conductance ratio (GKs:GKr) is 1:12, in agreement with reports from (Hassinen et al., 2015b, 2015a; Abramochkin et al., 2018), 2018). Yet, despite the relatively small IKs, it still has a large effect on repolarisation behaviour." I tend to disagree that IKs is "relatively small" as compared to IKr and IK1. The conductance ratios may suggest so, but the amplitudes of these three currents during an action potential are highly similar, as becomes clear from Figure 10 (panels D, E, and F). This needs rephrasing, together with the related text in the Discussion section.

7) Line 918: "by increasing Irel". To which extent?

- Discussion

Lines 1058-1065: "Considering the limitations of our study, it is important to recognise that direct measurements of some currents, such as INaK and INaCa, in adult zebrafish are lacking. Therefore, the original formulations from the TP04 model were utilised, and their validity was evaluated through specific biophysical properties of the model. In particular, the ability of the model to stabilise intracellular Na+ and K+ at different stimulation frequencies was verified. At a pacing rate of 2 Hz, after 100 s, intracellular Na+ stabilised at 7.99 mM, and intracellular K+ stabilised at 112.47 mM (see Figure A1 and Table A2 in Appendix 2)." But what if IKs is not treated as if it is a pure K+ current, thus not ignoring its Na+ component? From your response letter I conclude that you are not willing to correct this error, but you could at least bring it up as a limitation here.

- Appendix 2

Figure A1 is supposed to show that "Numerical concentrations [Na+] and [K+] stabilised during time in the model", but both panels show thick lines as if these concentrations exhibit considerable variations over time. This needs to be elucidated.

- References

Unfortunately, and in contrast with what is stated in the response letter, the list of references is still sloppy. Titles of papers lack subscripts, superscripts, and italics. The paper by Kopton et al. lacks a volume and article number (9, 1806), as already noted in my previous comments. I even provided the volume and article number. In alphabetical order, the Ten Tusscher papers should be listed before the Tomek et al. paper, not after.

END OF COMMENTS

EDITOR COMMENTS

Reviewing Editor:

The second revision of your manuscript was reassessed by the expert referee who pointed out a number of remaining open issues including:
- Discussion of the rationale for changing key model parameters between revisions
- Discrepancies between Figures 7 and 10
- Many comments point out the need for rewording with attention to detail
- Fix non-resolving DOI and broken link to the code repository

Thank you for once again reviewing and considering our revised manuscript and noting the remaining issues. We apologise for not adequately explaining the rationale for changing some key model parameters between revisions in our previous response, which has been necessary as we have responded to the reviewer's concerns – we hope the explanations presented here are now sufficient to understand these changes. We are also sorry for the misunderstanding that has caused what appears to be a discrepancy – we hope the revised text is now clear enough to avoid this misconception. And we appreciate the reviewer's continuing meticulous consideration of our text, and the suggestions made for rewording, which have allowed us to make our manuscript even clearer and precise. Regarding the non-resolving DOI and broken link to the code repository – as explained in our last response to the reviewer – we were purposefully leaving these links as unpublished until the paper was accepted, and instead attached the required information as additional files with our resubmitted manuscript. It appears these files were not seen by the reviewer, so we have now published the DOI links so everything will be easily accessible.

Senior Editor:

I concur with the reviewing editor. I would like to emphasize to the authors to carefully address the concerns, which were already raised in the first review. If the revision is again unsatisfactory, the manuscript will be rejected.

Thank you for your continued consideration of our manuscript. We have carefully addressed the new and recurring concerns of the reviewer and hope that our paper is now acceptable for publication in The Journal of Physiology.

REFEREE COMMENTS

Referee #2:

EVALUATION

- GENERAL

First of all, I would like to apologize for my late response. I received the request to review this R2 version of the manuscript on the very first day of a period of the academic year that is always extremely busy, with several courses running in parallel, each with their own coordination, lecturing, and exam obligations.
I have carefully read the updated manuscript and came across several inconsistencies - both within the manuscript itself and between the manuscript and the accompanying response letter -that require your attention. Some of these need an explanation. As with the previous version of the manuscript, it surprises me that none of the six authors noted these inconsistencies. In addition, I have some suggestions.

Thank you for once again carefully considering our manuscript. We apologise for the inconsistencies and hope that this revision and the explanations in the accompanying response will clear up any final misunderstandings.

SPECIFIC

Title

1) The title of the manuscript has been changed to "A Zebrafish Ventricular Action Potential Mathematical Model Integrating Whole-Heart Voltage and Calcium Measurements and Single-cell Electrophysiology". Why not a simple and more attractive title like "Computer Model of a Zebrafish Ventricular Cardiomyocyte"?

The current title was meant to satisfy the previous suggestion from the reviewer that it should represent elements of the experimental work presented in the manuscript. But we agree that a simple and more attractive title would be best, so we have changed it to: "**Mathematical Model of the Zebrafish Ventricular Cardiomyocyte Action Potential and Calcium Transient**".

Key Points

2) Only now I realize that "The model reinforces experimental findings, highlighting a key role of calcium currents in the action potential upstroke and intracellular calcium transient." can easily be misinterpreted. One may understand that multiple (sarcolemmal) calcium currents play a key role in the action potential upstroke as well as in the intracellular calcium transient, whereas it is the T-type calcium current that contributes to the action potential upstroke and the L-type calcium current that contributes to the intracellular calcium transient. This misinterpretation can be resolved by some straightforward rephrasing, as you already did for the abstract.

We agree that this could be misinterpreted as currently stated, so we have revised the phrasing to make it clearer.

3) The "key role of calcium currents in the action potential upstroke" feels at odds with the Results section, where it reads: "the contribution of voltage-gated Na+ channels in zebrafish ventricular CM, which are responsible for the rapid upstroke of the AP (Phase 0) and influence the speed of impulse transmission across the heart, is evident".

We see how these statements could be confusing. In the second statement, we did not mean to imply that voltage-gated Na^+ channels are *solely* responsible for the upstroke of the AP, so have changed that part of the text to instead read: "*critical for the rapid upstroke of the AP* ", as those channels primarily determine the speed of the AP upstroke, and as a result also influence the speed of impulse transmission. Calcium currents were found to be an important depolarising current supporting the end of the AP upstroke and the AP plateau. To make this clearer, the first phrase has been reworded to read: "*The model reinforces experimental findings, highlighting key roles of T- and L-type calcium currents in sustaining action potential depolarisation and the intracellular calcium transient*".

Abstract Figure

4) Now that it has become clear that your manuscript contains newly obtained patch clamp data, I would expect this part of your study to be highlighted in the Abstract Figure as well. However, it is not. Consider updating, also paying attention to font size: if printed at 100% using a high-quality laser printer, the text in this figure is barely legible (as it is with some of the other figures).

Thank you for this observation. The abstract figure now also includes a representation of the patch clamp data that was obtained in our study. We have also increased the font size in many of the figures to make the text more legible.

Keywords

5) Now that it has become clear that your manuscript contains newly obtained patch clamp data, I would expect 'patch clamp' or something similar among your keywords.

Thank you for the suggestion, we have added "patch clamp" to the keywords.

Introduction

6) Lines 142-143: "partly driven by reverse-mode (Na+)-Ca2+ exchanger current". Is there a specific reason to use parentheses? Anywhere in the manuscript, it reads "Na+-Ca2+ exchanger current", without the parentheses.

$^{+}$)-Ca^{2+}", as this is the first mention of sodium, so it must be defined (as is the case for the first use of all other ions).

7) Line 152: "no zebrafish-specific models exist". What about your own (preliminary) models and the accompanying

two four-page papers that are listed in the References as well as the study by Qian & Tarte that is not (Qian S, Tarte E. Finite element modelling of discontinuous action potential propagation in larval zebrafish and human cardiac tissue. Phys Biol. 2019;17:016001)? Consider rewording.

While our previous conference proceedings were only brief reports about preliminary versions of our model, we have reworded lines 150-166 so that we no longer make the outright claim that no zebrafish-specific models exist. Regarding the finite element model of Qian & Tarte (2019), this includes only a phenomenological representation of the zebrafish AP, which is now mentioned in the text.

Methods

8) Lines 236-237: "Isolated cells were placed in a recording chamber on the stage of an inverted microscope (Diaphot, Nikon) and superfused with HEPES-buffered saline I_{CaL} measurements." It seems that this sentence requires some rewording.

Thank you for noting this typo – we added the word "for".

9) Line 247: "and was calculated as 27.2 ± 8.8 pF (n = 6)." Now that the use of standard deviation (SD) is only introduced in the next section, it requires some explanation here that 8.8 is the SD. One option is to move "Data are presented as mean ± standard deviation (SD)." from the next page to over here.

We have now defined that this measurement is presented as mean ± standard deviation (SD).

10) Lines 343-344: "figures showing all individual data points are available on Figshare (DOI: https://doi.org/10.6084/m9.figshare.27997817)." It seems that this DOI cannot (yet) be found in the DOI system. Is this on purpose?

As mentioned in our previous response, DOI links have been created for the GitHub repository (containing the CellML and MATLAB code for the 0D and 1D models) and for the FigShare repository (containing the experimental data), but were being kept private until publication (we instead included the model codes as additional files in the Journal's editorial system for review purposes, however it appears that these may not have been made available to the reviewer). For the reviewer's convenience, these DOI links have now been made public.

11) Lines 382-383: "I_{stim} is the externally applied stimulation current". I may have overlooked it, but could some information be included on the amplitude and duration of this stimulus current?

The stimulation amplitude and duration are now reported in line 393.

12) Lines 406-409: "The parameters used for the computational simulations are reported in Appendix 2 and the scripts for the MATLAB models and simulations, along with a CellML version of the 0D model, are available on GitHub (https://github.com/jfrodrig1971/Zebrafish)." It seems that these are not (yet) available on GitHub. Is this on purpose?

Please see the response to comment 10 regarding the DOI links.

13) Lines 421-422: "First, the biophysical properties of the principal ionic currents were reparametrised using published and measured voltage-clamp data from zebrafish (as described in Section 2.8)." It seems to me that this reparameterization is described in Section 2.10 rather than in Section 2.8.

Amended.

14) Line 430: "demonstrated, firstly, the absence of alternants in the last ten action potentials." I would write "alternans" instead of "alternants".

Amended.

15) Line 456: "principal AP characteristics (i.e., RMP, APA, APD_{20-90}," What is meant by "APD_{20-90}"? It seems that it

does not appear elsewhere in the manuscript. Do you perhaps mean APD_{20}-APD_{90}, or, more precisely, APD_{20}, APD_{50}, APD_{80}, and APD_{90}, as listed in Table 2?

Yes, the APD parameters evaluated are APD_{20}, APD_{50}, APD_{80}, and APD_{90}. These are all now listed in the text.

16) Lines 476-478, Table 1: the IC_{50} values are listed without mentioning the unit in which they are expressed. Supposedly, they are expressed in µM.

Amended.

17) Lines 542-545, formulation of the T-type calcium current: E_{CaT} appears in Equation 6 as well as in the associated text, with a reference to Appendix 1. However, in Appendix 1, the formulation of the T-type calcium current is with E_{Ca} rather than E_{CaT}. Please correct.

Corrected.

18) Lines 699-702: "Finally, regarding the volumes of the various intracellular compartments, the cytoplasmic volume Vc is set to 390 µm3, following reported dimensions of the zebrafish ventricular CM (Brette et al., 2008) and assuming that Vc corresponds to 40% of the cell volume." I am confused here. In the original manuscript, I read: "Finally, regarding the volumes of the various intracellular compartments, the cytoplasmic volume Vc is set to 0.0024 µm3, following the dimensions of the zebrafish cardiomyocyte (Brette et al., 2008)". In the R1 version, it said: "Finally, regarding the volumes of the various intracellular compartments, the cytoplasmic volume Vc is set to 900 µm3, following reported dimensions of the zebrafish ventricular CM (Brette et al., 2008) and assuming that Vc corresponds to 50% of the cell volume." So, the cytoplasmic volume varies from 0.0024 through 390 to 900 µm3 between different versions of the manuscript, each time based on the same dimensions reported by Brette et al. (2008). Of note, I only now realize that it was changed from 0.0024 µm3 in the original manuscript to 900 µm3 in the R1 manuscript without specifying why. This requires a thorough explanation. At the same time, the rationale of the assumption that Vc corresponds to 50% (revision #1) or 40% (revision #2) of the cell volume could be explained. At the same time, the capacitive area, Ac, was changed from 3470 µm3 in the revised version of the manuscript to 1500 µm2 in the present version.

We must apologise to the reviewer regarding this particular aspect of the paper. In the first version of the manuscript, the expressions for the intracellular ionic concentrations were written as:

$$\frac{dNa_i}{dt} = -\frac{I_{Na} + I_{bNa} + 3I_{NaK} + 3I_{NaCa}}{V_c F}$$

$$\frac{dK_i}{dt} = -\frac{I_{K1} + I_{Kr} + I_{Ks} - 2I_{NaK} + I_{stim}}{V_c F}$$

$$\frac{dCa_{itotal}}{dt} = -\frac{I_{CaL} + I_{CaT} + I_{bCa} + I_{pCa} - 2I_{NaCa}}{2V_c F} + I_{leak} - I_{up} + I_{rel},$$

In revising the paper, we noted that these expressions were missing the term $C_m A_c$, which multiplies the first term on the righthand side of each equation, leading to the correct form (below), as reported in the first revised version of the manuscript:

$$\frac{dNa_i}{dt} = -\frac{I_{Na} + I_{bNa} + 3I_{NaK} + 3I_{NaCa}}{V_c F} C_m A_c$$

$$\frac{dK_i}{dt} = -\frac{I_{K1} + I_{Kr} + I_{Ks} - 2I_{NaK} + I_{stim}}{V_c F} C_m A_c$$

$$\frac{dCa_{itotal}}{dt} = -\frac{I_{CaL} + I_{CaT} + I_{bCa} + I_{pCa} - 2I_{NaCa}}{2V_c F} C_m A_c + I_{leak} - I_{up} + I_{rel},$$

Regarding the value of V_c, the model was calibrated using a fixed value of the ratio of $A_c/V_c = 0.13$ mm^{-1}, where A_c was assumed to be twice the surface area of the cell and V_c was considered as a fraction of the total cell volume. In most action potential models, this fraction typically ranges between 30% and 50% of the cell volume. To the best of the authors' knowledge, no studies have reported the cytoplasmic volume fraction for zebrafish, and only a few provide measurements of the zebrafish cardiomyocyte dimensions, from which cell surface area and volume can be estimated.

The values of A_c, V_c, and the cytoplasmic volume fraction in the model were selected to preserve the ratio of $A_c/V_c = 0.13$ mm^{-1}, but also always within a range consistent with cardiomyocyte dimensions reported in the literature. The differences in these parameter values between the first and second revision of the manuscript reflect an effort to align the model's effective membrane capacitance with the value measured in our patch-clamp experiments. Unfortunately, in the first review of the manuscript, the effective capacitance was wrongly calculated using the surface area instead of the capacitive area, leading to $C_m \approx 68$ pF. In the second revision, this mistake was corrected, and the values of A_c, V_c, and cytoplasmic volume fraction were selected such that $C_m = 30$ pF, in close agreement with the experimental measurements.

19) Lines 704-706: "The capacitive area, Ac, is assumed to be twice the surface area of the cell, as in (O'Hara et al., 2011), with the surface area of the cell set to 1500 µm2, corresponding to the lower bound value reported for the zebrafish (Brette et al., 2008)." If I understand correctly, this implies that the single cell capacitance amounts to 30 pF, as mentioned in the response letter. This nicely fits the experimental value of 27.2 {plus minus} 8.8 pF observed in the patch-clamp experiments (data included at line 247 of the present manuscript). It would be nice to point the reader to this similarity.

Thank you for this suggestion. This similarity is now pointed out in lines 725-727.

Results

20) Line 711: "during steady-state pacing at 2-Hz pacing". Change to "during steady-state pacing at 2 Hz".

Amended.

21) Lines 728-729: "Isolated hearts from-six experimental animals were used". Remove the underscore.

Amended.

22) Figure 10: It would be helpful to include a panel with the associated action potentials, so that the current traces can readily be interpreted in relation to the action potentials.

The associated action potential is now included in all panels, along with the current tracings, for direct comparison.

23) Figure 10: How should Figure 10F be interpreted versus Figure 7? Figure 10F shows an IK1 trace during the action potential with a maximum amplitude that is well above 1.0 µA/µF, whereas the computational current-voltage relationship of Figure 7 demonstrates a maximum amplitude of IK1 near 0.7 µA/µF, as becomes clear in the newly included inset. This requires some explanation.

The computational current-voltage relationship shown in Figure 7, which has a maximum amplitude of ~ 0.7 µA/µF, was generated by fitting the mathematical formulation for I_{K1} to patch clamp measurements by Hassinen et al. (2015) of I_{K1} in enzymatically isolated single cells (which is also shown in the figure). Numerous studies have reported the sensitivity of ion channels to enzymatic digestion during cell isolation (Yue et al, 1996; Li et al., 1996; Virag et al, 2001; Hoshimo et al, 2012). In a recent review by Varro et al. (2021), the authors state the following when referring to recordings in single cells using patch-clamp: "First, measurements are performed in single isolated myocytes or, occasionally, cell pairs, and it is uncertain how, and to what degree, different ion channels are influenced by individual enzymatic digestion during the isolation procedure. Therefore, even if the recordings show single-cell action potentials with a normal shape, the function of the finely regulated ion channels can be drastically altered from their original condition". As such, the experimentally measured maximum amplitude of I_{K1} in isolated zebrafish cells may be lower than in the intact zebrafish heart. To correct for this technical limitation, during the development of recent computational action potential models, the conductance of each ionic current (G_x) is

calibrated to reproduce experimentally recorded action potentials measured in tissue preparations, thus avoiding enzymatic isolation (*e.g.*, O'Hara et al., 2011; Tomek et al., 2019). In our case, we have followed a similar procedure in the development of our zebrafish-specific model.

The current tracing of I_{K1} seen in Figure 10F comes from simulations using our integrated cell model. As described in the paper, we ran Monte Carlo simulations (Section 2.7) to calibrate the conductances of each ionic current in the model to produce a similar action potential to that measured by microelectrodes in the whole heart. So, in our case, the final value of G_{K1} in the model, and the resulting maximum amplitude of the I_{K1} current in our simulations (which was above 1.0 µA/µF), reflects the value that generated an action potential that best matched those measured in the whole heart, so might be expected to be greater than that measured in enzymatically isolated cells. We have now included a discussion of this important consideration in lines 447-454 of the paper.

References:

Hoshino, S., Omatsu-Kanbe, M., Nakagawa, M. & Matsuura, H. (2012). Postnatal developmental decline in I_{K1} in mouse ventricular myocytes isolated by the Langendorff perfusion method: comparison with the chunk method. *Pflugers Arch* **463**(5), 649–668.

Li, G-R., Feng, J., Yue, L., Carrier, M. & Nattel, S. (1996). Evidence for two components of delayed rectifier K+ current in human ventricular myocytes. *Circ Res* **78**(4), 689-696.https://doi.org/10.1161/01.RES.78.4.689

Varro, A., Tomek, J., Nagy, N., Virág, L., Passini, E., Rodriguez, B. & Baczkó, I. (2021). Cardiac transmembrane ion channels and action potentials: cellular physiology and arrhythmogenic behavior. *Physiol* Rev **101(3)**, 1083-1176.https://doi.org/10.1152/physrev.00024.2019

Virag, L., Iost, N., Opincariu, M., Szolnoky, J., Szécsi, J., Bogáts, G., Szenohradszky, P., Varró, A. & Papp, J.G. (2001). The slow component of the delayed rectifier potassium current in undiseased human ventricular myocytes. *Cardiovasc Res* **49**(4), 790-797.https://doi.org/10.1016/S0008-6363(00)00306-0

Yue, L., Feng, J., Li, G.R. & Nattel S. (1996). Transient outward and delayed rectifier currents in canine atrium: properties and role of isolation methods. *Am J Physiol* **270** (6 Pt 2)**,** H2157–H2168.https://doi.org/10.1152/ajpheart.1996.270.6.H2157

24) Lines 806-807: "figures showing individual experimental curves are available on Figshare (DOI: https://doi.org/10.6084/m9.figshare.27997817)". It seems that this DOI cannot (yet) be found in the DOI system. Is this on purpose?

Please see the response to comment 10 regarding the DOI links.

25) Lines 883-886: "In the model, the I_{Ks}:I_{K1} maximal conductance ratio (G_{Ks}:G_{K1}) is 1:5, while the I_{Ks}:I_{Kr} maximal conductance ratio (G_{Ks}:G_{Kr}) is 1:12, in agreement with reports from (Hassinen et al., 2015b, 2015a; Abramochkin et al., 2018), 2018). Yet, despite the relatively small I_{Ks}, it still has a large effect on repolarisation behaviour." I tend to disagree that I_{Ks} is "relatively small" as compared to I_{Kr} and I_{K1}. The conductance ratios may suggest so, but the amplitudes of these three currents during an action potential are highly similar, as becomes clear from Figure 10 (panels D, E, and F). This needs rephrasing, together with the related text in the Discussion section.

We agree that the phrasing "*relatively small* I_{Ks}" could be misleading. We have revised the sentence (lines 907-911) to clarify that the I_{Ks} maximal conductance is relatively small compared to I_{Kr} and I_{K1}, while the total I_{Ks} current amplitude during the action potential is of similar magnitude to I_{Kr}, as shown in Figure 10. We also revised the following paragraph in the Discussion to make this explicitly clear (lines 1060-1061).

26) Line 918: "by increasing Irel". To which extent?

In lines 942-944 of the revised manuscript, we now clarify the extent of the increase in I_{rel}. Specifically, we increased the conductance of I_{rel} fivefold for a duration of 200 ms to simulate the caffeine-induced release of SR Ca^{2+} observed experimentally. This transient increase was sufficient to approximate complete SR calcium release, as in the related experiments involving perfusion with caffeine.

Discussion

27) Lines 1058-1065: "Considering the limitations of our study, it is important to recognise that direct measurements of some currents, such as I_{NaK} and I_{NaCa}, in adult zebrafish are lacking. Therefore, the original formulations from the TP04 model were utilised, and their validity was evaluated through specific biophysical properties of the model. In particular, the ability of the model to stabilise intracellular Na^+ and K^+ at different stimulation frequencies was verified. At a pacing rate of 2 Hz, after 100 s, intracellular Na^+ stabilised at 7.99 mM, and intracellular K^+ stabilised at 112.47 mM (see Figure A1 and Table A2 in Appendix 2)." But what if I_{Ks} is not treated as if it is a pure K^+ current, thus not ignoring its Na^+ component? From your response letter, I conclude that you are not willing to correct this error, but you could at least bring it up as a limitation here.

As stated in Section 2.10, our model does not treat I_{Ks} as a purely K^+-selective current in terms of its reversal potential; a small Na^+ permeability is included in its calculation. This approach was originally introduced by Luo CH and Rudy Y (*Circ. Res. 1991;68;1501-1526)*, and since adopted in most AP models, including TP04, TP06, O'Hara et al, 2011, and Tomek et al, 2019, among others. However, even though Na^+ permeability is included in the calculation of the reversal potential, none of these models account for Na^+ flux through I_{Ks} in the intracellular Na^+ balance equations. Nonetheless, we agree that this assumption should be explicitly acknowledged as a modelling limitation. We have now revised the paragraph (lines 1098-1105) to incorporate this point.

Appendix 2

28) Figure A1 is supposed to show that "Numerical concentrations [Na+] and [K+] stabilised during time in the model", but both panels show thick lines as if these concentrations exhibit considerable variations over time. This needs to be elucidated.

The apparent thickness in the traces of $[Na^+]$ and $[K^+]$ in Figure A1 reflects the fact that simulations involved electrical stimulation, such that intracellular concentrations of Na^+ and K^+ cyclically varied with each cardiac cycle between diastolic and systolic values (*i.e.*, the bottom and top of the 'thick' line). When the model is stimulated for a sufficient amount of time, the diastolic and systolic values of $[Na^+]$ and $[K^+]$ reach a steady state value, as apparent in Figure A1 when looking at the stability of the top and bottom of the thick line.
To make this more clear, we have now added insets to both panels in Figure A1, which zoom in on a short interval of representative paced activity, illustrating the nature of the fluctuations during each cardiac cycle and their stability over time. These insets help distinguish between the beat-to-beat variation in $[Na^+]$ and $[K^+]$ and their stability over time.

References

29) Unfortunately, and in contrast with what is stated in the response letter, the list of references is still sloppy. Titles of papers lack subscripts, superscripts, and italics. The paper by Kopton et al. lacks a volume and article number (9, 1806), as already noted in my previous comments. I even provided the volume and article number. In alphabetical order, the Ten Tusscher papers should be listed before the Tomek et al. paper, not after.

We apologise that the reviewer still found errors in the list of references (although 'sloppy' seems like an overly harsh interpretation). Most remaining errors unfortunately relate to apparent limitations of the reference software we are using (Zotero), which we have learned does not deal well with subscripts, superscripts, and italics. We have corrected all references (the best we can) and are comforted to know that the Journal's publisher will rebuild the reference list for publication and in the process will ensure that references all are formatted correctly with all required details included.

With regard to the ordering of the *ten Tusscher* and *Tomek* references, we have chosen to keep the current order, in which *ten Tusscher* follows *Tomek*. This is consistent with international cataloguing standards and library conventions, where particles such as *ten*, *van*, or *de* are not used as the basis for alphabetization. Instead, the main part of the surname determines sorting, meaning *ten Tusscher* is filed under "Tu" for *Tusscher*, not "te" for *ten*. This approach is outlined in the IFLA document "Names of Persons: National Usages for Entry in Catalogues" (IFLA Working Group on the International Standard Authority Data Number, 4th rev. & enlarged edition, 1996). We therefore believe the current ordering is correct and consistent with accepted referencing and cataloguing practices,

but will leave the final decision up to the Journal's publisher to align with their own conventions (which are not included in the Journal guidelines).

Dear Dr Cestariolo,

Re: JP-RP-2025-287624R3 "Mathematical Model of the Zebrafish Ventricular Cardiomyocyte Action Potential and Calcium Transient" by Ludovica Cestariolo, Zachary David Long, Arie O Verkerk, Jose Maria Ferrero, T Alexander Quinn, and Jose Felix Rodriguez Matas

We are pleased to tell you that your paper has been accepted for publication in The Journal of Physiology.

The last Word (or similar) version of the manuscript provided will be used by the Production Editor to prepare your proof. When this is ready you will receive an email containing a link to Wiley's Online Proofing System. The proof should be thoroughly checked and corrected as promptly as possible.

Authors should note that it is too late at this point to offer corrections prior to proofreading. Major corrections at proof stage, such as changes to figures, will be referred to the Editors for approval before they can be incorporated. Only minor changes, such as to style and consistency, should be made at proof stage. Changes that need to be made after proof stage will usually require a formal correction notice.

All queries at proof stage should be sent to: TJP@wiley.com.

Following copyediting and typesetting the accepted version will be published online.

Yours sincerely,

Bjorn Knollmann
Senior Editor
The Journal of Physiology

IMPORTANT POINTS TO NOTE FOLLOWING YOUR PAPER'S ACCEPTANCE:

If you would like to receive our 'Research Roundup', a monthly newsletter highlighting the cutting-edge research published in The Physiological Society's family of journals (The Journal of Physiology, Experimental Physiology, Physiological Reports, The Journal of Nutritional Physiology and The Journal of Precision Medicine: Health and Disease), please click this link, fill in your name and email address and select 'Research Roundup':
https://www.physoc.org/journals-and-media/membernews

• TRANSPARENT PEER REVIEW POLICY: To improve the transparency of its peer review process, The Journal of Physiology publishes online as supporting information the peer review history of all articles accepted for publication. Readers will have access to decision letters, including Editors' comments and referee reports, for each version of the manuscript as well as any author responses to peer review comments. Referees can decide whether or not they wish to be named on the peer review history document.

• You can help your research get the attention it deserves! Check out Wiley's free Promotion Guide for best-practice recommendations for promoting your work at: www.wileyauthors.com/eeo/guide. You can learn more about Wiley Editing Services which offers professional video, design, and writing services to create shareable video abstracts, infographics, conference posters, lay summaries, and research news stories for your research at: www.wileyauthors.com/eeo/promotion.

• IMPORTANT NOTICE ABOUT OPEN ACCESS: To assist authors whose funding agencies mandate public access to published research findings sooner than 12 months after publication, The Journal of Physiology allows authors to pay an Open Access (OA) fee to have their papers made freely available immediately on publication.

The Corresponding Author will receive an email from Wiley with details on how to register or log-in to Wiley Authors Services where you will be able to place an order.

• You can check if your funder or institution has a Wiley Open Access Account here: https://authorservices.wiley.com/author-resources/Journal-Authors/licensing-and-open-access/open-access/author-compliance-tool.html.

EDITOR COMMENTS

Reviewing Editor:

Your manuscript was assessed again by the expert referee who confirmed that their concerns have been addressed sufficiently.

Senior Editor:

The manuscript is acceptable for publication. Thank you for your excellent contribution!

REFEREE COMMENTS

Referee #2:

I thank the authors for carefully taking my comments into consideration and changing the manuscript accordingly. I realize that I tend to be a meticulous reviewer, but I feel that this has improved your manuscript.
